# Chromosome-scale assembly and evolution of the tetraploid *Salvia splendens* (Lamiaceae) genome

**DOI:** 10.1038/s41438-021-00614-y

**Published:** 2021-09-01

**Authors:** Kai-Hua Jia, Hui Liu, Ren-Gang Zhang, Jie Xu, Shan-Shan Zhou, Si-Qian Jiao, Xue-Mei Yan, Xue-Chan Tian, Tian-Le Shi, Hang Luo, Zhi-Chao Li, Yu-Tao Bao, Shuai Nie, Jing-Fang Guo, Ilga Porth, Yousry A. El-Kassaby, Xiao-Ru Wang, Charles Chen, Yves Van de Peer, Wei Zhao, Jian-Feng Mao

**Affiliations:** 1 Beijing Advanced Innovation Center for Tree Breeding by Molecular Design, National Engineering Laboratory for Tree Breeding, Key Laboratory of Genetics and Breeding in Forest Trees and Ornamental Plants, Ministry of Education, College of Biological Sciences and Technology, Beijing Forestry University, Beijing, 100083 China; 2 Ori (Shandong) Gene Science and Technology Co., Ltd, Weifang, 261000 Shandong China; 3 Départment des Sciences du Bois et de la Forêt, Faculté de Foresterie, de Géographie et Géomatique, Université Laval, Québec City, QC G1V 0A6 Canada; 4 Department of Forest and Conservation Sciences, Faculty of Forestry, University of British Columbia, Vancouver, BC V6T 1Z4 Canada; 5 Department of Ecology and Environmental Science, Umeå Plant Science Centre, Umeå University, SE-901 87 Umeå, Sweden; 6 Department of Biochemistry and Molecular Biology, 246 Noble Research Center, Oklahoma State University, Stillwater, OK USA; 7 Department of Plant Biotechnology and Bioinformatics, Ghent University, Ghent, Belgium; 8 VIB Center for Plant Systems Biology, 9052 Ghent, Belgium; 9 Centre for Microbial Ecology and Genomics, Department of Biochemistry, Genetics and Microbiology Genetics, University of Pretoria, Private Bag X20, Pretoria, 0028 South Africa; 10 College of Horticulture, Academy for Advanced Interdisciplinary Studies, Nanjing Agricultural University, Nanjing, 210095 China

**Keywords:** Structural variation, Plant evolution, Comparative genomics, Gene expression

## Abstract

Polyploidization plays a key role in plant evolution, but the forces driving the fate of homoeologs in polyploid genomes, i.e., paralogs resulting from a whole-genome duplication (WGD) event, remain to be elucidated. Here, we present a chromosome-scale genome assembly of tetraploid scarlet sage (*Salvia splendens*), one of the most diverse ornamental plants. We found evidence for three WGD events following an older WGD event shared by most eudicots (the γ event). A comprehensive, spatiotemporal, genome-wide analysis of homoeologs from the most recent WGD unveiled expression asymmetries, which could be associated with genomic rearrangements, transposable element proximity discrepancies, coding sequence variation, selection pressure, and transcription factor binding site differences. The observed differences between homoeologs may reflect the first step toward sub- and/or neofunctionalization. This assembly provides a powerful tool for understanding WGD and gene and genome evolution and is useful in developing functional genomics and genetic engineering strategies for scarlet sage and other Lamiaceae species.

## Introduction

Lamiaceae or Labiatae, or the mint family, is one of the largest families within the flowering plants, with 236 genera and more than 7000 species^1^. Plants in the mint family are chemically diverse, of great ecological, economic, and cultural importance, and extensively cultivated because of their ornamental value, flavor, fragrance, and medicinal properties. Composed of approximately 1000 species^1^, the genus *Salvia* is the largest genus of the mint family that seems to be polybasic, with different species having polyploid origins^2^. Tetraploid (2*n* = 4*x* = 44) scarlet sage (*Salvia splendens* or tropical sage)^3^ is among the most commonly cultivated ornamental plants and is characterized by dense flowers, wide color variation, long-lasting flowering (longer than 2 months), and resistance to pests and diseases^4^. *S. splendens* is a worldwide popular bedding plant with significant social and economic value^5^. Notwithstanding these virtues, genomic resources are only available for very few mint species, which severely limits further evolutionary and functional studies.

Polyploidization, also known as whole-genome duplication (WGD), is widespread across land plants and particularly frequent in ferns and angiosperms^6,7^, generating novel and varied phenotypes^8–10^. WGD events can result in instant reproductive isolation, as the difference in chromosome number impedes reproduction, promoting speciation, evolution, and biodiversity^11,12^. Polyploidization can enhance adaption, as it has been associated with survival in stressful environments^13^ (e.g., within aspen’s southwestern distribution in North America) (Goessen, Isabel, Giguère, Gros-Louis, Touchette, Laroche, Boyle, Lamothe, Tischenko, Soolanayakanahally, Mock, Bousquet, Hernández Velasco, Simental Rodriguez, Wehenkel, Porth (2021) in prep. How perennial angiosperms cope with environmental constraints: adaptive genetic variation and plasticity of life-history traits in *Populus tremuloides*). Gene duplications result in genetic redundancy, thereby increasing genetic resources and masking deleterious mutations through compensation. Polyploidization and initial redundancy also offer new possibilities for gene evolution: one copy can be degraded, both copies can be conserved by dosage balance, or their expression patterns may diverge (tissue-specific expression through subfunctionalization^14^ or even the evolution of new functions through neofunctionalization^15,16^). In such polyploids, the simultaneous duplication of many genes provides extra genetic material on which evolution can work, increasing genetic variation, which is considered an important mechanism for evolving adaptive traits^10,16^. However, although polyploidization may be significant in increasing genetic variation and affecting gene expression, we still have a limited understanding of the full extent of whether homoeologs resemble or differ from each other in their expression patterns, the spatiotemporal dynamics of these relationships, and how genomic rearrangement, transposable element (TE) proliferation, and sequence diversification impact these differences.

A draft genome sequence for *S. splendens* has been published previously^17^, but scaffold-level assembly complicates deeper investigation of polyploidy and homoeolog divergence and evolution. Here, based on long-read sequencing and the Hi-C scaffolding strategy, an 807 Mb chromosome-scale assembly was constructed for the tetraploid *S. splendens*, with 95% of the assembly anchored to 22 pseudochromosomes. We detected remnants of older WGD events and examined gene expression differences between homoeologs and potential associations with sequence diversification, genomic rearrangement, and TE proliferation. This genome assembly is essential to better understand the dynamic evolution following WGD in Lamiales; in addition, our findings will further benefit the functional genomics and molecular breeding of *S. splendens*.

## Results

### “Gold standard” tetraploid genome assembly and annotation

**Fig. 1:**
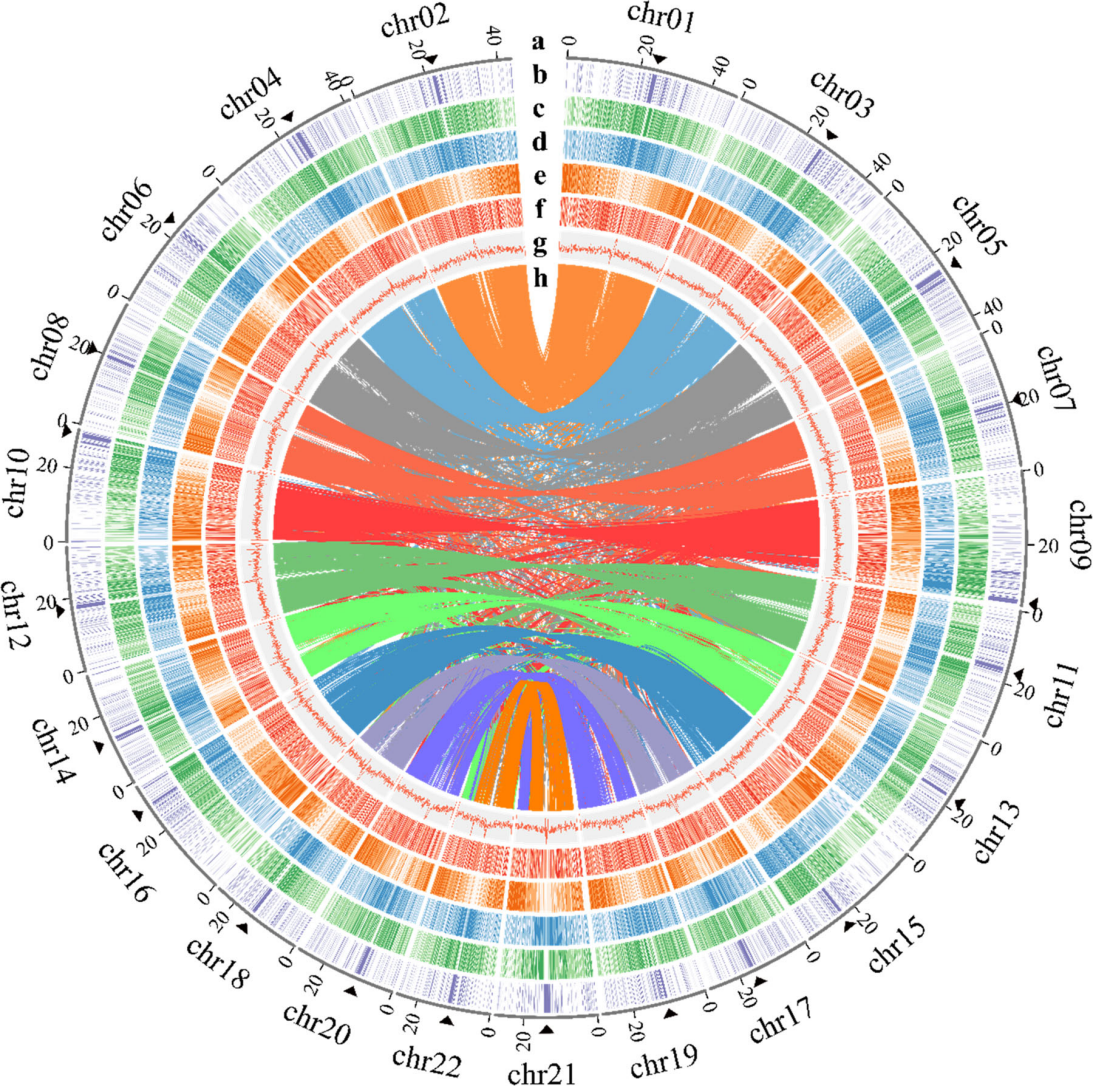
Synteny and distribution of features in the *S. splendens* genome. Chromosome numbers are indicated on the outside. Within the circle plot, the darker the color is, the higher the density. **a** Length (Mb) of chromosomes and position of the pericentromeric regions (black triangles), **b** LINE1 (long interspersed nuclear elements 1) density, **c** *Copia* density, **d** *Gypsy* density, **e** Gene density, **f** Pseudogene density, **g** GC (guanine-cytosine) content, and **h** Syntenic blocks

An elite tetraploid *S. splendens* cultivar, “aoyunshenghuo (Olympic flame),” developed through multiple rounds of selfing, was selected for sequencing. A total of 66 Gigabases (Gb) (82 X) of PacBio single-molecule long reads (average read length of 7.4 kb) and 37 Gb (40 X) Illumina paired-end reads were generated for initial assembly^17^ (Supplementary Table 1). A primary assembly with contig and scaffold N50 values of 2.27 and 3.12 Mb (Table 1, V1.0) was gained for further scaffolding after rounds of assembly, polishing, and comparison^17^. To anchor the scaffolds to chromosomes, we constructed high-throughput chromosome conformation capture (Hi-C) libraries of *S. splendens*, generating 85 Gb (105 X) Hi-C paired-end reads (Supplementary Table 1). As a result, the contig and scaffold N50 of the final assembly were upgraded to 3.77 and 35.13 Mb, with 47.13 Mb as the longest scaffold (Table 1, V2.0 and Supplementary Table 2). The final reference assembly comprised 22 chromosome-scale pseudomolecules representing 95% of the 807 Mb assembly (the pseudomolecules are hereafter referred to as chromosomes, Fig. 1 and Table 1). By filtering reads that showed sequence similarity to known chloroplast and mitochondrial genomes, we were able to assemble the chloroplast and mitochondrial genomes into single contigs with lengths of 150,607 and 347,308 bp, respectively (Supplementary Figs 1 and 2). The high-fidelity assembly was supported by a high ten-fold minimum genome coverage of 98.58% (PacBio) and high mapping rates of 99.06% (Illumina). Protein-coding regions were assembled to near completeness judged by the high Benchmarking Universal Single Copy Orthologs recovery of 92% in querying the embryophyte dataset^18^ (Supplementary Table 3). Repetitive sequence regions were assembled to high continuity by a high long terminal repeat (LTR) assembly index score of 27.49, reaching the “gold standard”^19^. These results suggest a highly accurate, very contiguous, and near-complete assembly.

**Table 1:** Statistics of the *S. splendens* genome assembly.

Assembly feature	V1.0	V2.0
Assembly size (Mb)	809.2	807.7
Anchored size (Mb)	–	767.09
Number of contigs	2204	1655
Max. contig length (Mb)	10.81	12.92
Contig N50 length (Mb)	2.27	3.77
Contig N90 length (Mb)	0.27	0.59
Number of scaffolds	1525	1184
Max. scaffold length (Mb)	12.94	47.13
Scaffolds N50 length (Mb)	3.12	35.13
Scaffolds N90 length (Mb)	0.43	25.62
GC content (%)	38.84	38.84
Repeat region % of assembly	57.52	56.94
Predicted gene models	54,008	88,489

We identified a total of 88,489 gene models, including 56,267 protein-coding genes, 28,993 long noncoding RNAs (lncRNAs), 1541 transfer RNAs (tRNAs), 518 ribosomal RNAs (rRNAs), and 1170 extra unclassifiable noncoding RNAs (ncRNAs) (Table 1 and Supplementary Table 4). There were 53,771 protein-coding genes (95.6%) that could be assigned to 22 chromosomes, and the gene density was highly skewed toward the distal region of chromosome arms (Fig. 1 and Supplementary Figs 3–24). Numerous transcription factor (TF; 3717) genes were predicted and classified into 56 gene families, including the major families bHLH, ERF, MYB, C2H2, NAC, and MYB_related, which contained 325, 321, 281, 221, 192, and 163 genes, respectively (Supplementary Table 5).

### Comparative phylogenomics and palaeopolyploidization

**Fig. 2:**
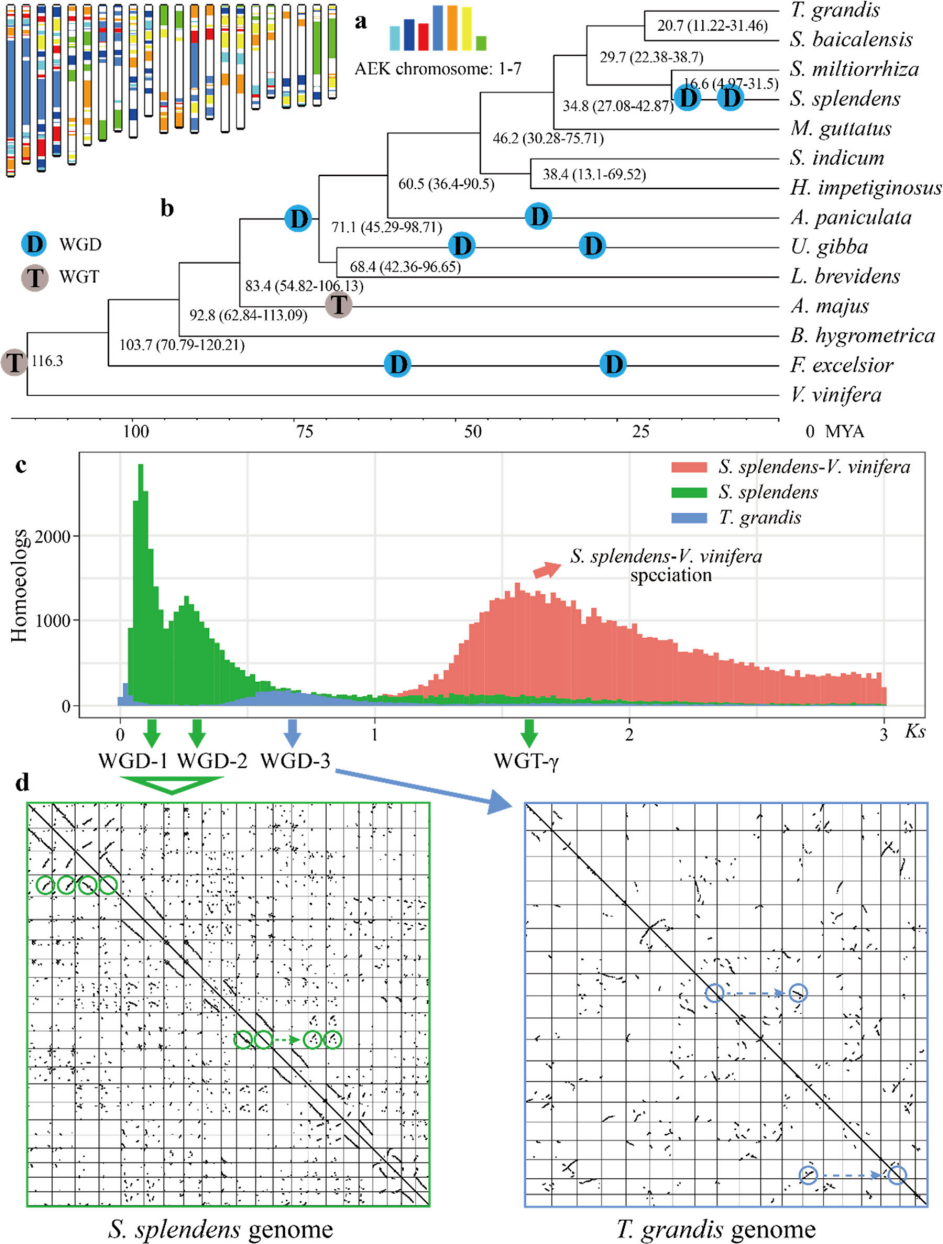
*S. splendens* evolutionary history. **a** Comparison with ancestral eudicot karyotype (AEK) chromosomes reveals synteny. The syntenic AEK blocks are painted onto *S. splendens* chromosomes. **b** Inferred phylogenetic tree, divergence time, whole-genome duplication (WGD), and whole-genome triplication (WGT). **c** *Ks* distribution. *y* axis, *S. splendens* paralogs (green), *T. grandis* paralogs (blue), and *S. splendens* – *V. vinifera* orthologs (red). Polyploidization (WGD-1, WGD-2, WGD-3 and WGT-γ) events are referenced on the *x* axis. **d** Dot plots of paralogs in *S. splendens* and *T. grandis* that illustrate WGD-1, WGD-2 (1:4 chromosomal relationships in green circles), and WGD-3 (1:2 chromosomal relationships in blue circles) events

To assess the palaeohistory of the Lamiales, we performed comparative genomic analyses incorporating *S. splendens* along with 12 Lamiales genomes and one outgroup (*Vitis vinifera*) (Fig. 2b, see Methods). Out of the 18,497 gene families (consisting of 364,467 genes), 557 genes from 88 gene families were found to be unique in the *S. splendens* genome (Supplementary Fig. 25). These unique genes are mainly related to plant resistance, such as the detection of biotic stimuli, hydrogen peroxide metabolism regulation, and salicylic acid biosynthesis (Supplementary Table 6). Construction of a phylogenetic tree from 373 orthogroups confirmed the evolutionary relationship within Lamiales, and the divergence between *S. splendens* and *Salvia miltiorrhiza* was estimated at 16.6 MYA (million years ago) (Fig. 2b).

Ancient polyploidization is widespread in most plant lineages, providing a powerful resource for novel genes and the evolution of new gene functions, as well as the emergence of new species^20^. Comparison of *S. splendens* with an ancestral eudicot karyotype genome^21^ and intragenomic homology supported WGD-1 (which led to sage’s tetraploid state) (Figs 1 and 2a). The *Ks* (the number of substitutions per synonymous site) distribution of intragenomic paralogs further showed two clear peaks of duplicate genes at *Ks* values of approximately 0.08 (WGD-1) and 0.26 (WGD-2) (Fig. 2c). The synteny analysis consistently revealed that these inferred WGD events (WDG-1 and WGD-2) in the *S. splendens* genome (Fig. 2d). Homoeology analysis of 11 pairs of homoeologous chromosomes indicated that they were highly conserved, despite few major rearrangements (Fig. 2d). Of the 53,771 protein-coding genes, 72% (40,246) were organized in macrosynteny, that is, still present at their ancestral genomic position and in intervals with a highly conserved gene order (Fig. 2d). The 1:4 syntenic relationship indicates that *S. miltiorrhiza* and *Tectona grandis* did not share these two WGD events (WGD-1 and WGD-2) with *S. splendens* (Supplementary Figs 26 and 27).

As shown in Fig. 2c, a peak near *Ks* = 0.6–0.8 (WGD-3) was identified by examining the intragenomic homology with *Mimulus guttatus*^22^, *Sesamum indicum*^23^, *Antirrhinum majus*^24^, *Tectona grandis*^25^, *Boea hygrometrica*^26^, and *Utricularia gibba*^27^. However, the *Ks* peak was not evident in *S. splendens* (Fig. 2c), which could be indicative of an accelerated substitution rate in *S. splendens*.

When analyzing the 1:1 syntenic relationship between *T. grandis* and *Scutellaria baicalensis*, *M. guttatus*, and *S. indicum*, the presence of the shared WGD-3 event emerged (Supplementary Figs 28–30). By combining phylogenetic analyses, we inferred that *Andrographis paniculate*, *Handroanthus impetiginosus*, *S. indicum*, *M. guttatus*, *S. miltiorrhiza*, *S. baicalensis*, *T. grandis*, and *S. splendens* all collectively shared WGD-3 (Fig. 2b). The 2:1 syntenic relationship between *Andrographis paniculata* and *S. indicum* revealed that, in addition to the WGD-3 event, *A. paniculata* might have experienced another independent WGD event (Supplementary Fig. 31). Together with the shared WGD found previously for *U. gibba* and *M. guttatus*^27^, these results collectively implicated that WGD-3 is a shared event among species of Lamiaceae (*T. grandis*, *S. baicalensis*, *S. miltiorrhiza*, *S. splendens*), Phrymaceae (*M. guttatus*), Pedaliaceae (*S. indicum*), Bignoniaceae (*H. impetiginosus*), Acanthaceae (*A. paniculate*), Lentibulariaceae (*U. gibba*), and Linderniaceae (*Lindernia brevidens*) (Fig. 2b).

Subsequently, we examined whether *A. majus* shared this WGD-3 with these species. Syntenic analysis showed that there was no 1:1 syntenic relationship supporting the same WGD-3 event shared between *A. majus*, *S. indicum*, and *T. grandis* (Supplementary Figs 32 and 33). In contrast, 3:2 syntenic blocks were identified between *A. majus, S. indicum*, and *T. grandis* (Supplementary Figs 32 and 33), suggesting that *A. majus* did not share the WGD-3 event with these species. Unexpectedly, 3:2 syntenic block support suggests that *A. majus* may have undergone a whole-genome triplication event rather than a WGD^24^.

### Recent accumulation of LTR-RTs and LINE1-dominated pericentromeres

Through an integrative approach, we identified 56.94% of the assembly as repeat elements (Supplementary Table 7). The long terminal repeat retrotransposons (LTR-RTs) form the largest proportion (26.66%) of repeats, while the most abundant LTRs were *Gypsy* elements, making up 17.59% of the genome, followed by *Copia* elements (8.70%; Fig. 1 and Supplementary Table 7). *Gypsy* and *Copia* families of repeats were found to have significantly contracted in *S. splendens* compared to *S. miltiorrhiza* (29.83%/*Gypsy* and 14.77%/*Copia* of the genome)^28^.

For full-length LTR-RTs, significant differences in individual counts, average length, and genomic coverage were present in the *Gypsy* and *Copia* superfamilies. Most LTR-RTs gradually accumulated during the last 5 million years, rather than showing accumulation by sudden bursts (Supplementary Fig. 34), possibly due to the relaxed purifying selection on TE overaccumulation after the last WGD (WGD-1)^29^. Abundant LTR-RTs overlapped with genes (Supplementary Figs 35 and 36), signifying the potential impacts of LTR-RTs on protein-coding genes. When comparing *S. splendens* to other Lamiales species with respect to LTR-RT accumulation and removal rates, we found that the *S. splendens* was characterized by large numbers of intact, solo, and truncated LTR-RTs and relatively low removal rates (Supplementary Fig. 37). These results implied that the recent expansion of LTR-RTs played a significant role in the genome size evolution of *S. splendens*.

Based on the Hi-C interaction, the pericentromeric position was identified for each chromosome using an established pipeline^30^ (Fig. 1 and Supplementary Figs 3–24 and 38). Interestingly, pericentromeric regions of *S. splendens* were enriched with long interspersed nuclear elements 1 (LINE1), along with an elevated guanine-cytosine (GC) content (Fig. 1 and Supplementary Figs 3–24). Therefore, we further defined the pericentromeric regions by examining LINE1 density and found that the size of centromeric regions ranged from 0.3 to 1.2 Mb and was delimited by 93–573 LINE1 elements (Supplementary Figs 3–24 and Supplementary Table 8).

### Structural variation between homoeologous chromosomes

Genomic structural variations (SVs) and/or rearrangements may occur through nonhomoeologous chromosome recombination between repetitive elements^31^. To test whether TEs are related to an increased rate of chromosome SVs, we first used the longest homoeologous chromosome as the reference and the shorter chromosome as a query to detect SVs between homologous chromosomes. In total, we identified 8036 large SVs, including 3053 duplications, 167 inversions, 865 translocations, 3160 inverted duplications, and 791 inverted translocations (Supplementary Figs 39 and 40 and Supplementary Tables 9 and 10). Furthermore, we randomly selected 1000 4 kb regions from each chromosome to compare the 2 kb regions upstream and downstream of SV breakpoints, and the results showed that TEs were significantly enriched within breakpoint regions (Supplementary Fig. 41). The TE content within breakpoint regions is nonrandom, with LTR being the most strongly enriched relative to other types of repeated elements (Supplementary Table 11). Our results suggest that TE insertions may provide substrates for *S. splendens* genome SV events.

In addition, we examined whether these different types of SVs have the same effects on the gene expression of homoeologs. Therefore, we identified genes related to SVs. A total of 18,031 genes were within the SV regions, of which 15,878 (88.06%) were in syntenic blocks shared between homoeologous chromosomes (Supplementary Table 12). Furthermore, we investigated genes that showed ≥2-fold significantly differential expression (*p* < 0.05) on homoeologous chromosomes in a pooled analysis by combining data across all RNA-seq samples (root, stem, leaf, calyx, and corolla for red/purple varieties; Supplementary Table 1). Inverted duplications (62.58%) and duplications (56.25%) had a greater impact on differentially expressed homoeologs than translocations (48.54%), inverted translocations (45.57%), and inversions (44.84%) (Supplementary Table 12). These results indicate that duplications had a significant effect on differential gene expression compared with other SVs, which may be because duplications can increase gene redundancy and render homoeologs more susceptible to mutation accumulation due to relaxed functional constraints on additional gene copies^32,33^.

### Spatiotemporal expression patterns of homoeologs

**Fig. 3:**
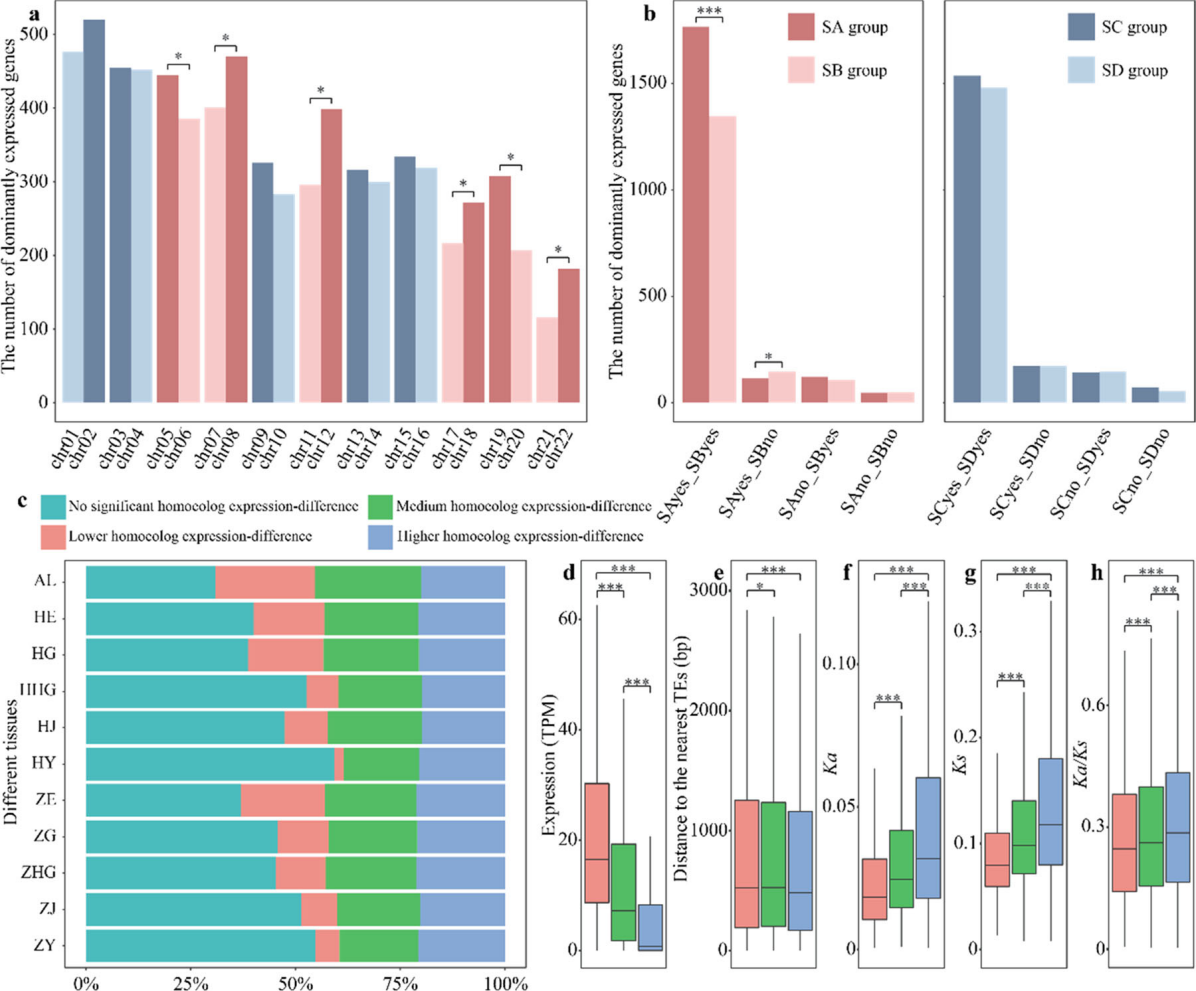
Biased gene expression of homoeologs. **a** The number of highly expressed genes across homoeologous chromosomes. **b** Expression effects of transposable elements (TEs) on flanking genes. Groups SA, SB, SC, and SD refer to chromosome groups categorized according to homoeolog expression bias and the number of highly expressed genes between homoeologous chromosomes. “Yes” indicates whether the gene category contains TEs within 2 kb; otherwise, “no.” **c** Proportion of homoeologs for each homoeologous expression bias category across the 10 tissues/varieties and pooled tissue. AL, pooled tissue; HE, calyx of the red variety; HG, root of the red variety; HHG, corolla of the red variety; HJ, stem of the red variety; HY, leaf of the red variety; ZE, calyx of the purple variety; ZG, root of the purple variety; ZHG, corolla of the purple variety; ZJ, stem of the purple variety; and ZY, leaf of the purple variety. **d** Absolute TPM gene expression abundance for the three homoeologous expression bias categories. **e** Distance to the nearest TEs for each of the three gene categories. **f**–**h** *Ka*, *Ks*, and the *Ka*/*Ks* ratio for each of the three gene categories. The bottom and top of each box represent the 25th and 75th percentiles, respectively. The centerline represents the 50th percentile. The whiskers indicate the minimum and maximum values. **d**–**h** Colors are consistent with cluster colors presented under (**c**). **a**, **b** *χ*^2^ test, **d**–**h** Mann–Whitney–Wilcoxon test. *p < 0.05; **p < 0.01; ***p < 0.001

In polyploids, the quantitative variation of many traits is regulated by genetic interactions. Among the 16,495 high-confidence homoeologs between homoeologous chromosome pairs (see Methods), 7481 (45.35%) exhibited significant ≥2-fold differential expression in the pooled tissue. Interestingly, we found that various homoeologous chromosome gene pairs showed different expression differences (Fig. 3a). Among the 11 homoeologous chromosome pairs, the number of two-fold expressed genes did not show significant differences in five pairs (chr01 vs. chr02; chr03 vs. chr04; chr09 vs. chr10; chr13 vs. chr14; and chr15 vs. chr16) of homoeologous chromosomes, while the remaining pairs of homoeologous chromosomes (chr05 vs. chr06; chr07 vs. chr08; chr11 vs. chr12; chr17 vs. chr18; chr19 vs. chr20; and chr21 vs. chr22) did show significant two-fold expression differences (Fig. 3a, *χ*^2^ test, *p* < 0.05). Unbalanced homoeologous expression bias was also seen for different varieties and tissues (Supplementary Fig. 42).

Based on whether or not homoeolog expression bias was detected and dependent on the number of highly expressed genes between homoeologous chromosomes (Fig. 3a), we separated all 22 *S. splendens* chromosomes into the following four groups: (1) SA (significant bias and more highly expressed genes, chr05; chr08; chr12; chr18; chr19; and chr22); (2) SB (significant bias and fewer highly expressed genes, chr06; chr07; chr11; chr17; chr20; and chr21); (3) SC (no significant bias and more highly expressed genes, chr02; chr03; chr09; chr13; and chr15); and (4) SD (no significant bias and fewer highly expressed genes, chr01; chr04; chr10; chr14; and chr16). We then investigated the impact of the flanking TE on the gene expression of the homoeologs. To do so, four categories were created: (1) SAyes_SByes, with both homoeologs having TEs within 2 kb; (2) SAyes_SBno, with the group SA genes having TEs within 2 kb and SB genes not; (3) SAno_SByes, with the group SB genes having TEs within 2 kb and SA genes not; and (4) SAno_SBno, where neither SA nor SB have TEs within 2 kb. The numbers of genes that exhibited significant two-fold expression changes in these four categories are shown in Fig. 3b. Group SA chromosomes exhibited significant expression dominance over group SB chromosomes when both SA- and SB-type genes had TEs within 2 kb (SAyes_SByes category). When comparing both chromosome groups, and when TEs were not in proximity to a gene (SAyes_SBno category), the number of dominantly expressed genes was slightly higher than when TEs were near genes (Fig. 3b). Furthermore, we performed a similar analysis on chromosome groups SC and SD that lack homoeolog expression bias. For gene categories such as those detailed above (SCyes_SDyes; SCyes_SDno; SCno_SDyes; and SCno_SDno), no significant differences were found (Fig. 3b). These results indicate that TEs affect the homoeologous expression bias pattern between homoeologous chromosomes.

Based on the level of homoeologous gene differential expression, we defined the following four homoeologous expression bias categories: (1) a lower homoeolog expression-difference with FC (fold-change) ≤|2| (*p* ≤ 0.05), (2) a medium homoeolog expression-difference with |2| < FC < |8| (*p* ≤ 0.05), (3) a higher homoeolog expression-difference with FC ≥ |8| (*p* ≤ 0.05), and (4) no significant homoeolog expression-difference (*p* > 0.05) categories. The differences in expression between most homoeologs were not significant, and the lower homoeolog expression-difference category showed a more dynamic change between different tissues, ranging from 2.3% in the leaf of the red cultivar (HY) to 23.8% in calyx of the purple cultivar (ZE) (Fig. 3c). We found that genes in the lower homoeolog expression-difference category were expressed across a wider range of tissues and had higher absolute transcript abundance (mean, 27.17 TPM) than genes in the medium expression-difference (mean, 21.68 TPM) and higher homoeolog expression-difference categories (14.56 TPM) (Fig. 3d). Absolute transcript abundance datasets indicated that the gene expression-difference between homoeologs could not be attributed to the increased expression of homoeologs but to the relatively low expression of a specific homoeolog. Next, we examined the associations of TEs with the relative expression of homoeologs and found that genes in the higher homoeolog expression-difference category were closer to the flanking TE than those in the medium homoeolog expression-difference and lower homoeolog expression-difference categories (Fig. 3e). We also compared the nonsynonymous (*Ka*) and synonymous (*Ks*) substitution rates between homoeologs and observed that genes in the higher homoeolog expression-difference category had higher *Ka*, *Ks*, and *Ka*/*Ks* than those in the medium homoeolog expression-difference and lower homoeolog expression-difference categories (Fig. 3f–h).

**Fig. 4:**
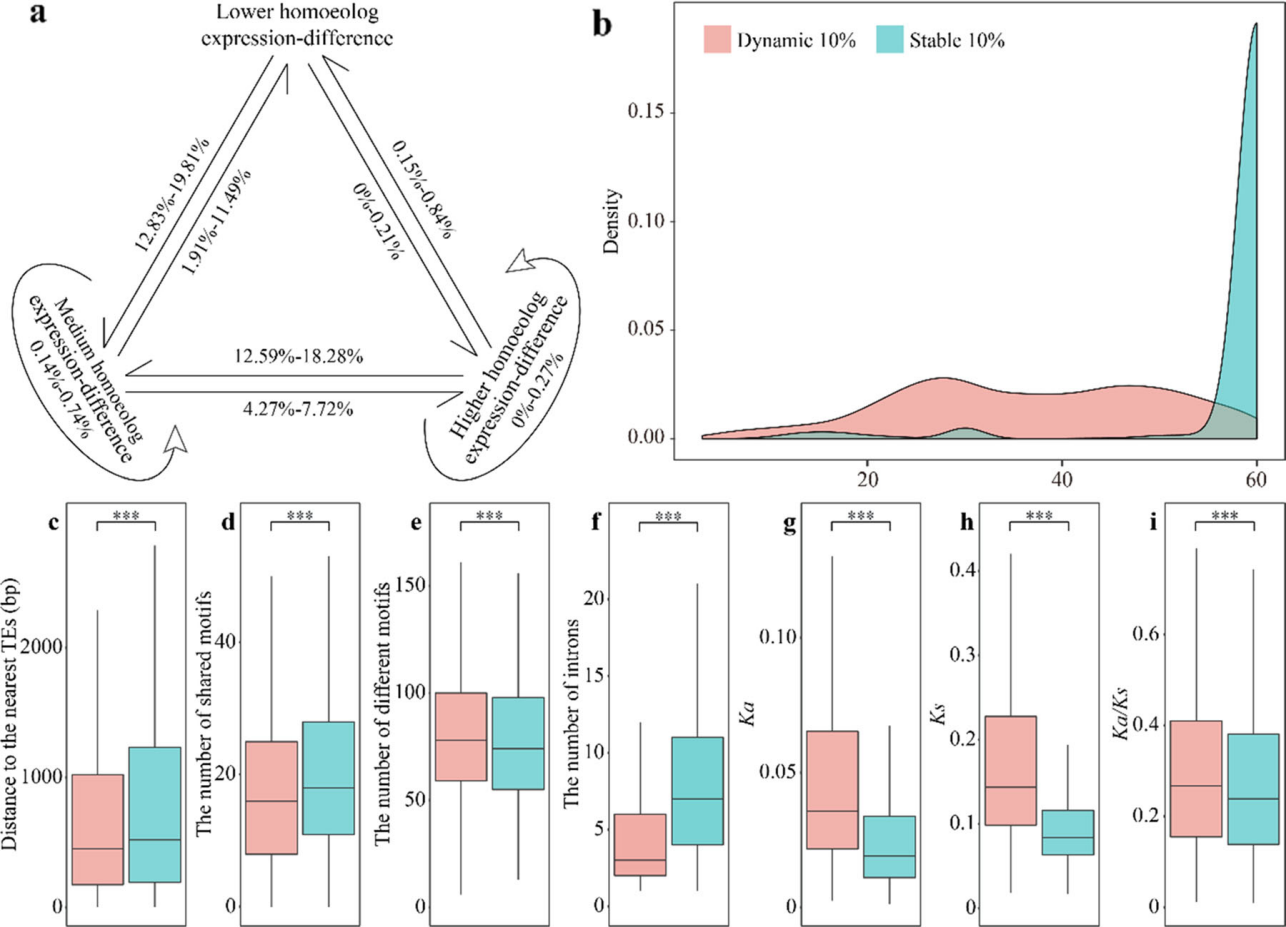
Stable and dynamic expression patterns of homoeologs. **a** Variations in the observed lower homoeolog expression-difference, medium homoeolog expression-difference, and higher homoeolog expression-difference categories. **b** Expression patterns across tissues querying dynamic vs. stable homoeologs. **c** Base pair distances to the nearest TEs comparing dynamic vs. stable homoeologs. TF binding site categories for dynamic vs. stable homoeologs: **d** number of shared motifs among dynamic vs. among stable homoeologs; **e** number of specific motifs among dynamic vs. among stable homoeologs. **f** Intron number per gene for dynamic vs. stable homoeologs. **g**–**i** *Ka*, *Ks*, and *Ka*/*Ks* ratio for dynamic vs. stable homoeologs. The bottom and top of each box represent the 25th and 75th percentiles, respectively. The centerline represents the 50th percentile. The whiskers indicate the minimum and maximum values. Colors are consistent with cluster colors presented under (**b**). Mann–Whitney–Wilcoxon test. ***p < 0.001

Polyploidy may confer phenotypic plasticity through homoeologs being differentially expressed among tissues and/or environmental conditions^16^. Therefore, we examined whether homoeologs retain their biased expression in different tissues (root, stem, leaf, calyx, and corolla for red/purple flower varieties; Supplementary Table 1). We found that most of the homoeologs remained in the original grouping, with only 0–19.81% (0–777 homoeologs) changing in at least one tissue (Fig. 4a). Adjacent categories (i.e., lower homoeolog expression-difference to medium homoeolog expression-difference or medium homoeolog expression-difference to higher homoeolog expression-difference) most often shifted to each other (1.91–19.81%, 80–777 homoeologs), with just 0–0.84% (0–33 homoeologs) of categories moving across categories (i.e., lower homoeolog expression-difference to higher homoeolog expression-difference) (Fig. 4a). These data indicate that homoeologs are usually expressed stably in different tissues.

Based on the dynamic change of differential expression between the single tissue and pooled tissue samples, we focused on the 10% most stable homoeologs with consistently stable expression and the 10% most dynamic homoeologs that show the highest variable expression in different tissues/varieties. Stable homoeologs had higher expression and higher expression widths and were expressed in almost all samples, while dynamic homoeologs were more tissue-specific (Fig. 4b). “Stable” homoeologs were enriched for gene ontology (GO) terms associated with RNA splicing, mRNA processing, and splicing via the spliceosome (Supplementary Table 13). In contrast, dynamic homoeologs were enriched for defense, secondary metabolic processes, and external stimuli responses, functions that more frequently determine fitness differences (Supplementary Table 14). Approximately 47% of stable homoeologs belonged to the lower homoeolog expression-difference category, whereas two-thirds of the dynamic homoeologs were part of the higher homoeolog expression-difference category. Compared to stable homoeologs, dynamic homoeologs were significantly closer to TEs and contained fewer conserved TF binding sites (Fig. 4c–e). We also found that the number of flanking TEs (within 2 kb) for dynamic homoeologs was on average higher (1.52 vs. 1.37), and dynamic homoeologs also contained fewer introns than stable homoeologs (Fig. 4f). Dynamic homoeologs had a significantly higher *Ka*, *Ks*, and *Ka*/*Ks* ratio, indicating that they have relaxed selection pressure (Fig. 4g–i).

These results indicate that spatiotemporal expression patterns are positively correlated with differences in flanking TEs, *cis*-regulatory elements, coding sequences, and selection pressure. These observed changes in spatiotemporal expression patterns, as well as the relaxation of selection pressure, the proximity of TE and the difference of TF binding sites, may lead to functional innovation through sub- or neofunctionalization, following, for instance, divergence of gene expression.

To further examine whether these homoeologs are coordinately expressed, we also constructed a weighted gene coexpression network for the red-flowered and purple-flowered varieties separately. For the red-flowered variety, these networks were composed of 26 modules and contained 98% of all expressed genes. We found 34% of the homoeologs in the same coexpression module (Supplementary Table 15), while the majority of homoeologs (66%) were in different modules. To quantify whether homoeologs in different modules have similar or divergent expression patterns, we calculated the Euclidean distance between module eigengenes. Homoeologs for which the pairwise distance was zero were in the same module. Homoeologs with distances greater than zero were in different modules. When the distance between homoeologs was greater than 50% of the median distance between eigengenes, the two genes were classified as having “divergent” expression patterns in different modules; otherwise, they had “similar” expression patterns. We found only 33% of homoeologs with a divergent pattern, suggesting a globally highly coordinated expression pattern (Supplementary Table 15). A similar coordinated expression pattern was also found for the purple-flowered variety (Supplementary Table 15). The “divergent” expression patterns in different modules foreshadow sub- or neofunctionalization between homoeologs^34^.

## Discussion

Plant genomes accommodate much more redundancy, diversity, and dynamics than animal genomes^35^. Owing to their sessile nature, plants are constantly exposed to a multitude of environmental stressors. Such genomic redundancy is often provided as the raw material allowing plants to develop relatively rapid functional innovations^36^. Polyploidization/WGD events, often accompanied by the expansion of large amounts of repeated genes, large genomic rearrangements, and increased genetic variation, are thus considered catalysts for species diversification and evolutionary novelty in plants^10,37–40^. A recently published phylotranscriptomic study presented compelling evidence of widespread ancient polyploidy in Lamiaceae, particularly within the species-rich and chemically diverse Nepetoideae, where *S. splendens* resides^41^. Our study provides important genomic resources to further understand the genetic and genomic dynamics related to WGD that contribute to specific trait innovations, novelties in specialized metabolism, and subsequent diversification.

Following a polyploidization event, one of the subgenomes, often referred to as the “dominant” subgenome, exhibits significantly higher gene content and greater gene expression than the “recessive” subgenome^42^. There is also growing evidence in polyploid plants for the nonadditive contribution of parental genomes to gene expression^43–47^. At the subgenome level, Thomas et al.^42^ provided details of “biased fractionation” of two progenitor genomes. Findings in cotton also suggested that ancient genomic fractionation persists and can influence the modern cotton genome’s functional space, despite approximately 60 million years of evolution^48^. When examining more recent hybridization events, this asymmetric gene expression favoring either one of the A, B, or D cotton subgenomes was absent, and instead, the expression dominance was region- and cell type-dependent, with up to 20–40% of homoeologous pairs exhibiting expression bias in developmental stages and tissues across endosperm development^49,50^. Similar patterns of localized expression bias were also observed in *Brassica napus*^51^, as well as in allotetraploid cotton^45^. As shown in Fig. 3a, our results at the subgenome level also suggest an inconsistent, biased expression pattern by resolving the expression biases to the homoeologs. Furthermore, although marginally significant, TE-mediated epigenetic silencing might lead to the repression of gene expression for the dominant subgenome in *S. splendens* (SAyes_SBno in Fig. 3b)^52^. However, when studying TE adjacency to homoeologs, this TE-mediated silencing effect was found to be more prevalent on “recessive” homoeologous chromosomes (Fig. 3b), suggesting an initial cause of chromosome degradation. As a key driver of mutagenesis in the genome, TEs are capable of inducing initial differences in gene expression among homeologs of the polyploid genome^52^. Together with the large numbers of intact, solo, and truncated LTR-RTs identified in this study (Supplementary Fig. 37), we speculate that the ineffective TE removal was attributed to the inconsistent expression biases in the *S. splendens* genome.

In addition to the WGD shared by most eudicots, we identified three *S. splendens* lineage-specific WGD events. Following each of the WGD events, the doubling of genome copies often leads to two nonmutually exclusive evolutionary scenarios owing to the inflated TE content^29,53^. Originally proposed by Barbara McClintock^54^, the widespread upregulation of TEs as a consequence of “genome shock” would facilitate alterations in gene expression, likely through epigenetic regulation such as DNA methylation, to maintain genome stability^55,56^. Alternatively, WGD may lead to a buffering effect on recessive deleterious mutations and loss-of-function mutations caused by TE insertion. Such relaxed selective pressure could readily promote TE overaccumulation without transposition bursts^29^.

Comparing expression and epigenetic profiles in parental and hybrid contexts, despite the major reorganization of genes and stress-induced TE expression, no evidence for global gene expression dysregulation caused by “genomic shock” can be concluded^57^. As depicted in Fig. 4a, stable, high expression for most of the homoeologs across tissue types was found, regardless of their TE adjacency (Fig. 4c). Conversely, the dynamic homoeologs, as the top 10% of the homoeologs that exhibited differential expression changes across tissue types, demonstrated a broad range of genetic expression and had significantly higher synonymous, nonsynonymous mutations and, more importantly, significantly relaxed selection pressure (Fig. 4g–i). Functional analysis of these dynamic homoeologs also implied their characteristics in defense, secondary metabolic processes, and external stress responses (Supplementary Table 14). In the present study, we provided our adaptive evolution viewpoints on the TE-driven functional differentiation of homoeologs in polyploid genomes^10,58,59^. Finally, our results support adaptability in polyploid genomes as a result of relaxed purifying selection on the increased standing variation of environmentally responsive and stress-related genes^20,60^.

In conclusion, our chromosome-scale high-quality assembly of *S. splendens* and genomic analyses provide essential resources to understand the dynamic evolution following WGD in Lamiales. In addition, our findings will further benefit the functional genomics and molecular breeding of *S. splendens*, such as genome editing practices.

## Materials and methods

### Plant material

The sequenced individual of *S. splendens* (Aoyunshenghuo) was collected from the Beijing Institute of Landscape Architecture germplasm bank. This variety was originally developed by multiple rounds of selfing of one hybrid.

### Hi-C library construction and scaffolding

Young leaves of individual plants from the same *S. splendens* variety were collected for Hi-C library construction with the Proximo^TM^ Hi-C Plant kit (Phase Genomics) following the manufacturer’s protocol. Briefly, fresh-leaf tissue (0.2 g) was chopped, and chromatin was immediately crosslinked, fragmented, and proximity ligated, followed by library construction. The final library was size selected to contain 300–600 bp fragments and sequenced on the Illumina HiSeq 2500 device under paired-end 150 bp mode. Adapters were trimmed, and low-quality sequences were removed using fastp v0.19.5^61^. Quality-filtered reads were aligned to the *S. splendens* contigs using bwa v0.7.12^62^ with strict parameters (-n 0) to prevent mismatches and nonspecific alignments in the Juicer v1.5.6 pipeline^63^. Read pairs were merged, and PCR duplicates were filtered prior to constructing the interaction based on the distance matrix. Chimeras were split, and contigs were ordered and oriented using the 3d-DNA pipeline with default parameters^64^. The resulting Hi-C contact matrix was visualized using Juicebox v180922^65^, contig misassemblies and scaffold misjoins were manually detected and corrected based on neighboring interactions. In total, this method identified 22 high-confidence clusters representing the haploid chromosome number in *S. splendens*. The manually validated assembly was used as input to build 22 pseudomolecules using the finalize-output.sh script from 3d-DNA. Chromosomes were renamed and ordered by size, and homoeologous chromosomes were numbered consecutively.

### Optimization of genome assembly

The genome assembly was gap closed twice using LR_Gapcloser v1.1^66^ with PicBio data. Then, we polished the assembly a third time using pilon v1.22^67^ with Illumina short reads. Contigs with an identity of more than 99% were regarded as redundant sequences and were removed. The final assembly of *S. splendens* was 807 Mb.

### Repeat annotation

De novo repeat identification was pursued with RepeatModeler v1.0.10 (http://www.repeatmasker.org/RepeatModeler/), which employs two complementary computational methods (RECON v1.08 and RepeatScout v1.0.5)^68^ for identifying repeat element boundaries and family relationships from sequence data. The consensus repeat sequences generated above were combined and used for further characterization of TEs with RepeatMasker v4.0.7 (http://www.repeatmasker.org/).

### Gene annotation

AUGUSTUS v3.2.3^69,70^ was employed for ab initio gene prediction. Then, the transcriptome assembly^17^ was aligned to the repeat-masked reference genome assembly with BLAST v2.2.28+^71^. Next, protein sequences from *A. thaliana*^72^, *S. miltiorrhiza*^73,74^, and *S. splendens*^17^ were aligned to the masked genome assembly with BlastX. After optimization using Exonerate v2.4.0^75^, the gene model was finalized for prediction using the MAKER package v2.31.9^76^ with AUGUSTUS v3.2.3. The quality of gene prediction was assessed using annotation edit distance for each of the predicted genes as part of MAKER. Pseudogenes were identified using Pseudopipe^77^ with default parameters. The programs tRNAScan-SE v2.0.5^78^ and RNAmmer v1.2^79^ were used to predict tRNA and rRNA, respectively, and other ncRNAs were identified by searching against the Rfam database (http://eggnogdb.embl.de/). The functions of each gene model were predicted by homoeology searches with BLAT v0.36^80^ against the UniProt database^81^. Protein annotation against Pfam^82,83^ and InterProScan v5.27–66.0^84^ was also conducted using the MAKER package. In addition, we mapped the predicted genes to GO and KEGG classifications.

### Centromere/pericentromeric detection

Putative centromere/pericentromeric regions were predicted based on corrected Hi-C data and the tendency of formed clusters in three-dimensional space with a published bioinformatic procedure^30^. The centromeric regions were further delimited by examining the distribution of LINE1.

### Full-length LTR-RT annotation

We used LTRharvest v1.5.8^85^ and LTRdigest^86^ for de novo prediction of LTR-RTs. The LTR-RT candidates that possessed complete *Gap-Pol* protein sequences were retained as intact LTR-RTs (*I*), while solo-LTRs (*S*) and truncated LTRs (*T*) were identified based on sequence similarity to the intact LTR-TRs (*E* value <1e–10, overlap length >90%, identity >90%). Then, LTR homoeology within 15 kb of sequence data both up- and downstream was extracted and compared with *Gap-Pol* protein sequences within the rexdb^87^ database using TBLASTN^71^. We considered the corresponding LTRs as truncated LTR-RTs if they had at least 50% *Gag-Pol* covered by one side of the flanking sequence and 30% identity. Lacking *Gag-Pol* up- and downstream of the LTR was considered to represent a solo-LTR.

### Differential proliferation, age dynamics, and gene proximity of different LTR-RT families

The insertion time of LTR-RTs was estimated according to the difference between the 5′-LTR and 3′-LTR of the same transposon^88^ using MAFFT v7.221^89^ with a mutation rate of 1.3e–8 substitutions year^−1^ per site. Although the actual pattern of LTR-RT activation and amplification appeared at the family level, as defined by >80% sequence homoeology in the LTR-RTs, our focus was on holistic genomic characteristics that can be more carefully dissected and compared at the LTR-RT superfamily level (>60% homoeology). Then, we calculated the distances between intact LTR-RTs and adjacent genes and examined the relationships of proximity to genes and insertion time.

To further understand the relationship among individual LTR-RTs, we aligned the 5′-LTR sequences of all LTR-RTs. If two LTRs covered at least 70% of the length of each other and had at least 60% identity, they were assigned to the same cluster^90^. We also compared Solo-LTR-RTs and truncated LTR-RTs to the same cluster containing the 5′-LTR from the most similar intact LRT-RTs. The ratios of solo-LTR-RTs and truncated LTR-RTs to intact LTR-RTs (*S*:*I*, *T*:*I*) and their sum were then evaluated separately to investigate the removal rate of LTR-RTs over the past several million. Furthermore, we evaluated the proportion of clusters with *S*:*I* values greater than 3.

### Molecular phylogenetic analysis

We performed a comparative genomic investigation of *S. splendens* with *A. paniculata*^91^, *A. majus*^24^, *B. hygrometrica*^26^, *Fraxinus excelsior*^92^, *H. impetiginosus*^93^, *L. brevidens*^94^, *M. guttatus*^22^, *S. miltiorrhiza*^73^, *S. baicalensis*^95^, *S. indicum*^96^, *T. grandis*^25^, and *U. gibba*^27^ as representatives of the Lamiales order and *Vitis vinifera* as the outgroup.

OrthoFinder2 v2.3.1^97^ was used to identify homoeologous gene clusters. Based on 373 orthogroups, IQ-TREE v1.6.7^98^ was used to build a phylogenetic tree. MAFFT v7.407^89^ was used to align homoeologs before transforming aligned protein sequences into codon alignment. The concatenated amino acid sequences were trimmed using trimAL v1.4^99^ with -gt 0.8 -st 0.001 -cons 60. The program MCMCTree of PAML v4.9h^100^ was used to estimate the neutral evolutionary rate with an independent substitution rate (clock = 2), GTR substitution model, 2.1e6 iterations, and discarded 1e5 iterations as burn-in and fossil date points from TimeTree (http://timetree.org): 110–124 MYA split time between *V. vinifera* and *F. excelsior*.

### Whole-genome duplication

Syntenic blocks containing at least five genes were identified using MCScanX^101^ with default parameters. KaKs_Calculator 2.0^102^ was used to calculate *Ka*, *Ks*, and the *Ka/Ks* ratio by implementing the YN model. *Ks* values >5.0 were excluded from all analyses due to saturated substitution as synonymous sites^103,104^.

### Homoeolog identification

We excluded syntenic blocks between nonhomoeologous chromosome pairs and retained only the gene pairs on the syntenic blocks between homoeologous chromosomes as homoeologs.

### Structure variation detection

The Nucmer alignment tool from the MUMmer v4.0.0 toolbox^105,106^ was used to perform whole-genome alignments. Nucmer was run with -maxmatch to obtain all alignments between two homoeologous chromosomes, including -c 500, -b 500, and -l 100 parameters. Subprograms *Delta-filter* and *show-coords* were employed to filter the alignments and convert them into tab-delimited files. Finally, SyRI^107^ was used to identify inversions, translocations, duplications, inverted translocations, and inverted duplications. We compared the number of TEs in the upper and lower 2k regions at the SV breakpoints and 22,000 4k regions (1000 segments per chromosome) randomly selected from the 22 chromosomes using BEDTools v2.29.2^108^. Similarly, we used BEDTools to compare the intersection of structural variant segments and genes.

### Mapping of RNA-seq reads

Data were collected for three independent biological replicates from five major tissues with two different varieties. Low-quality reads were removed, and the remaining reads were mapped to the genome using hisat2 v2.0.0^109^. We summarized expression levels from the transcript level to the gene level using featureCounts v1.5.3^110^. If the expression value of a gene or homoeolog exceeded 0.5 TPM in any sample, we considered them to be expressed genes or high-confidence homoeologs. Differentially expressed homoeologs were compared using the DESeq2 package^111^.

### Dynamic and stable homoeologs identification

We first identified homoeologs that were differentially expressed ≥2 (*p* < 0.05) in all tissues and pooled tissue. Then, we merged homoeolog sets. For each pair of homoeologs, if *p* ≥ 0.05 in a tissue, we defined them as no difference (FC = 0). Dynamic homoeologs were defined as the top 10% with the largest FC change between different single tissues and pooled tissues, and stable homoeologs were defined as the top 10% with the smallest FC change.

### Transcription factor (TF) and TF binding site identification

The protein sequence was submitted to plantTFDB^112^ to identify TFs with the best hit in *Arabidopsis thaliana*. The 2 kb sequence upstream of the gene was used to identify TF binding sites present in the promoters of genes. The FIMO tool from MEME suite v4.12^113^ was used with a position weight matrix obtained from plantTFDB to predict TF binding sites. FIMO was run with a *p* value threshold of 1e–05, -motif-pseudo of 1e–08 and a -max-stored-scores of 1e6.

### WGCNA network construction

Coexpression networks were separately built for each of the two varieties with RNA-seq gene expression data from five different tissues using the WGCNA R package^114^. The soft power threshold was calculated as the first power to exceed a scale-free topology fit index of 0.9 for each network separately. Signed hybrid networks were constructed blockwise using the function blockwiseModules. The topographical overlap matrices (TOM) were calculated by the blockwiseModules function using TOMType = “unsigned.” The parameter mergeCutHeight = 0.25 was used to merge similar modules.

### GO enrichment analysis

GO enrichment analysis was performed using the R package clusterProfiler^115^. The *p* values were adjusted for multiple comparisons using the method of Benjamini and Hochberg (*p* < 0.05 was considered significant).

## Supplementary information

Supplementary materials

## Data Availability

The Whole Genome Shotgun project has been deposited at DDBJ/ENA/GenBank under the accession PNBA00000000. The version described in this paper is PNBA00000000. The raw sequence data have been deposited in the Short Read Archive under NCBI BioProject ID PRJNA422035.
