# Supplementary material for: Chromosome-scale assembly and evolution of the tetraploid Salvia splendens (Lamiaceae) genome
Source: Hortic Res. 2021 Sep 1;8:177. doi: 10.1038/s41438-021-00614-y (PMC8408255; doi:10.1038/s41438-021-00614-y)
Supplement: Supplementary file 1 — Supplementary materials [file 41438_2021_614_MOESM1_ESM.docx]

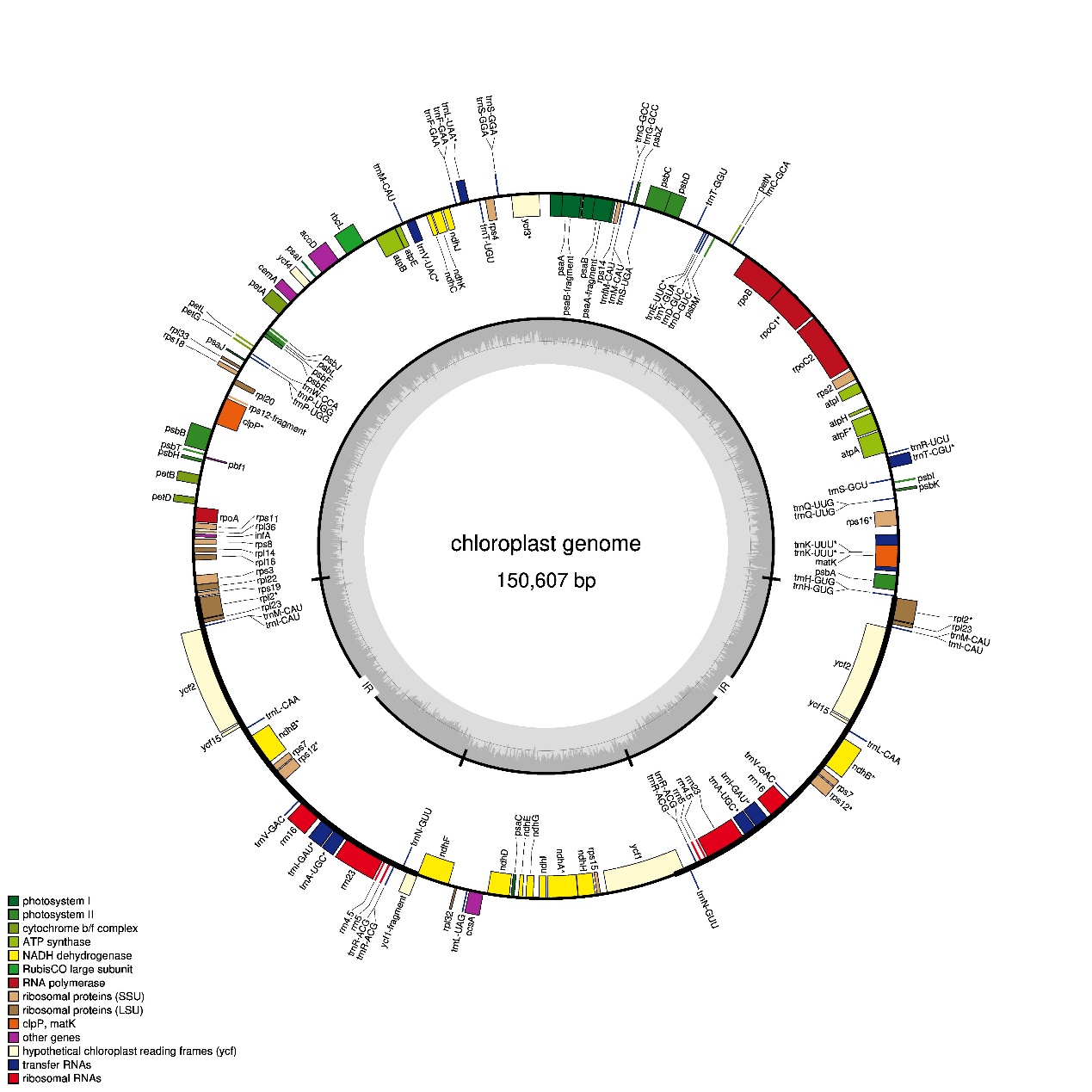


**Supplementary Figure 1. Gene map of *S. splendens* chloroplast genome.** The genes inside and outside of the circle are transcribed in the clockwise and counterclockwise directions, respectively. Genes belonging to different functional groups are shown in different colors. The thick lines indicate the extent of the inverted repeats (IRa and IRb) that separate the genomes into small single-copy and large single-copy regions.


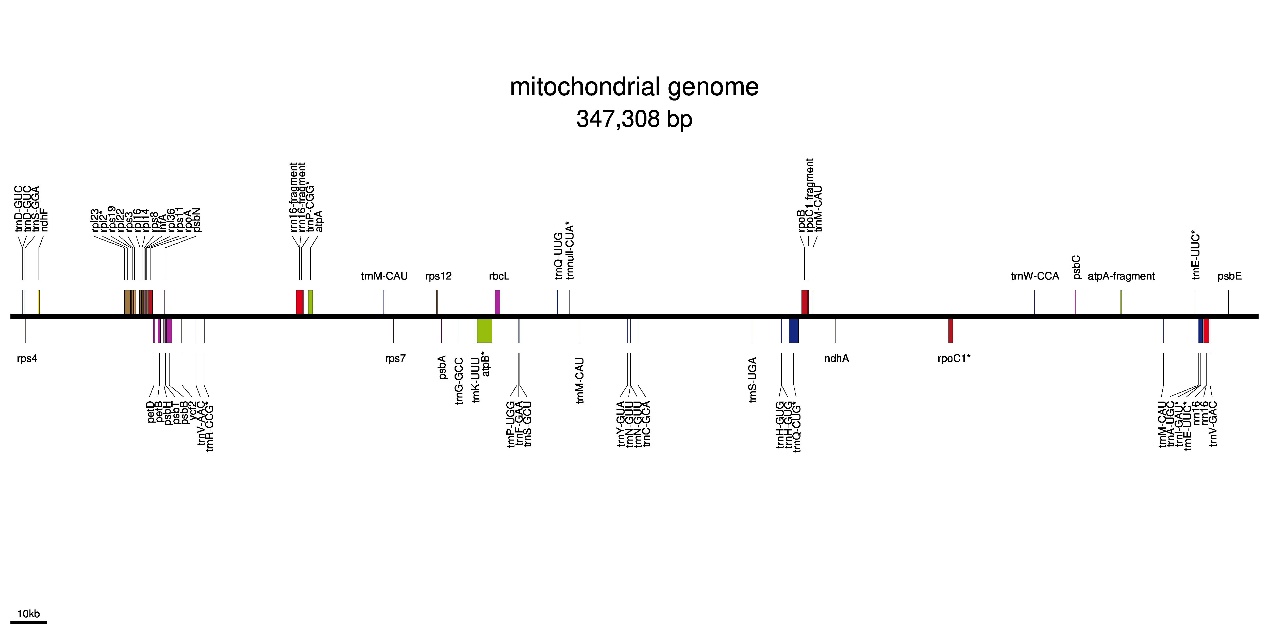


**Supplementary Figure 2. Gene map of *S. splendens* mitochondrial genome.** Genes belonging to different functional groups are shown in different colors.


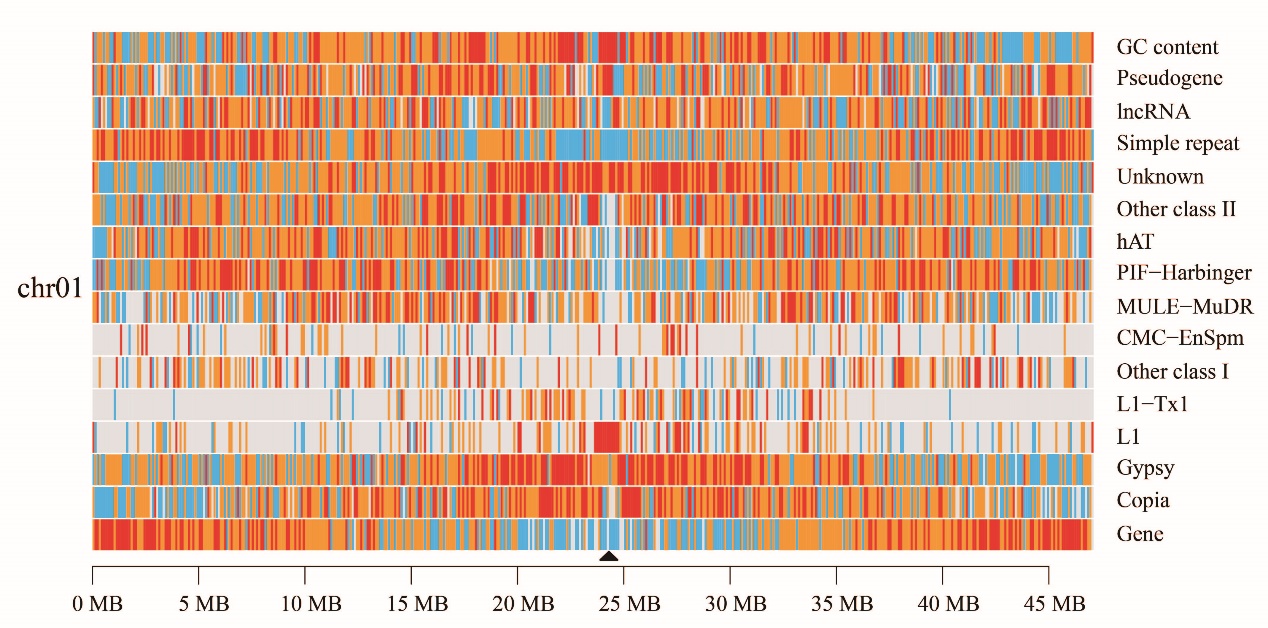


**Supplementary Figure 3. Distribution of different genomic features along chromosome 01 in *S. splendens* genome.** X axis, The length (Mb) of chromosomes and the position of the pericentromere (black triangles); Y axis, different genomic features. Red: density is greater than 75%; Orange: density is between 25% and 75%; Blue: density is between 0% and 25%; Gray: density is 0%.


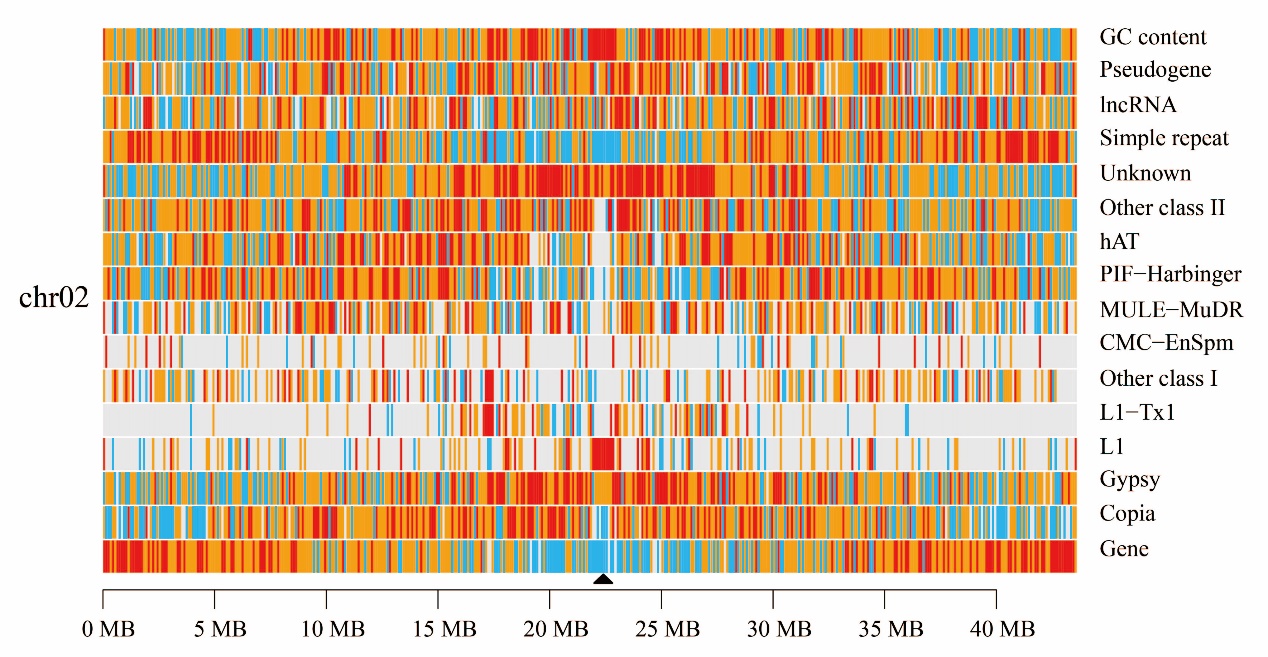


**Supplementary Figure 4. Distribution of different genomic features along chromosome 02 in *S. splendens* genome.** X axis, The length (Mb) of chromosomes and the position of the pericentromere (black triangles); Y axis, different genomic features. Red: density is greater than 75%; Orange: density is between 25% and 75%; Blue: density is between 0% and 25%; Gray: density is 0%.


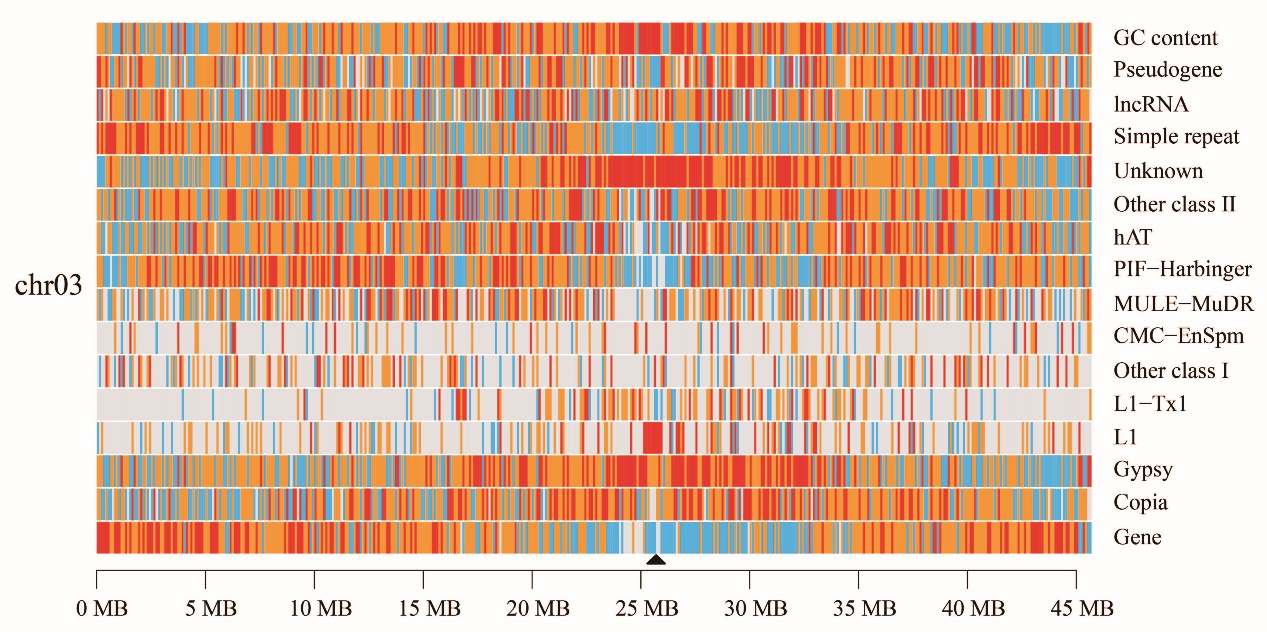


**Supplementary Figure 5. Distribution of different genomic features along chromosome 03 in *S. splendens* genome.** X axis, The length (Mb) of chromosomes and the position of the pericentromere (black triangles); Y axis, different genomic features. Red: density is greater than 75%; Orange: density is between 25% and 75%; Blue: density is between 0% and 25%; Gray: density is 0%.


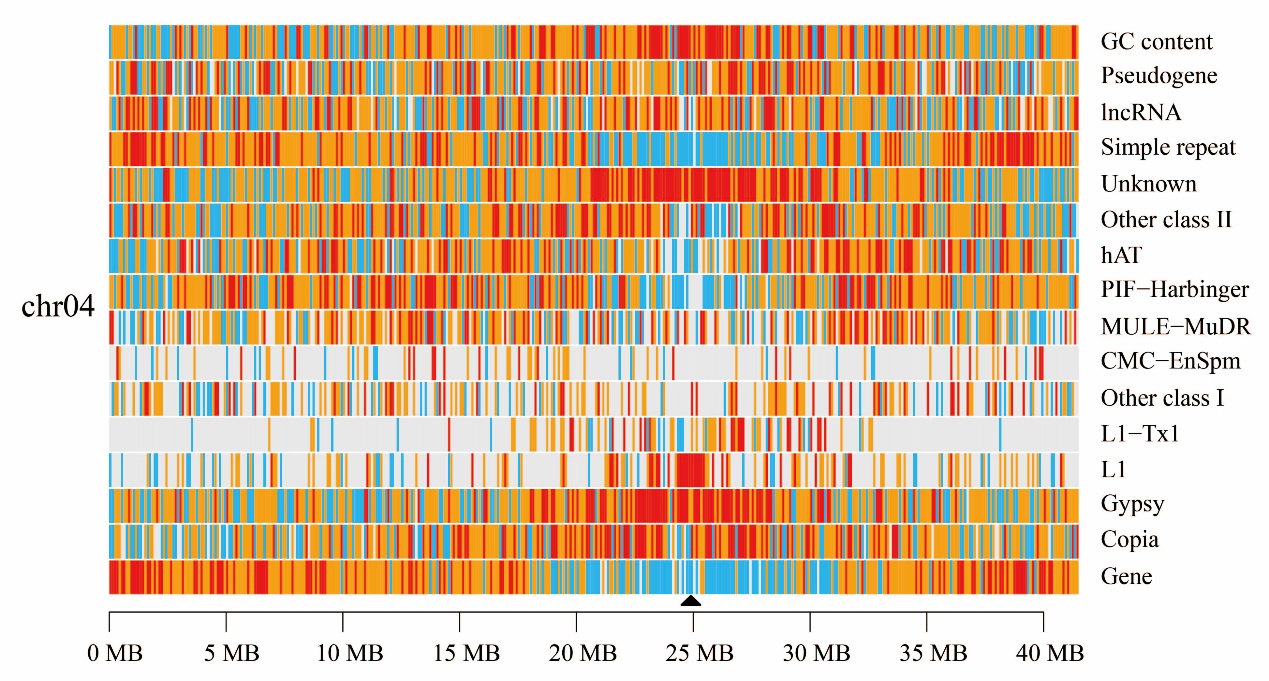


**Supplementary Figure 6. Distribution of different genomic features along chromosome 04 in *S. splendens* genome.** X axis, The length (Mb) of chromosomes and the position of the pericentromere (black triangles); Y axis, different genomic features. Red: density is greater than 75%; Orange: density is between 25% and 75%; Blue: density is between 0% and 25%; Gray: density is 0%.


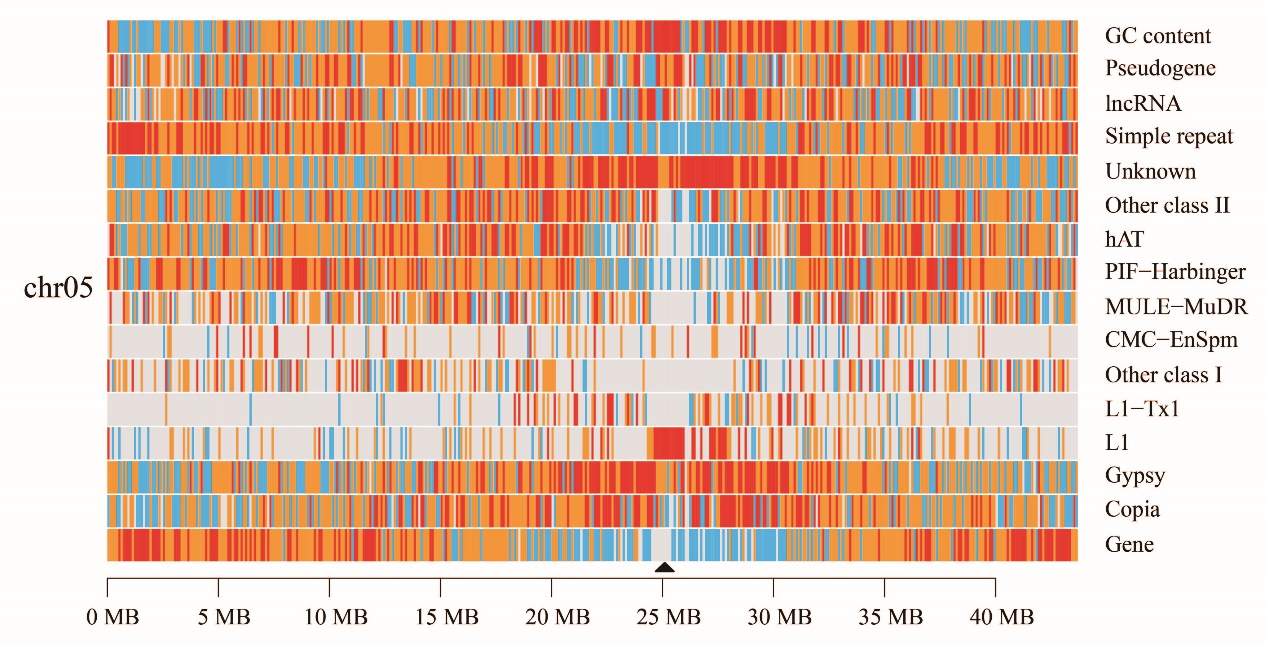


**Supplementary Figure 7. Distribution of different genomic features along chromosome 05 in *S. splendens* genome.** X axis, The length (Mb) of chromosomes and the position of the pericentromere (black triangles); Y axis, different genomic features. Red: density is greater than 75%; Orange: density is between 25% and 75%; Blue: density is between 0% and 25%; Gray: density is 0%.


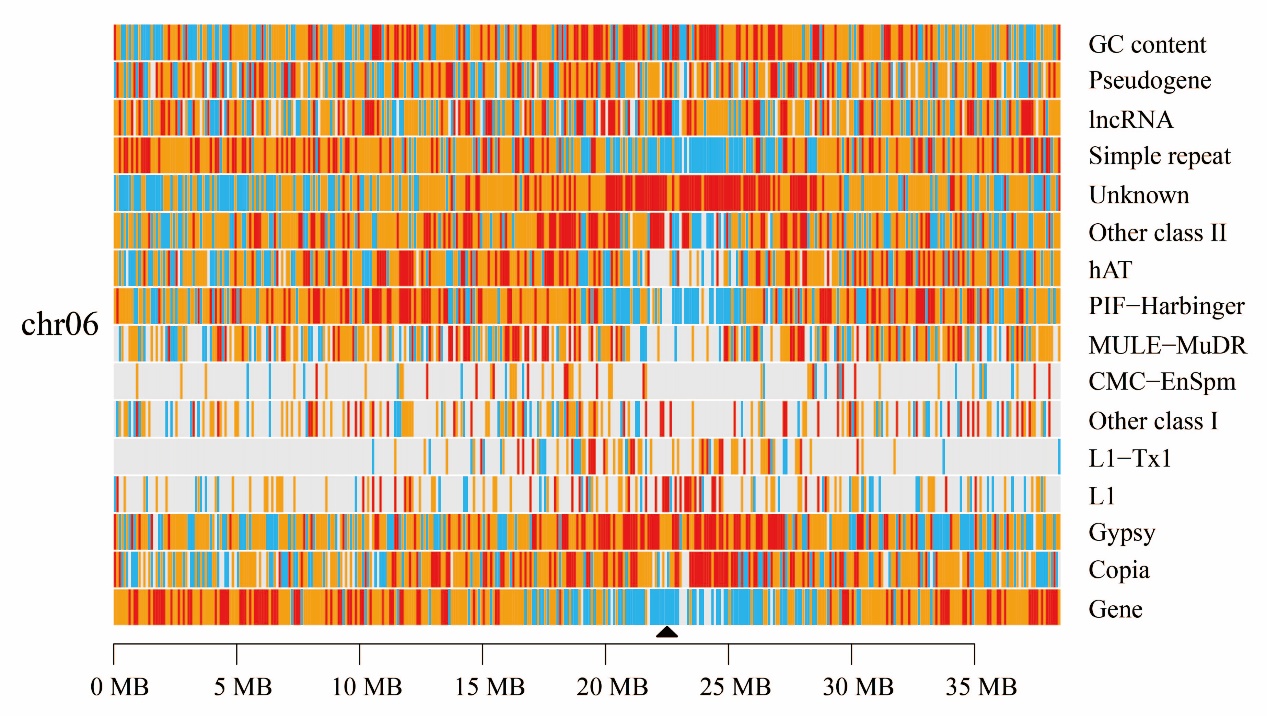


**Supplementary Figure 8. Distribution of different genomic features along chromosome 06 in *S. splendens* genome.** X axis, The length (Mb) of chromosomes and the position of the pericentromere (black triangles); Y axis, different genomic features. Red: density is greater than 75%; Orange: density is between 25% and 75%; Blue: density is between 0% and 25%; Gray: density is 0%.


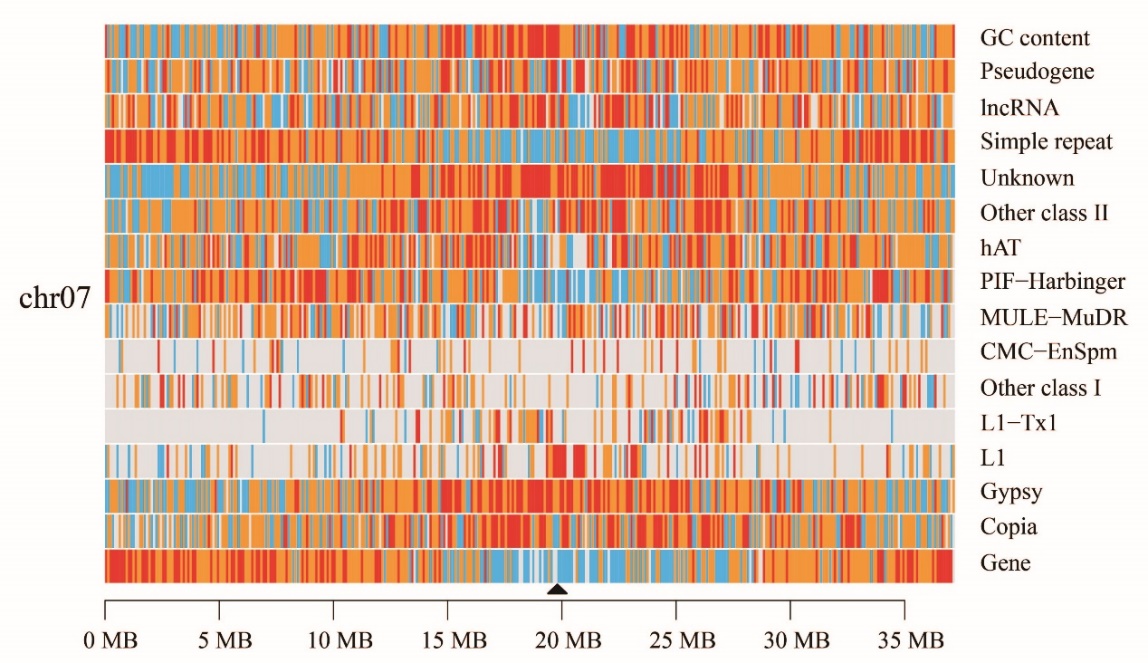


**Supplementary Figure 9. Distribution of different genomic features along chromosome 07 in *S. splendens* genome.** X axis, The length (Mb) of chromosomes and the position of the pericentromere (black triangles); Y axis, different genomic features. Red: density is greater than 75%; Orange: density is between 25% and 75%; Blue: density is between 0% and 25%; Gray: density is 0%.


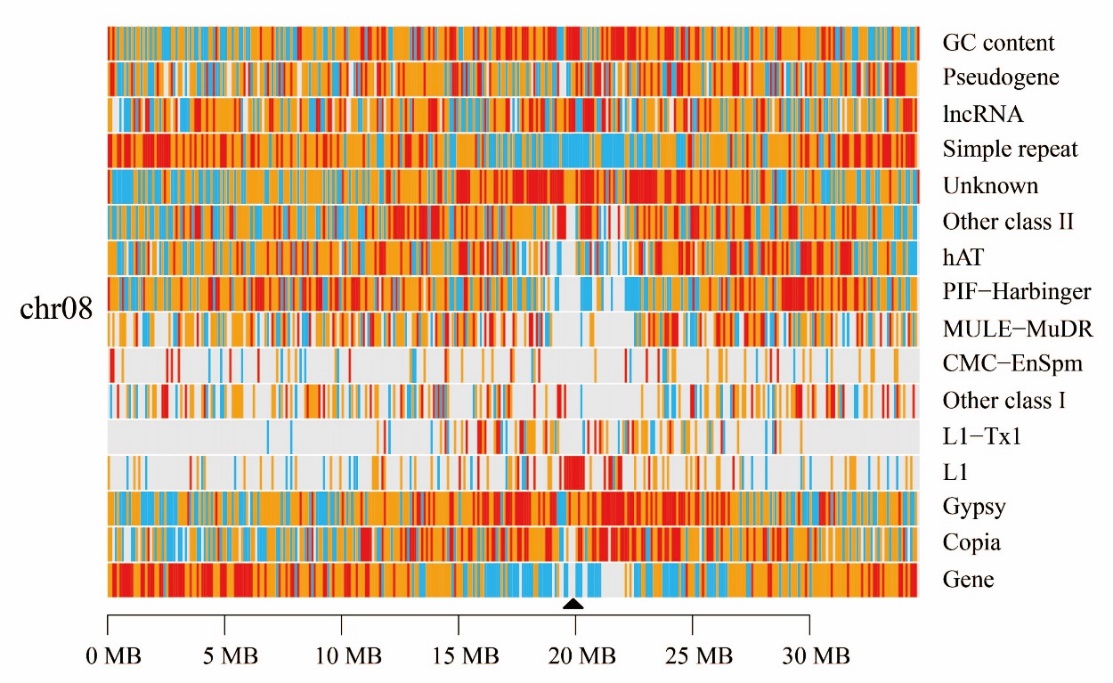


**Supplementary Figure 10. Distribution of different genomic features along chromosome 08 in *S. splendens* genome.** X axis, The length (Mb) of chromosomes and the position of the pericentromere (black triangles); Y axis, different genomic features. Red: density is greater than 75%; Orange: density is between 25% and 75%; Blue: density is between 0% and 25%; Gray: density is 0%.


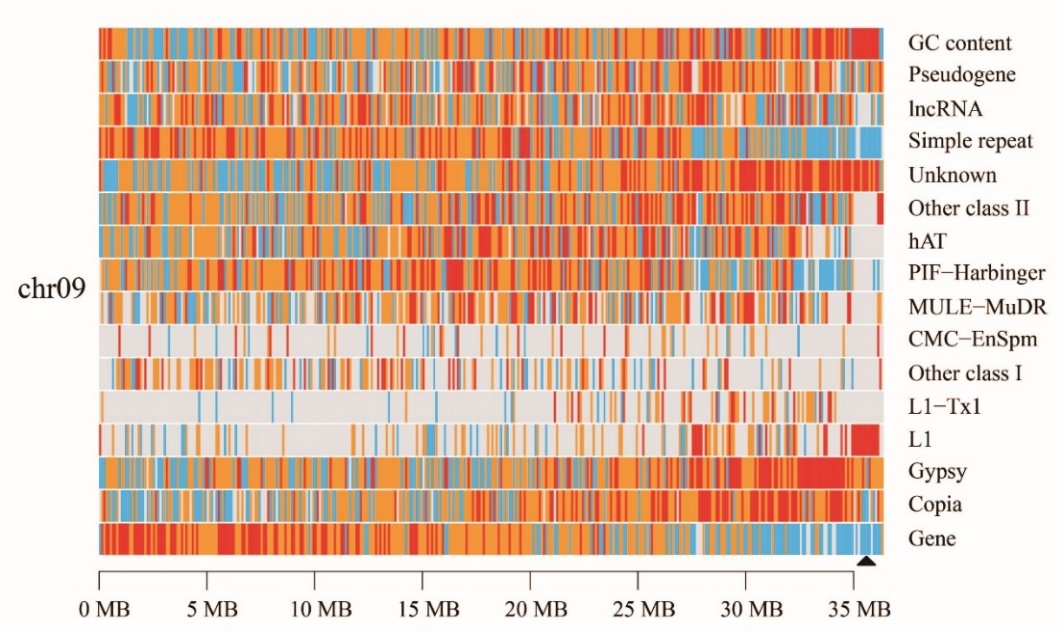


**Supplementary Figure 11. Distribution of different genomic features along chromosome 09 in *S. splendens* genome.** X axis, The length (Mb) of chromosomes and the position of the pericentromere (black triangles); Y axis, different genomic features. Red: density is greater than 75%; Orange: density is between 25% and 75%; Blue: density is between 0% and 25%; Gray: density is 0%.


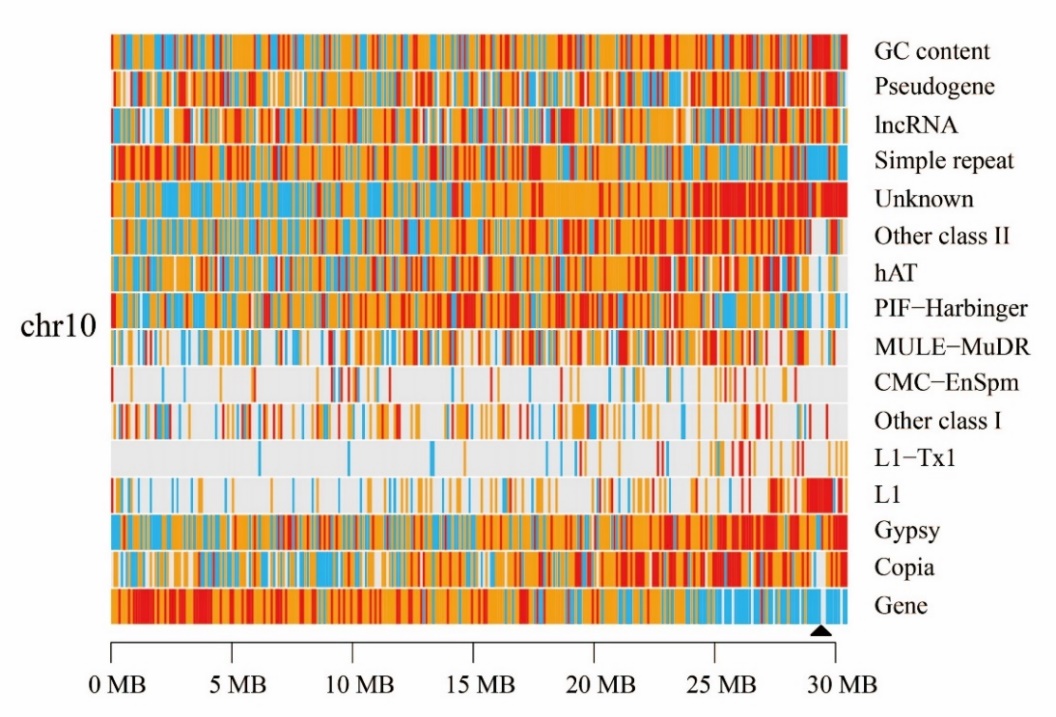


**Supplementary Figure 12. Distribution of different genomic features along chromosome 10 in *S. splendens* genome.** X axis, The length (Mb) of chromosomes and the position of the pericentromere (black triangles); Y axis, different genomic features. Red: density is greater than 75%; Orange: density is between 25% and 75%; Blue: density is between 0% and 25%; Gray: density is 0%.


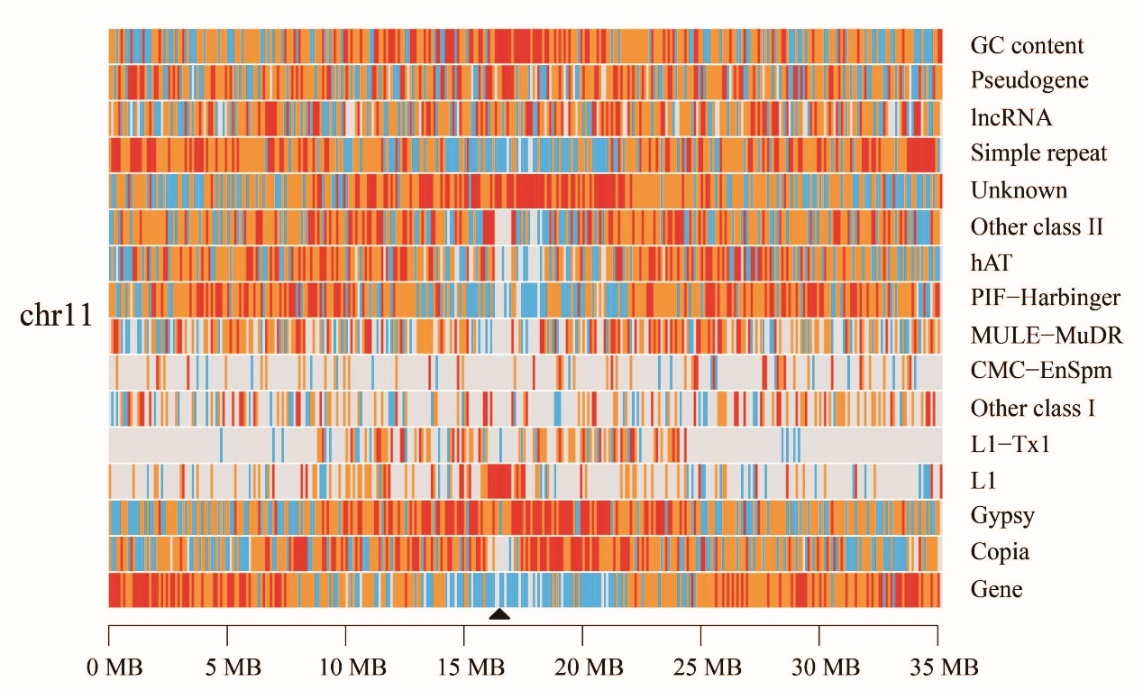


**Supplementary Figure 13. Distribution of different genomic features along chromosome 11 in *S. splendens* genome.** X axis, The length (Mb) of chromosomes and the position of the pericentromere (black triangles); Y axis, different genomic features. Red: density is greater than 75%; Orange: density is between 25% and 75%; Blue: density is between 0% and 25%; Gray: density is 0%.


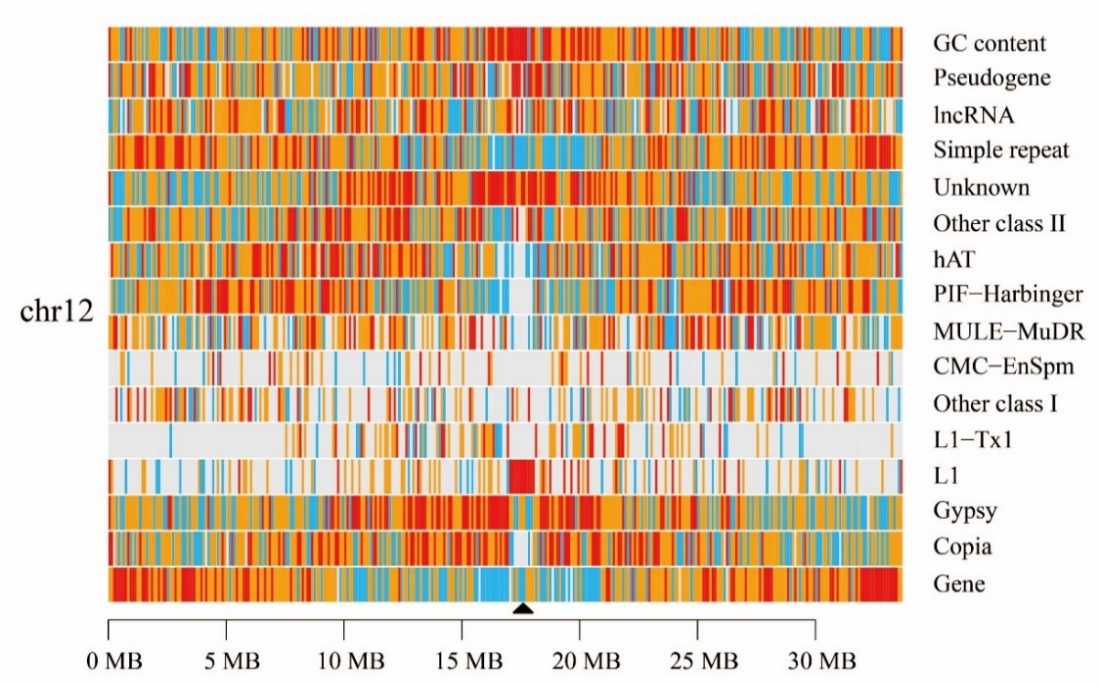


**Supplementary Figure 14. Distribution of different genomic features along chromosome 12 in *S. splendens* genome.** X axis, The length (Mb) of chromosomes and the position of the pericentromere (black triangles); Y axis, different genomic features. Red: density is greater than 75%; Orange: density is between 25% and 75%; Blue: density is between 0% and 25%; Gray: density is 0%.


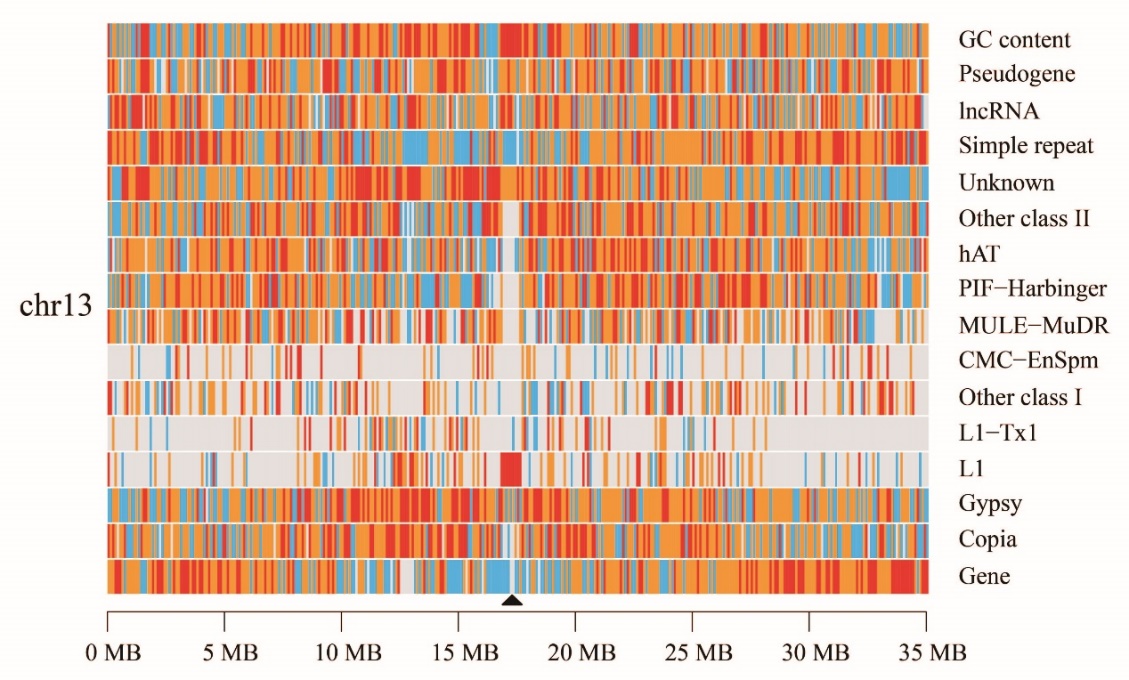


**Supplementary Figure 15. Distribution of different genomic features along chromosome 13 in *S. splendens* genome.** X axis, The length (Mb) of chromosomes and the position of the pericentromere (black triangles); Y axis, different genomic features. Red: density is greater than 75%; Orange: density is between 25% and 75%; Blue: density is between 0% and 25%; Gray: density is 0%.


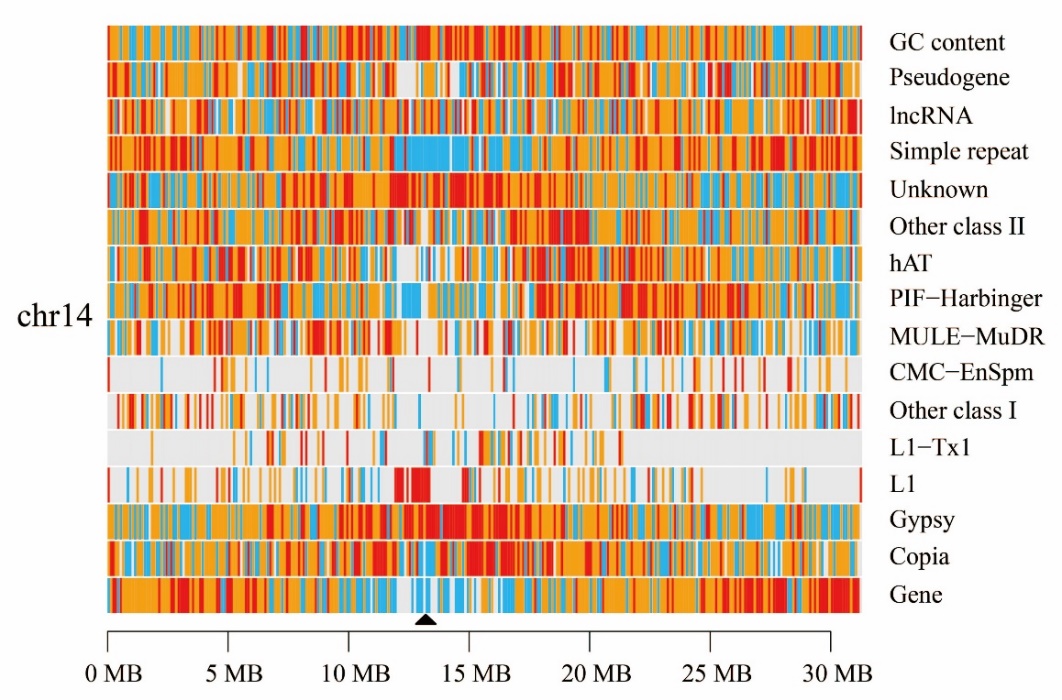


**Supplementary Figure 16. Distribution of different genomic features along chromosome 14 in *S. splendens* genome.** X axis, The length (Mb) of chromosomes and the position of the pericentromere (black triangles); Y axis, different genomic features. Red: density is greater than 75%; Orange: density is between 25% and 75%; Blue: density is between 0% and 25%; Gray: density is 0%.


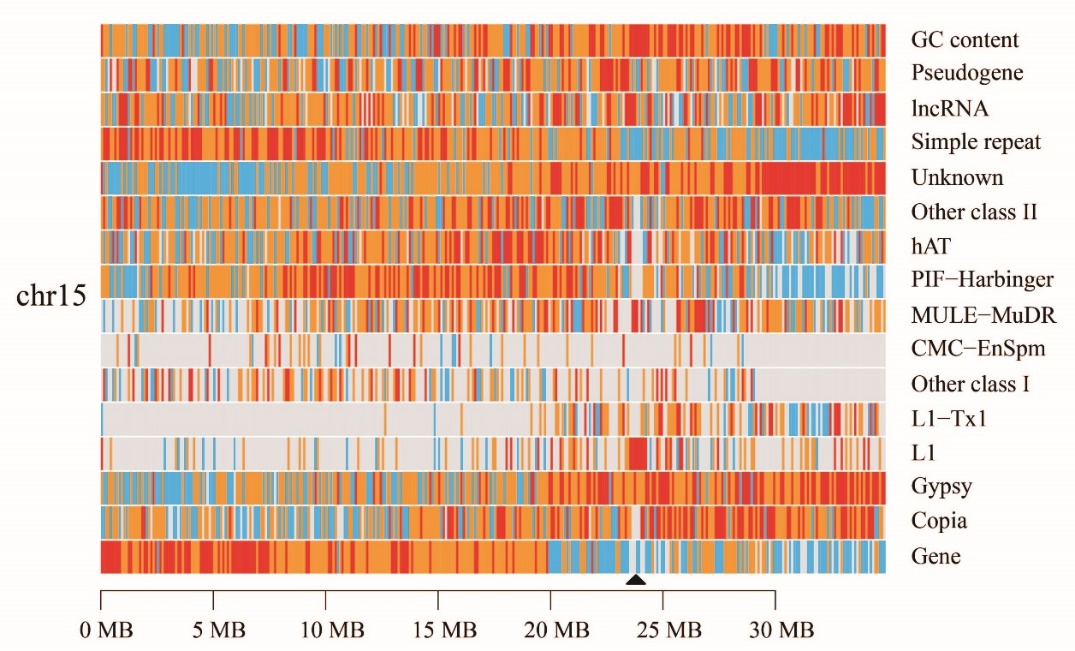


**Supplementary Figure 17. Distribution of different genomic features along chromosome 15 in *S. splendens* genome.** X axis, The length (Mb) of chromosomes and the position of the pericentromere (black triangles); Y axis, different genomic features. Red: density is greater than 75%; Orange: density is between 25% and 75%; Blue: density is between 0% and 25%; Gray: density is 0%.


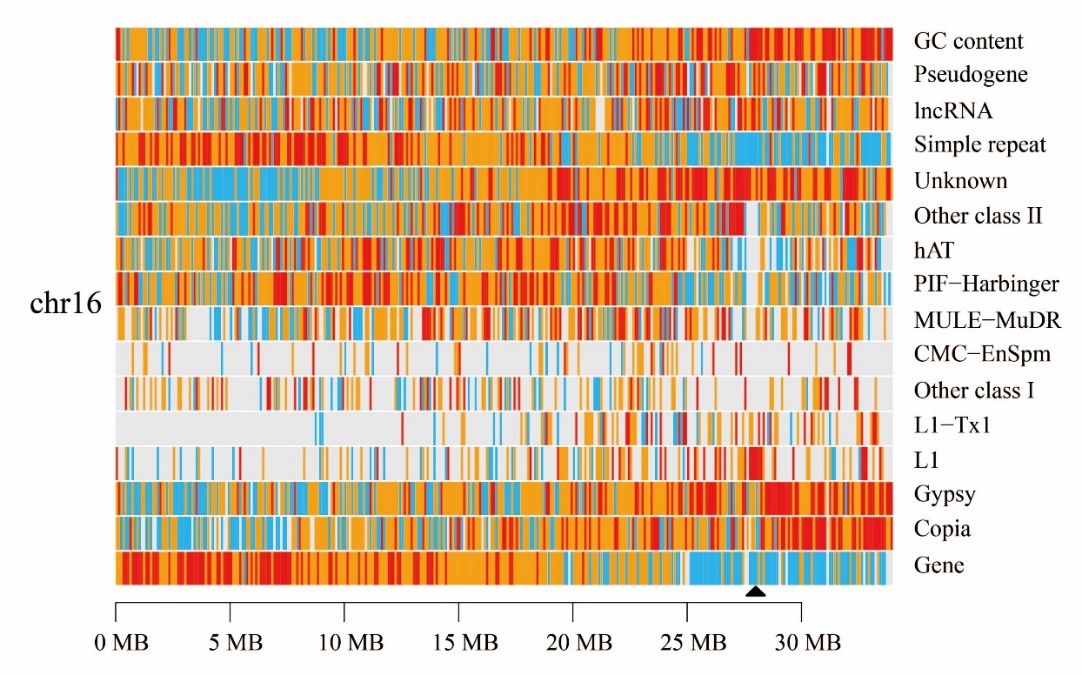


**Supplementary Figure 18. Distribution of different genomic features along chromosome 16 in *S. splendens* genome.** X axis, The length (Mb) of chromosomes and the position of the pericentromere (black triangles); Y axis, different genomic features. Red: density is greater than 75%; Orange: density is between 25% and 75%; Blue: density is between 0% and 25%; Gray: density is 0%.


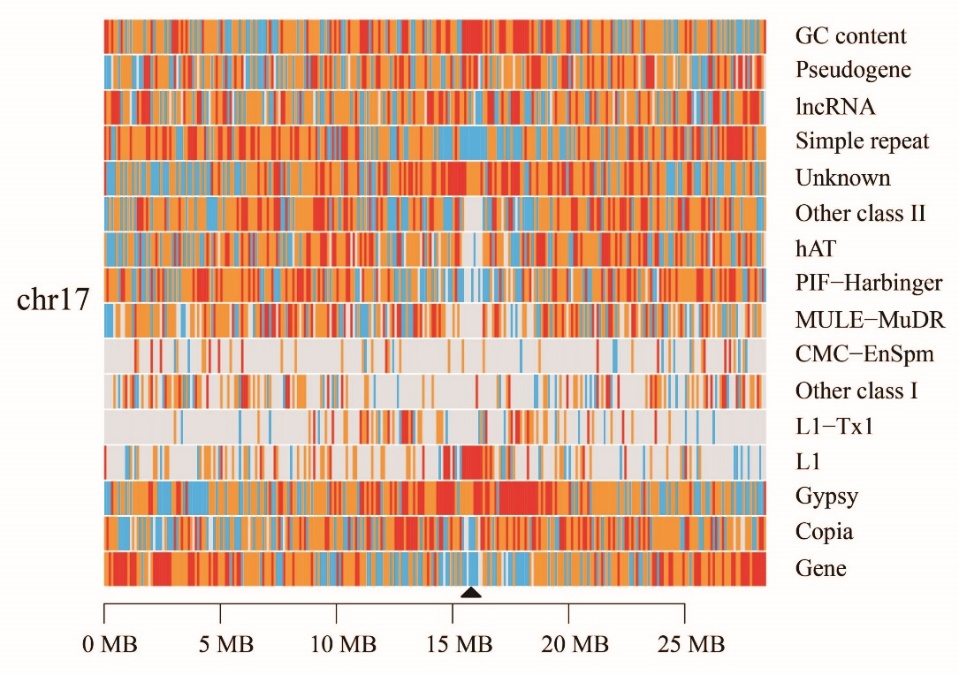


**Supplementary Figure 19. Distribution of different genomic features along chromosome 17 in *S. splendens* genome.** X axis, The length (Mb) of chromosomes and the position of the pericentromere (black triangles); Y axis, different genomic features. Red: density is greater than 75%; Orange: density is between 25% and 75%; Blue: density is between 0% and 25%; Gray: density is 0%.


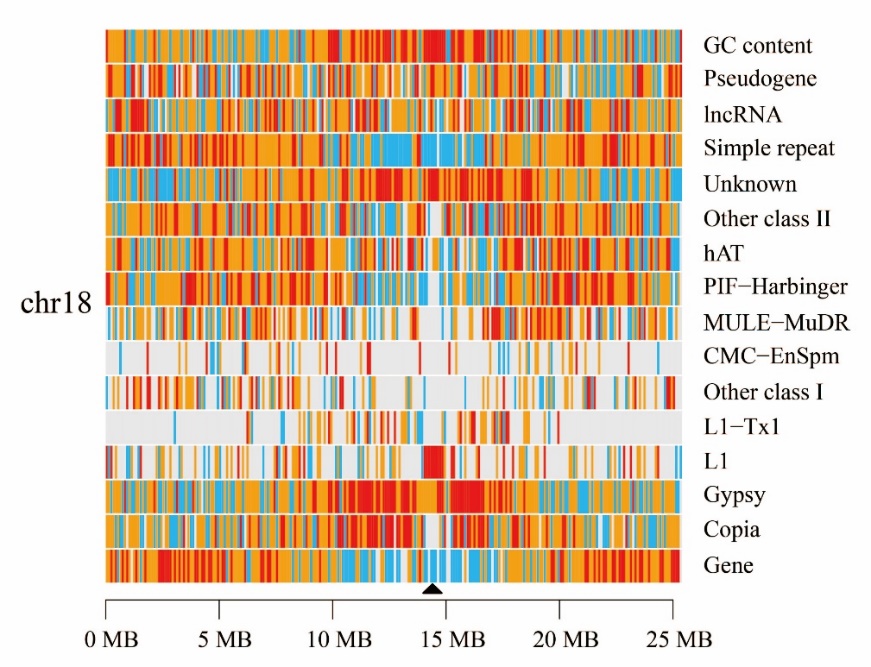


**Supplementary Figure 20. Distribution of different genomic features along chromosome 18 in *S. splendens* genome.** X axis, The length (Mb) of chromosomes and the position of the pericentromere (black triangles); Y axis, different genomic features. Red: density is greater than 75%; Orange: density is between 25% and 75%; Blue: density is between 0% and 25%; Gray: density is 0%.


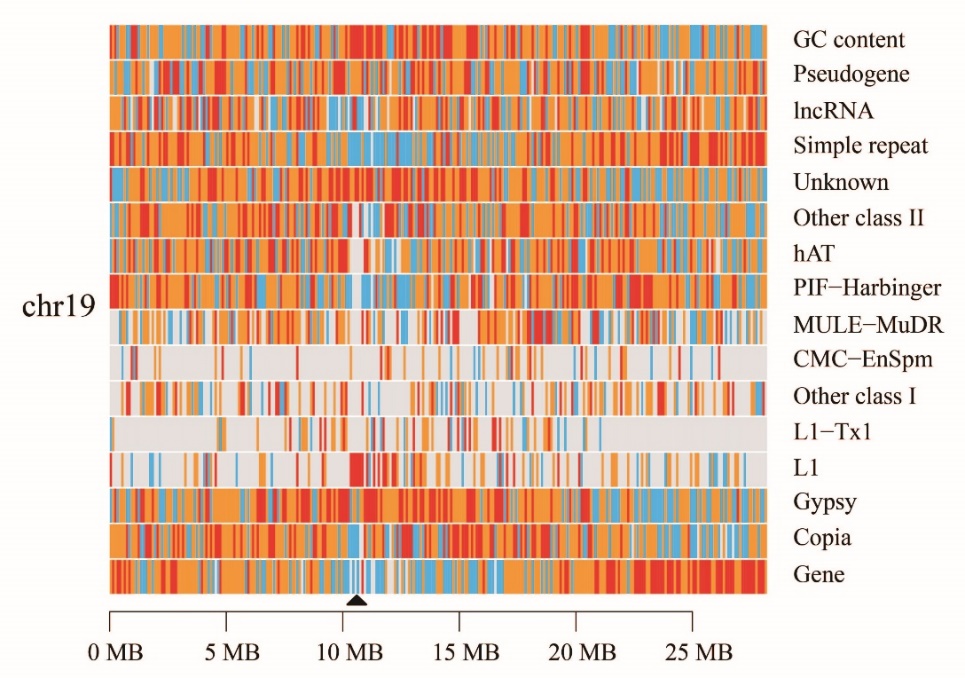


**Supplementary Figure 21. Distribution of different genomic features along chromosome 19 in *S. splendens* genome.** X axis, The length (Mb) of chromosomes and the position of the pericentromere (black triangles); Y axis, different genomic features. Red: density is greater than 75%; Orange: density is between 25% and 75%; Blue: density is between 0% and 25%; Gray: density is 0%.


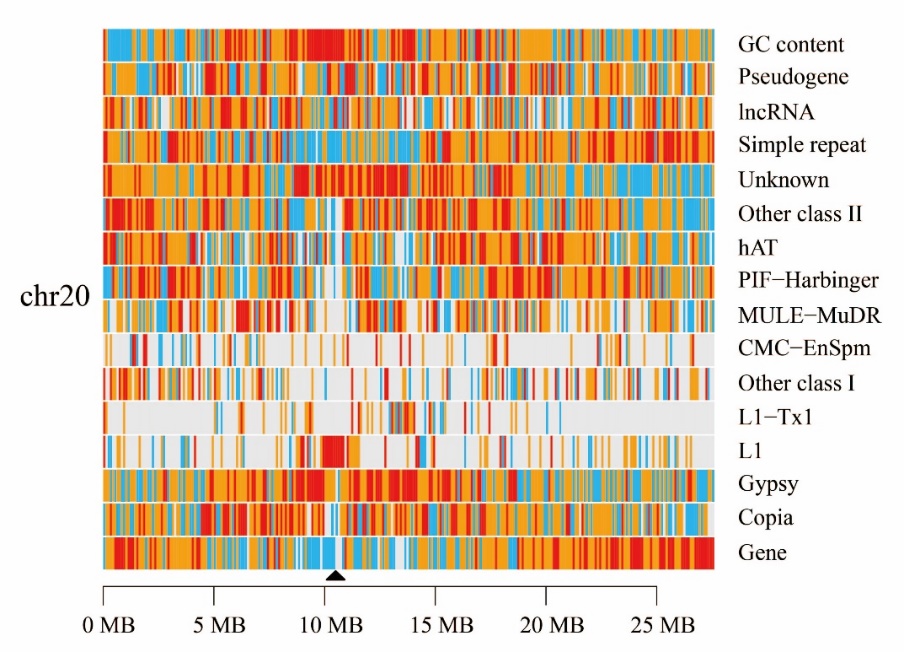


**Supplementary Figure 22. Distribution of different genomic features along chromosome 20 in *S. splendens* genome.** X axis, The length (Mb) of chromosomes and the position of the pericentromere (black triangles); Y axis, different genomic features. Red: density is greater than 75%; Orange: density is between 25% and 75%; Blue: density is between 0% and 25%; Gray: density is 0%.


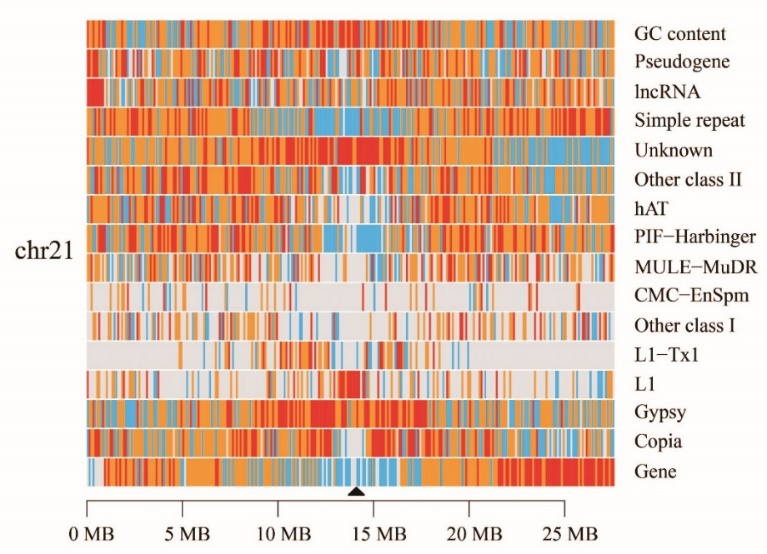


**Supplementary Figure 23. Distribution of different genomic features along chromosome 21 in *S. splendens* genome.** X axis, The length (Mb) of chromosomes and the position of the pericentromere (black triangles); Y axis, different genomic features. Red: density is greater than 75%; Orange: density is between 25% and 75%; Blue: density is between 0% and 25%; Gray: density is 0%.


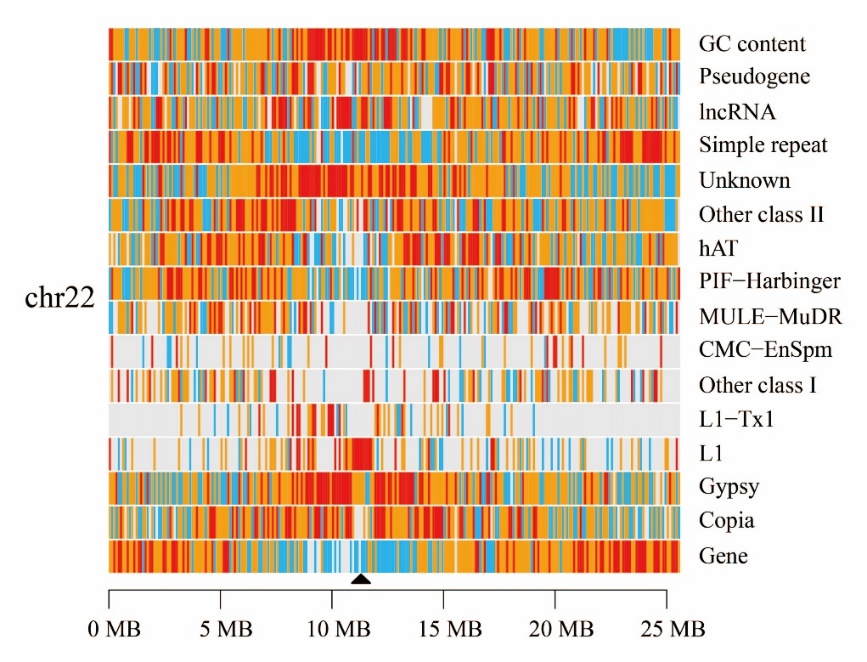


**Supplementary Figure 24. Distribution of different genomic features along chromosome 22 in *S. splendens* genome.** X axis, The length (Mb) of chromosomes and the position of the pericentromere (black triangles); Y axis, different genomic features. Red: density is greater than 75%; Orange: density is between 25% and 75%; Blue: density is between 0% and 25%; Gray: density is 0%.


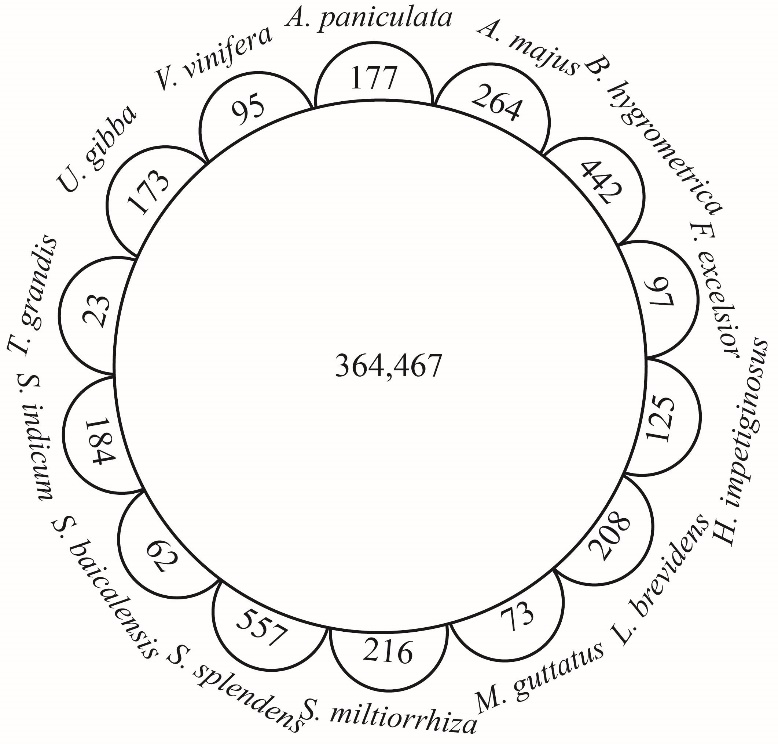


**Supplementary Figure 25. The number of orthologous genes sharing/unique among different species.** Large circle indicates all orthologous genes in different species, while small circles indicate genes unique to each species.


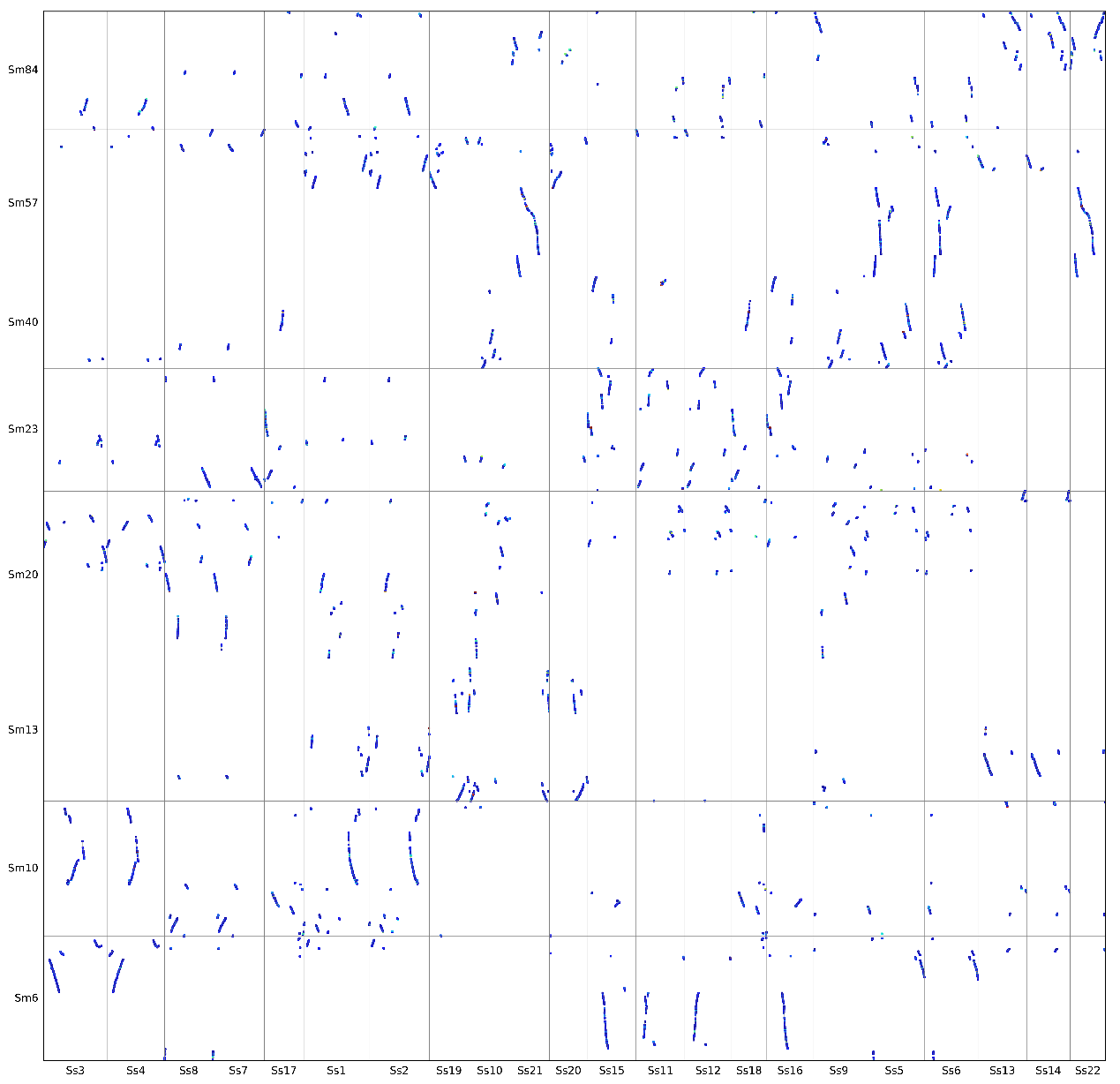


**Supplementary Figure 26. The dot plot comparing *S. splendens* and *S. miltiorrhiza*.** Ss, *S. splendens*; Sm, *S. miltiorrhiza*.


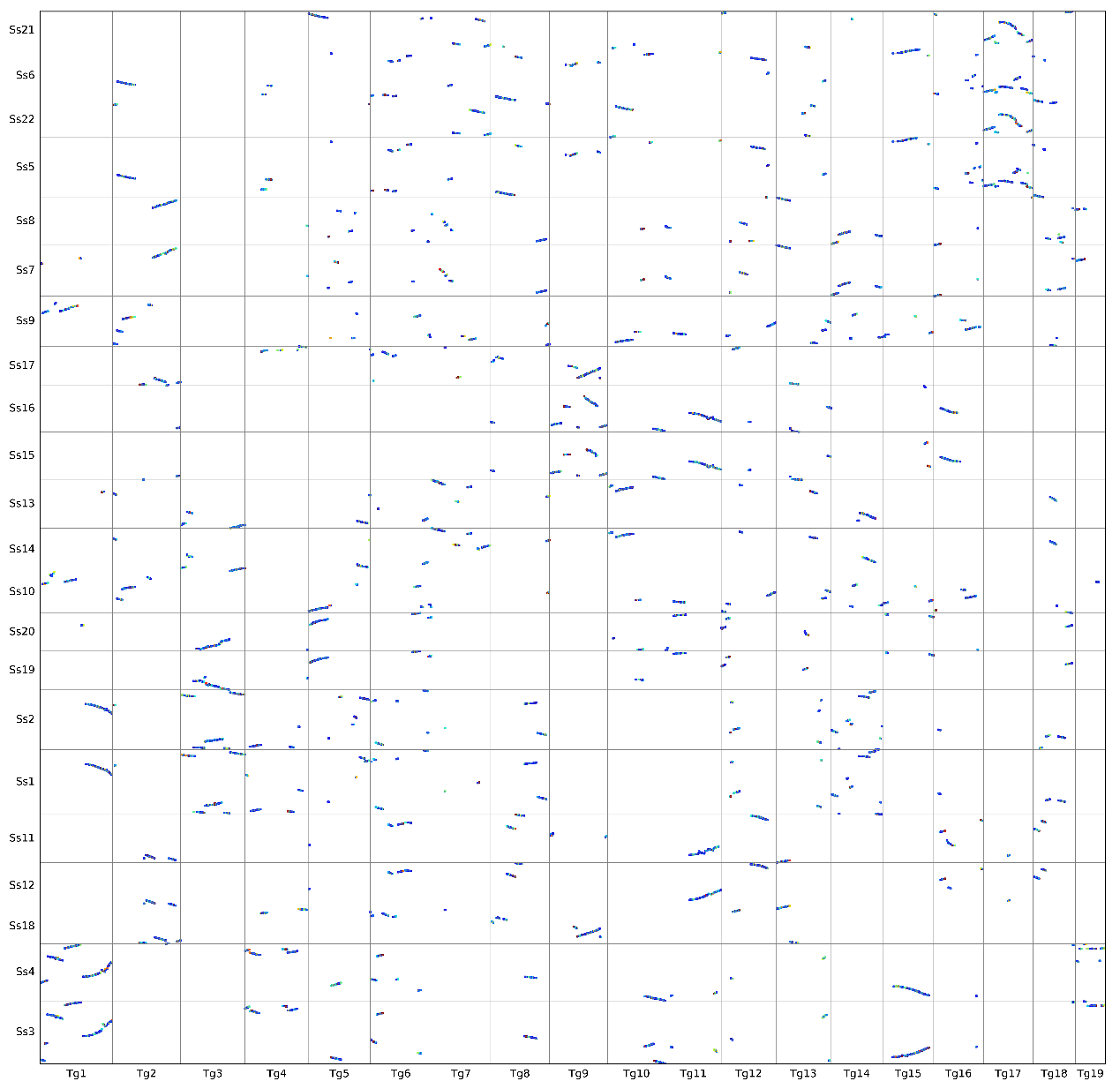


**Supplementary Figure 27. The dot plot comparing *T. grandis* and *S. splendens*.** Ss, *S. splendens*; Tg, *T. grandis*.


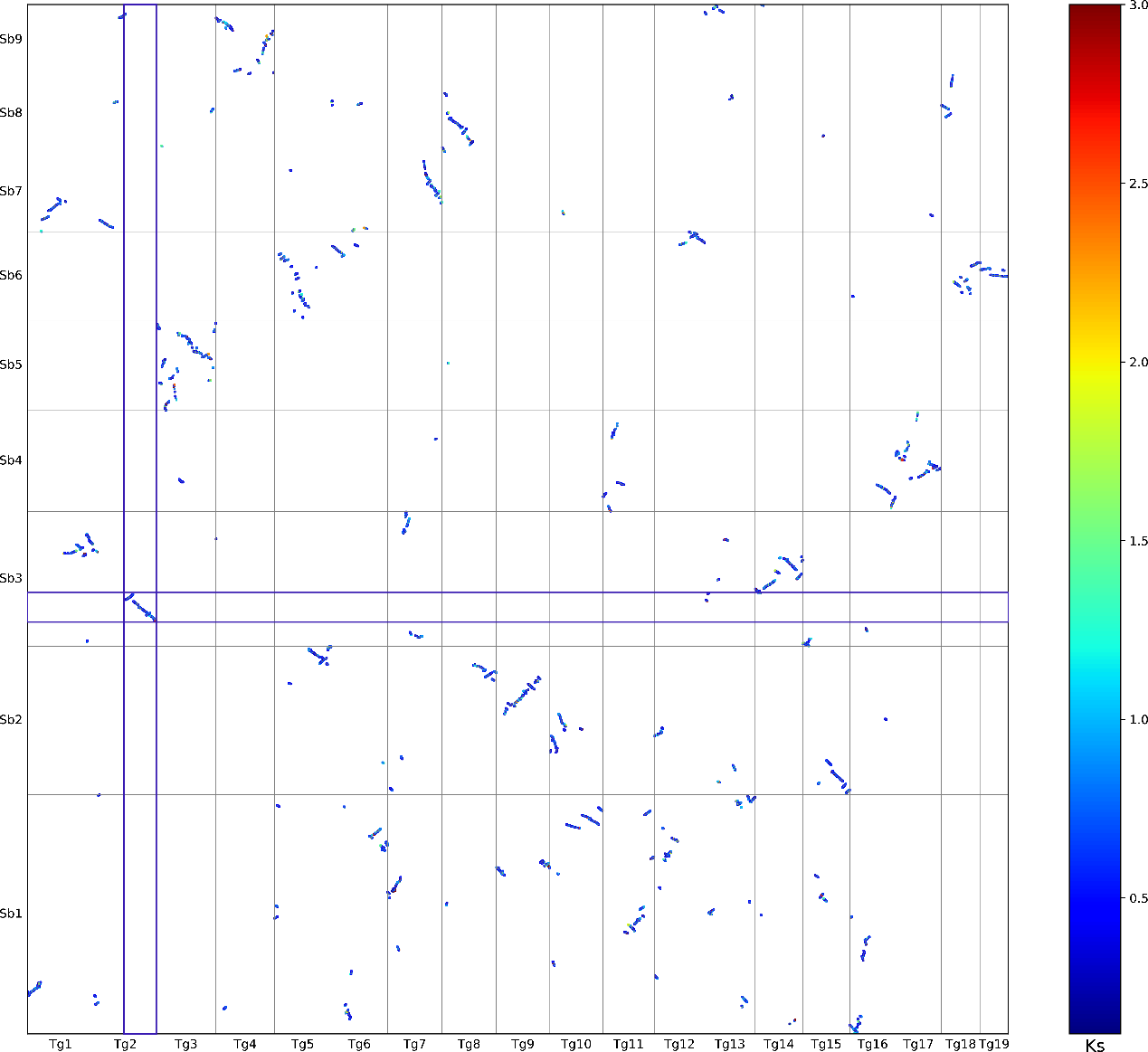


**Supplementary Figure 28. The dot plot comparing *T. grandis* and *S. baicalensis*.** Sb, *S. baicalensis*; Tg, *T. grandis*.


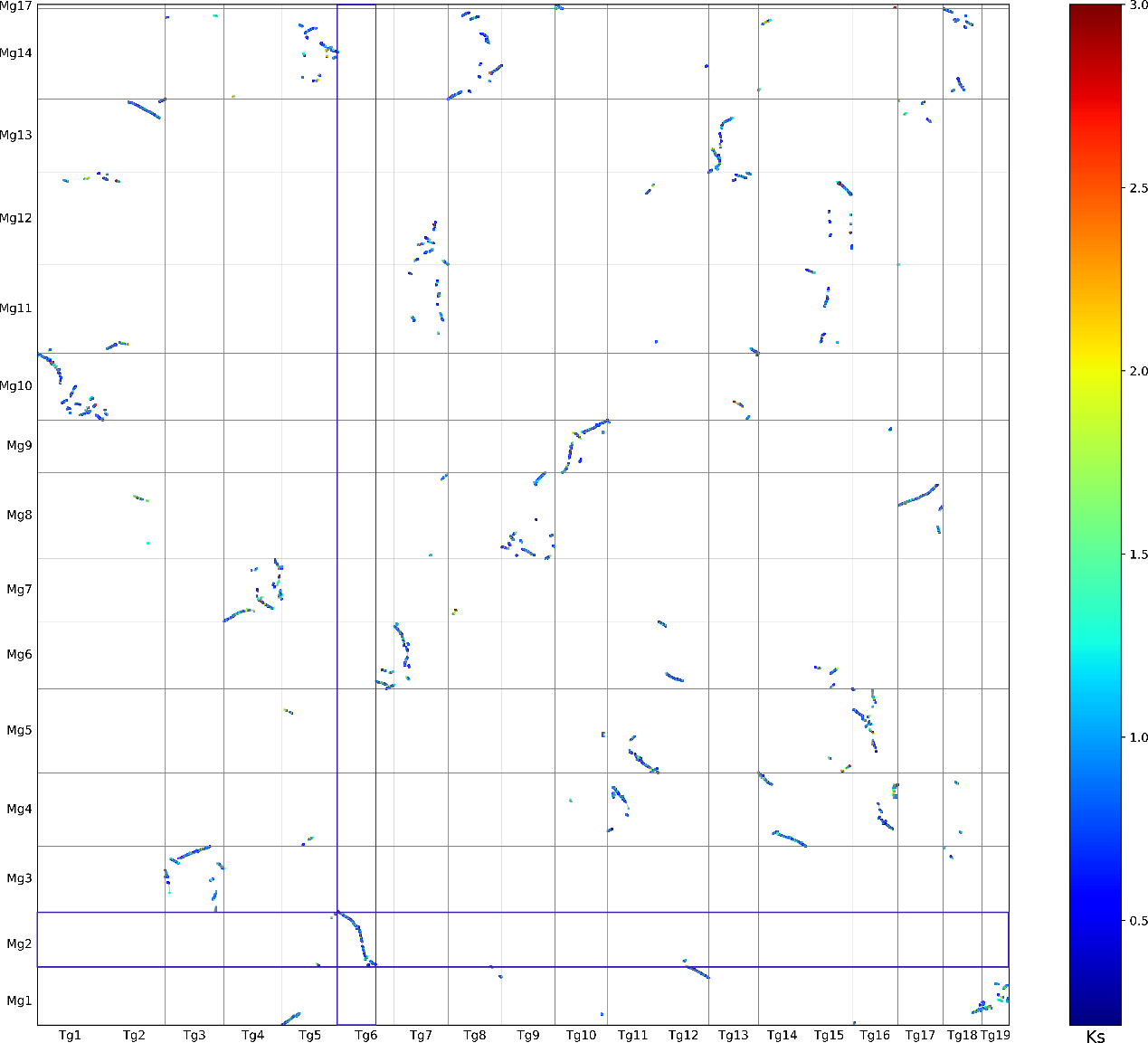


**Supplementary Figure 29.** **The dot plot comparing *T. grandis* and *M. guttatus*.** Mg, *M. guttatus*; Tg, *T. grandis*.


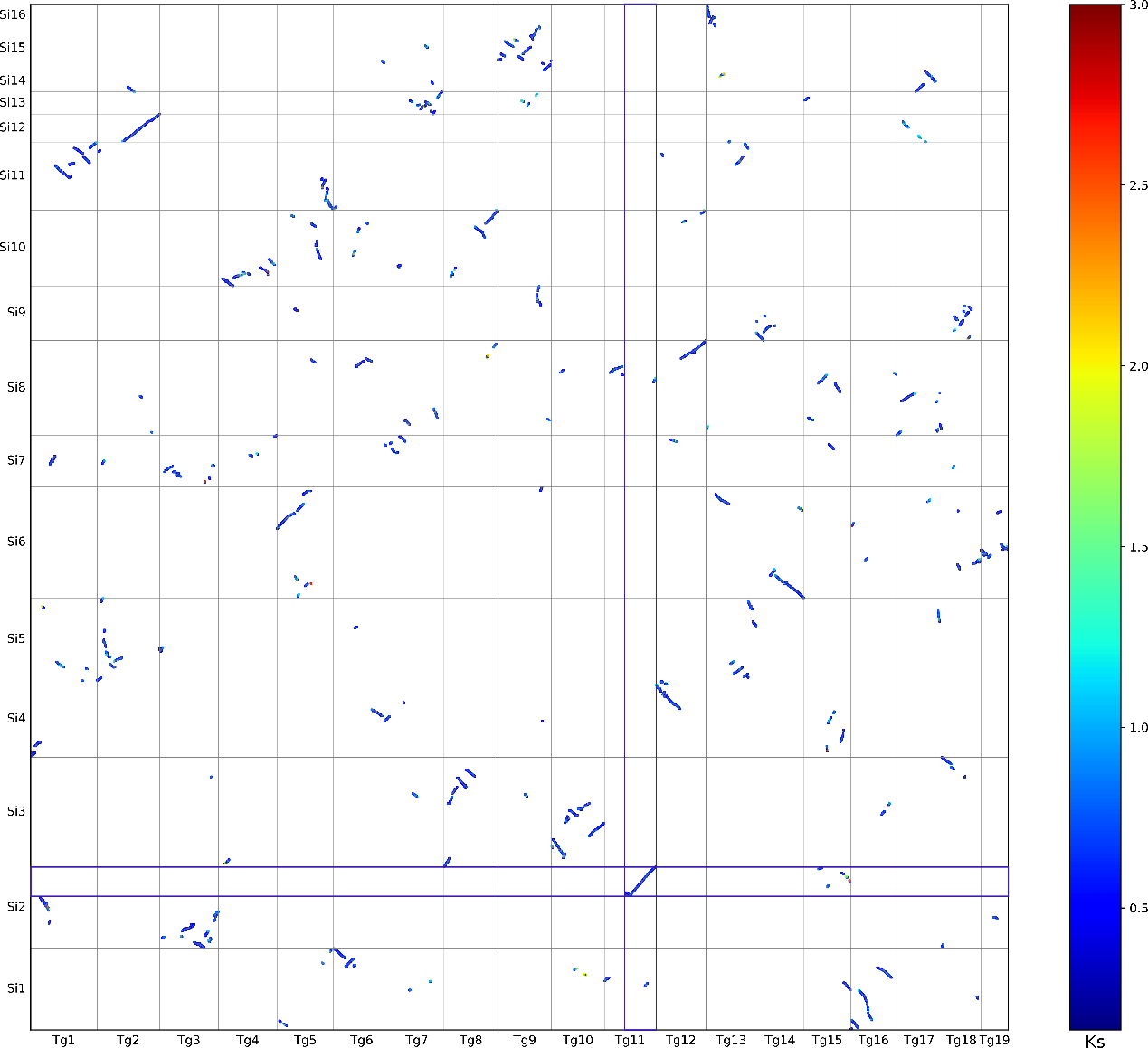


**Supplementary Figure 30. The dot plot comparing *T. grandis* and *S. indicum*.** Si, *S. indicum*, Tg, *T. grandis*.


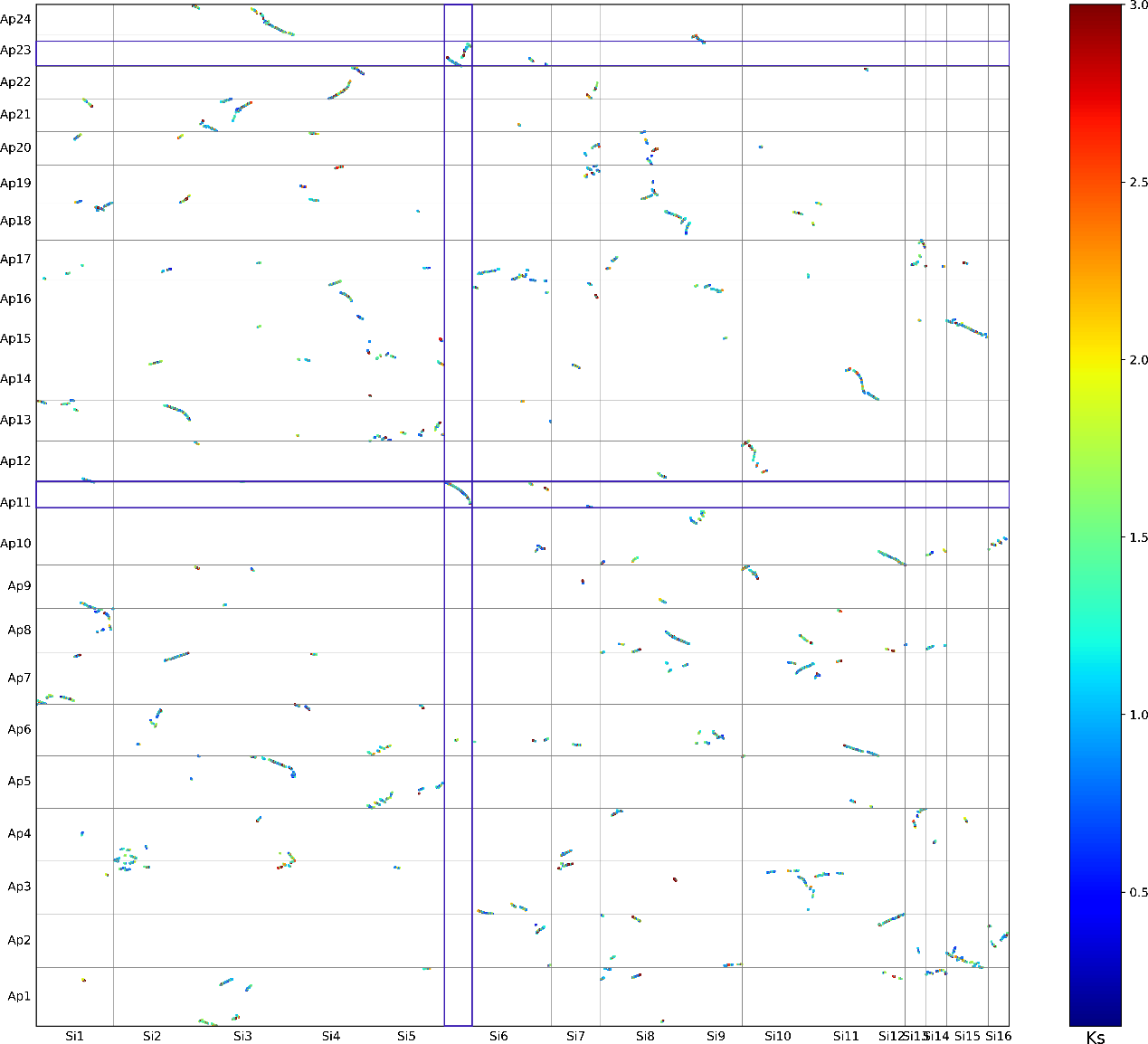


**Supplementary Figure 31. The dot plot comparing** ***A. paniculata* and *S. indicum*.** Ap, *A. paniculate*; Si, *S. indicum*.


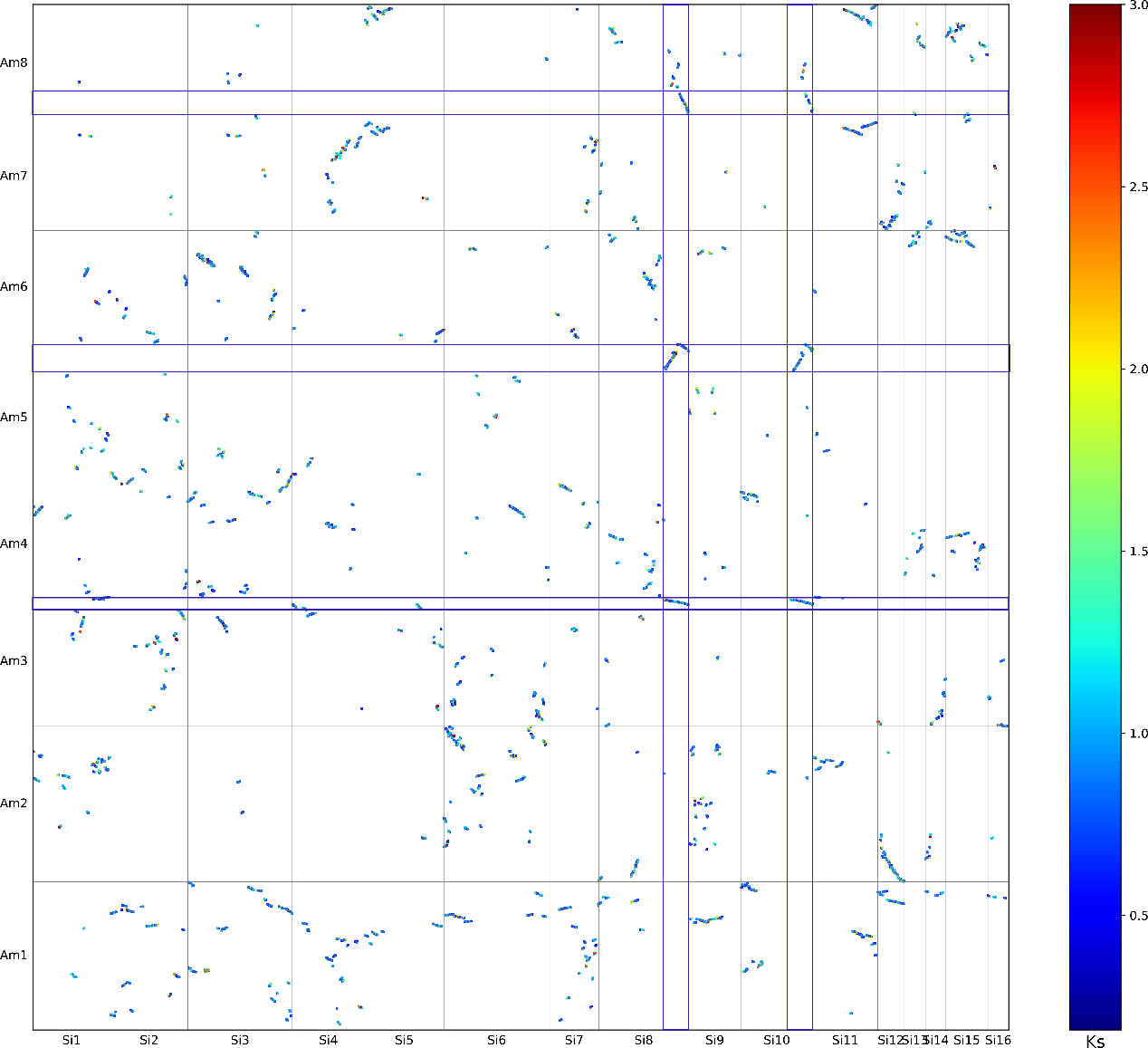


**Supplementary Figure 32. The dot plot comparing *A. majus* and *S. indicum*.** Am, *A. majus*; Si, *S. indicum*.


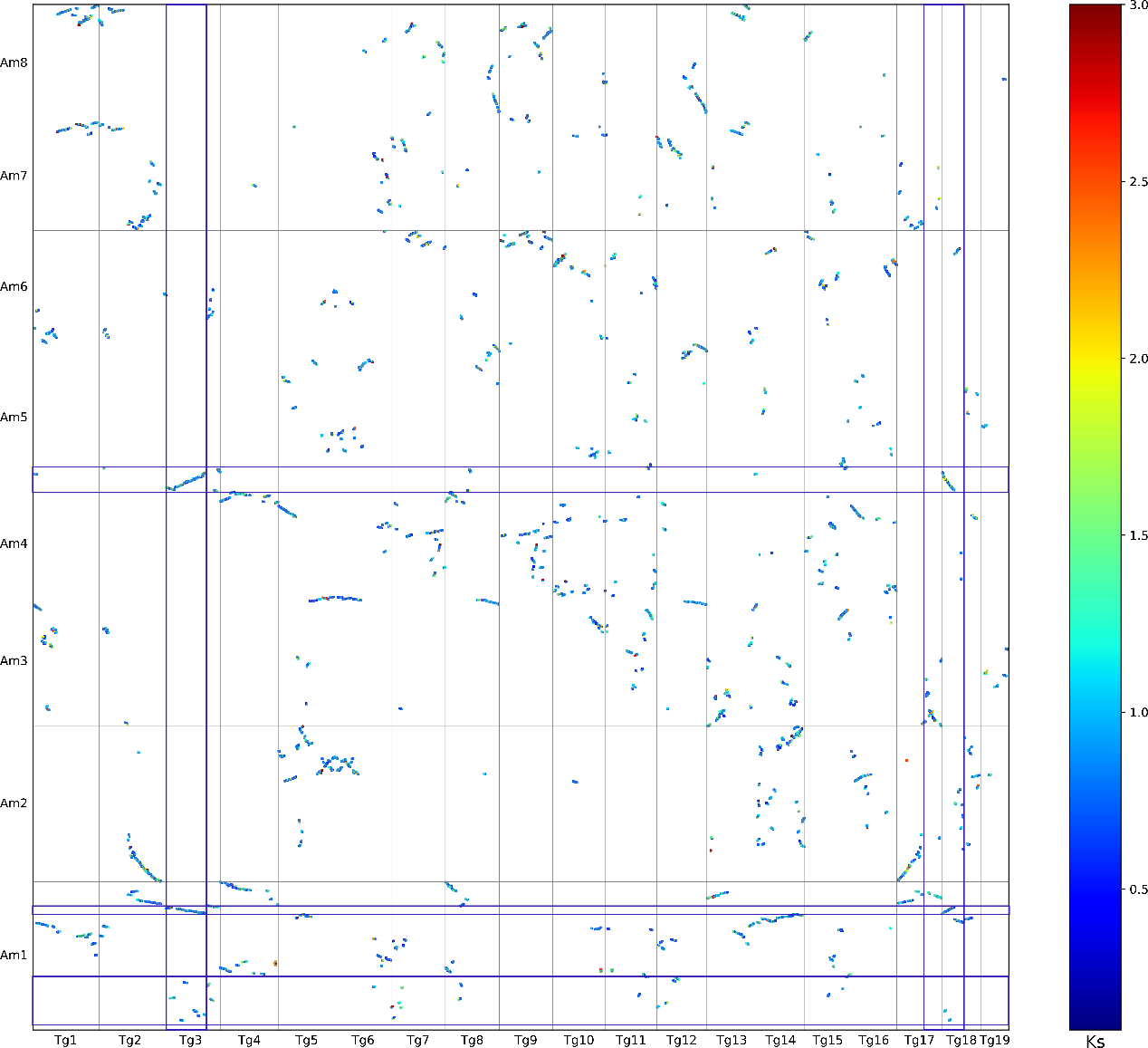


**Supplementary Figure 33. The dot plot comparing *A. majus* and** ***T. grandis*.** Am, *A. majus*; Tg, *T. grandis*.


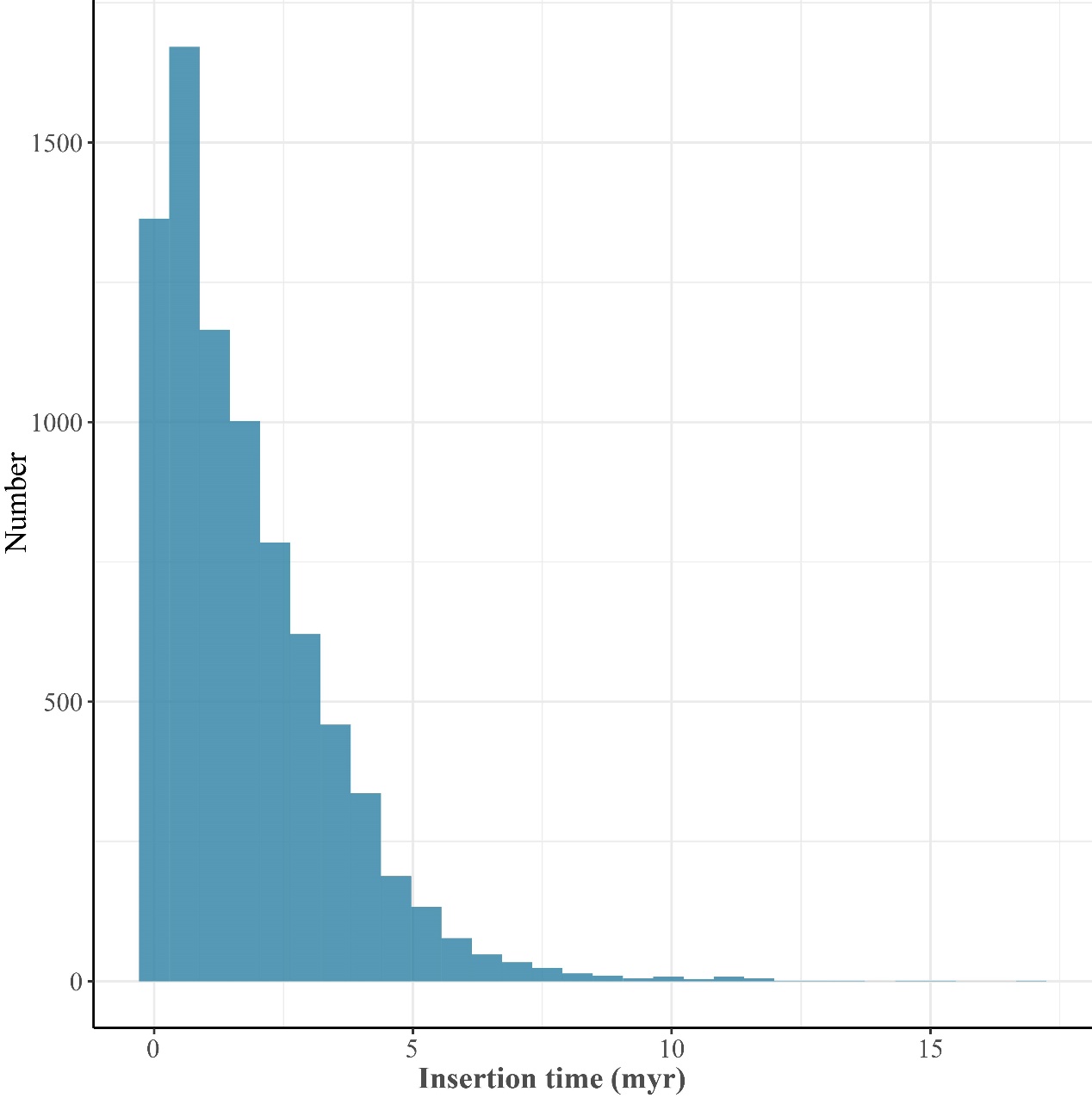


**Supplementary Figure 34. Insertion time of LTR-RTs (long terminal repeat-retrotransposons) in the *S. splendens* genome.**


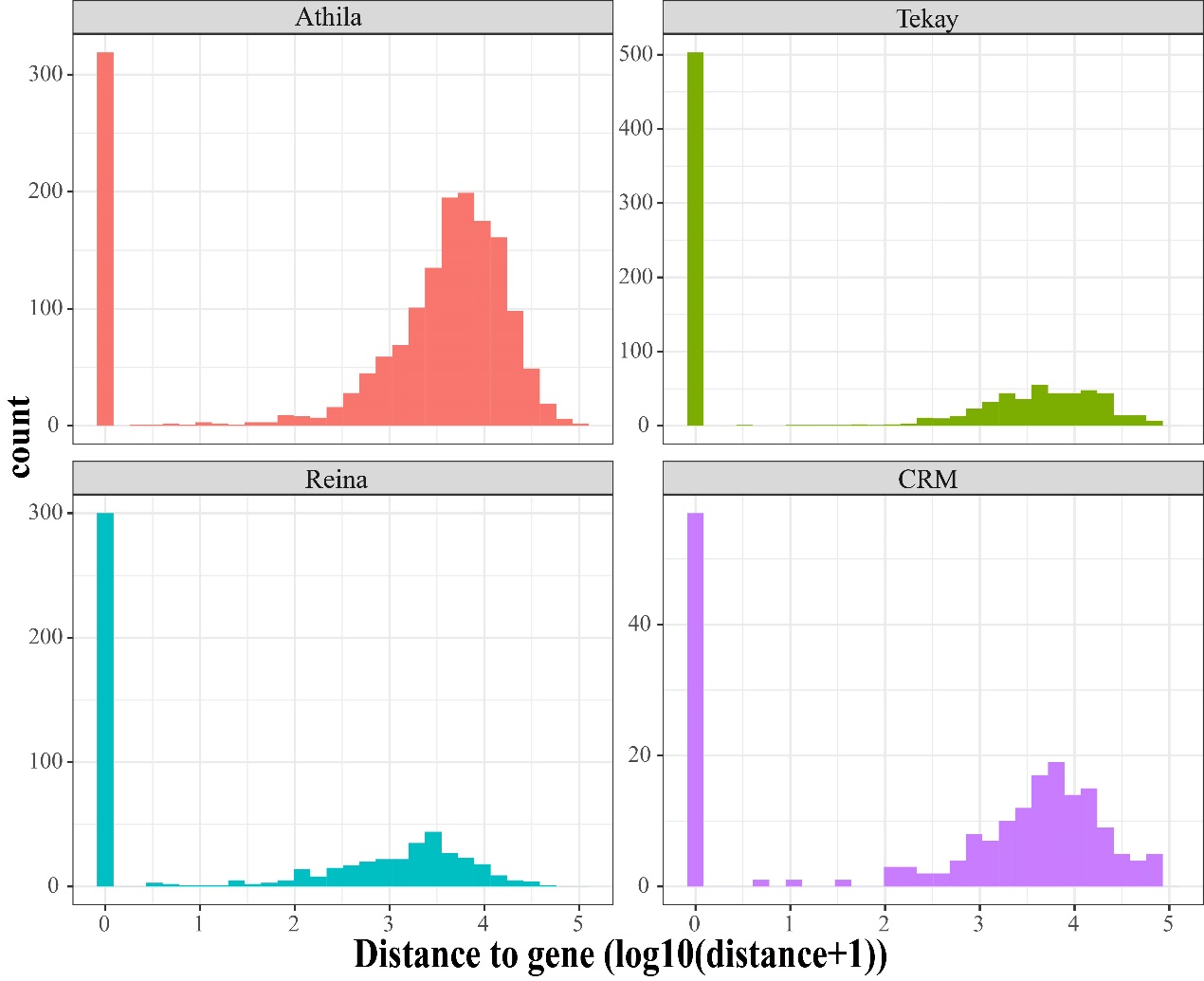


**Supplementary Figure 35.** **Gene proximity for different subgroups of *Gypsy* superfamily of LTR-RTs (long terminal repeat-retrotransposons) in the *S. splendens* genome*.***


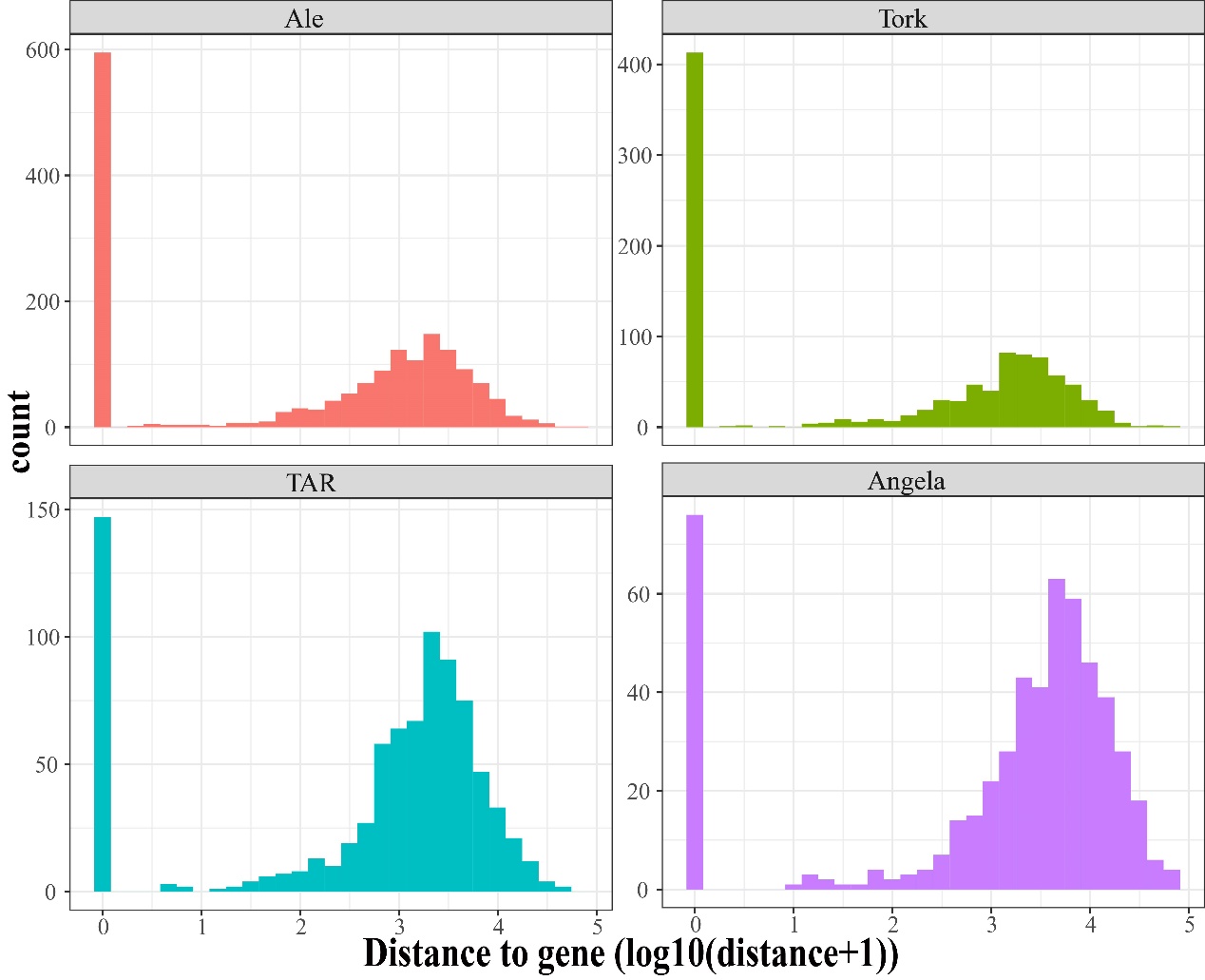


**Supplementary Figure 36.** **Gene proximity for different subgroups of *Copia* superfamily of LTR-RTs (long terminal repeat-retrotransposons) in the *S. splendens* genome*.***


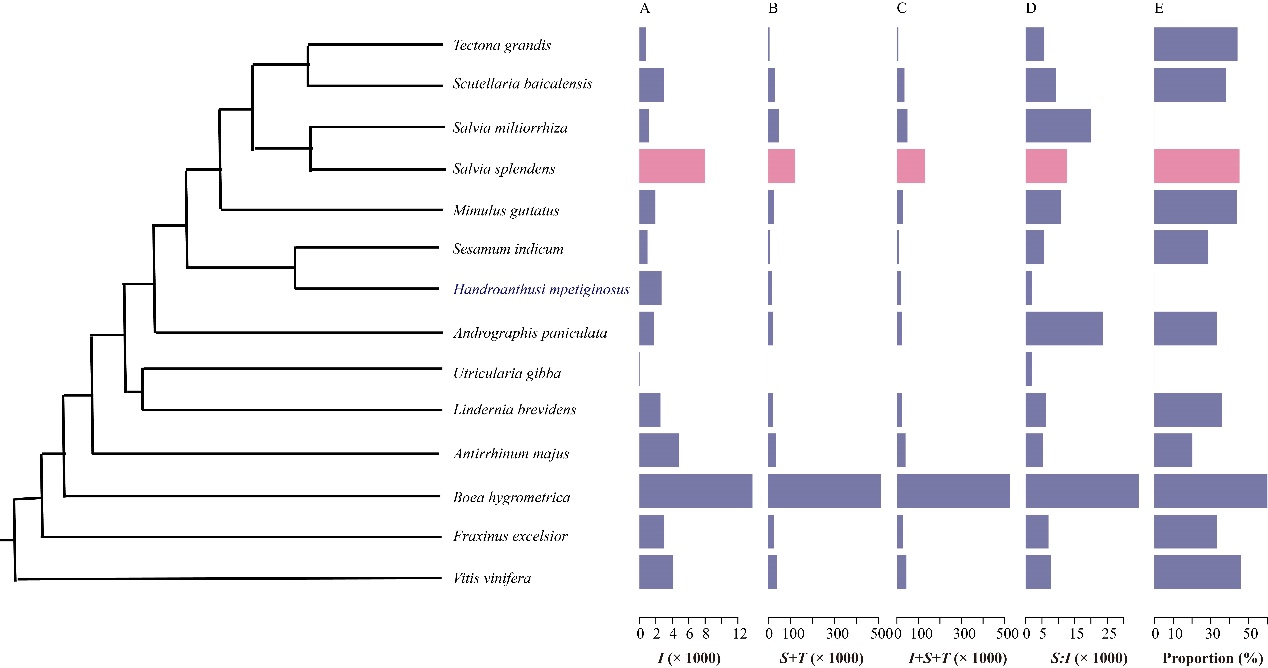


**Supplementary Figure 37. Birth and death of LTR-RTs (long terminal repeat-retrotransposons) in the genome of *S. splendens.*** (A) total numbers of LTR-RTs in genome; (B) comparison of *S* + *T* values among plant species; *S*, number of solo-LTRs; *T*, number of truncated LTR-RTs. (C) total numbers of intact LTR-RTs and traces of LTR-RT deaths; *I*, number of intact LTR-RTs. (D) ratios of solo-LTR to intact LTR-RT (*S*:*I*). (E) The proportions of LTR-RTs found in the clusters with high removal rates (filtered *S*:*I* ≥ 3).


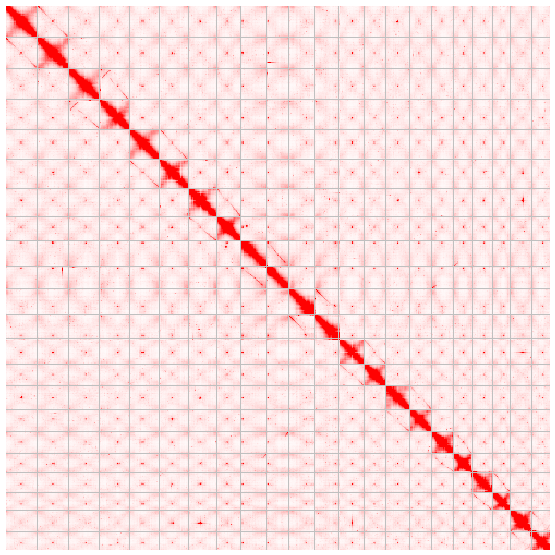


**Supplementary Figure 38. The Hi-C interaction of *S. splendens* pseudomolecules.**

**
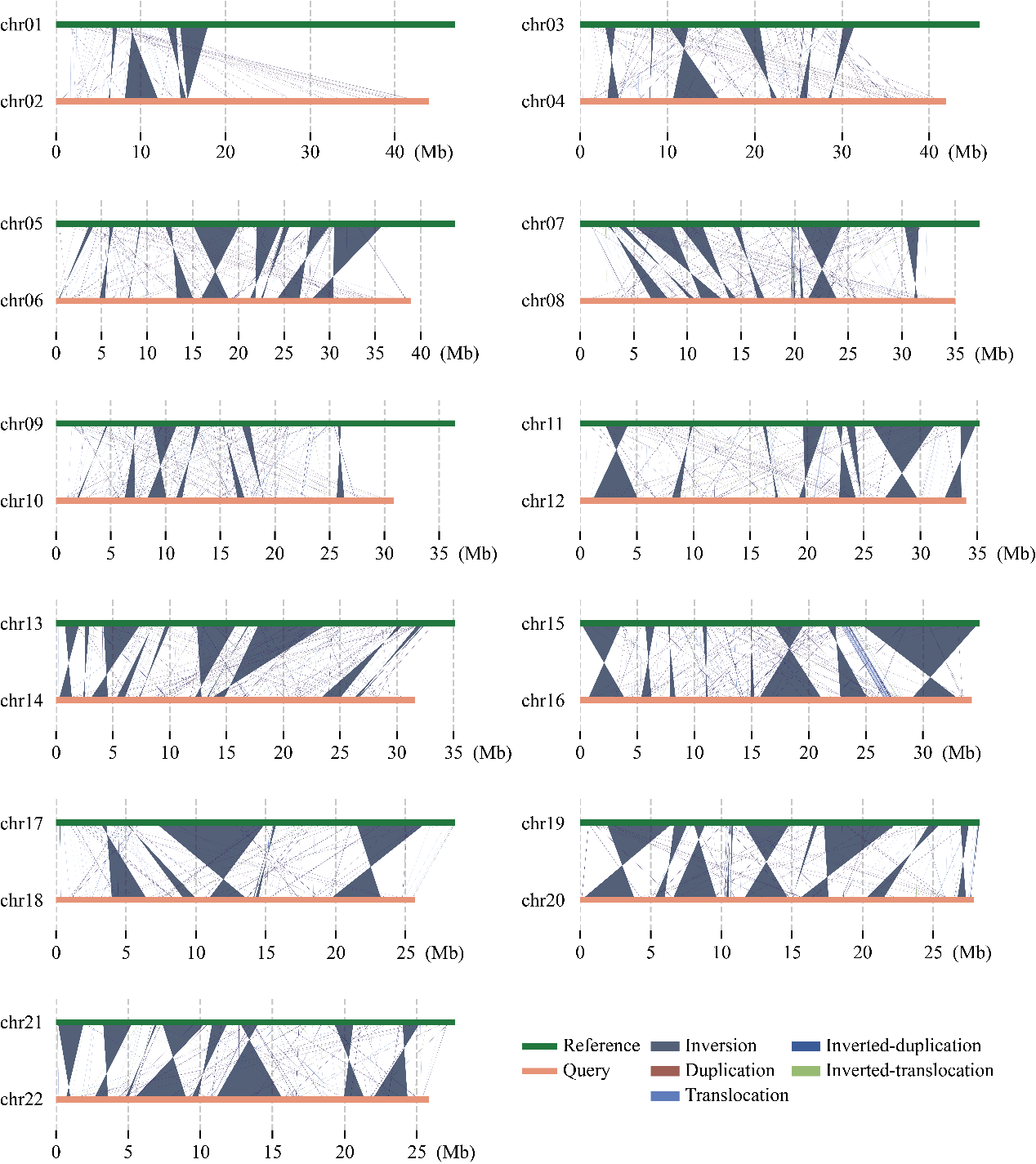
**

**Supplementary Figure 39. Structural variation between homoeologous chromosome pairs.**


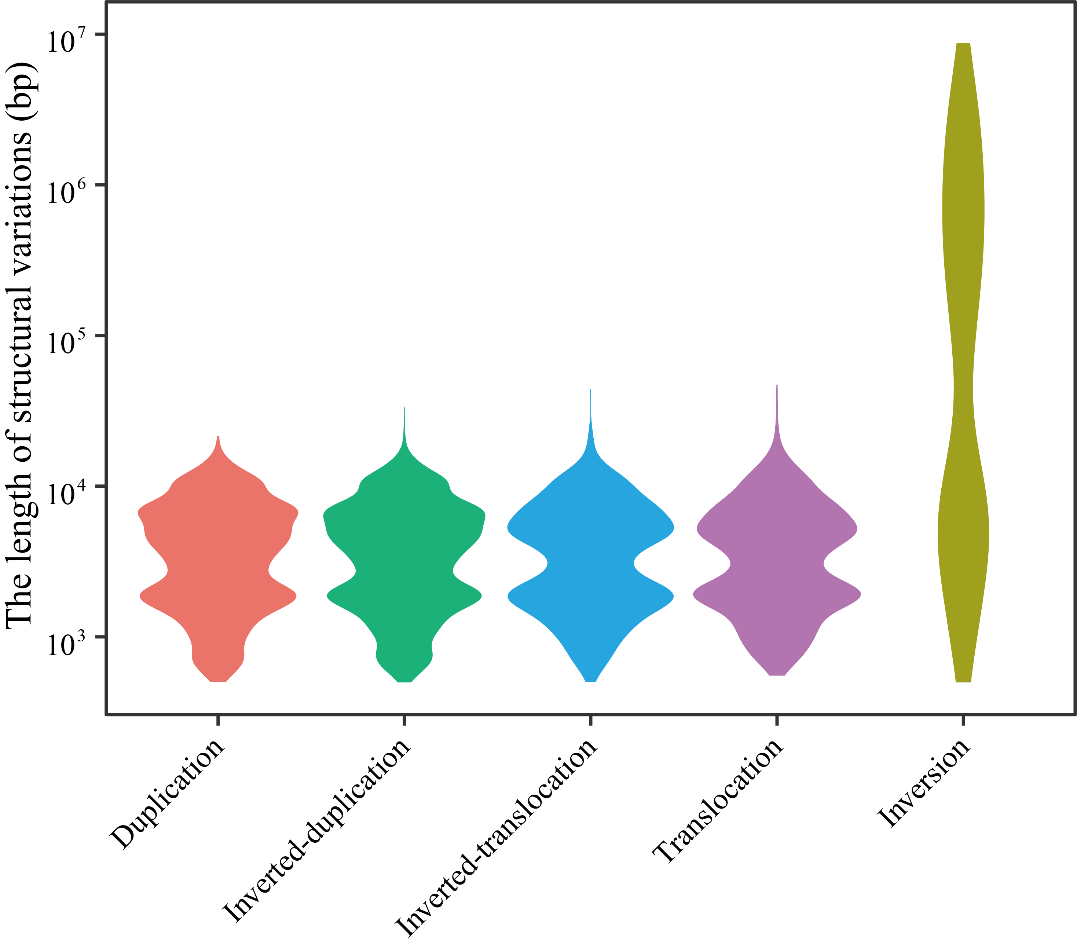


**Supplementary Figure 40. Size distributions of different types structural variation between homoeologus chromosome pairs.**


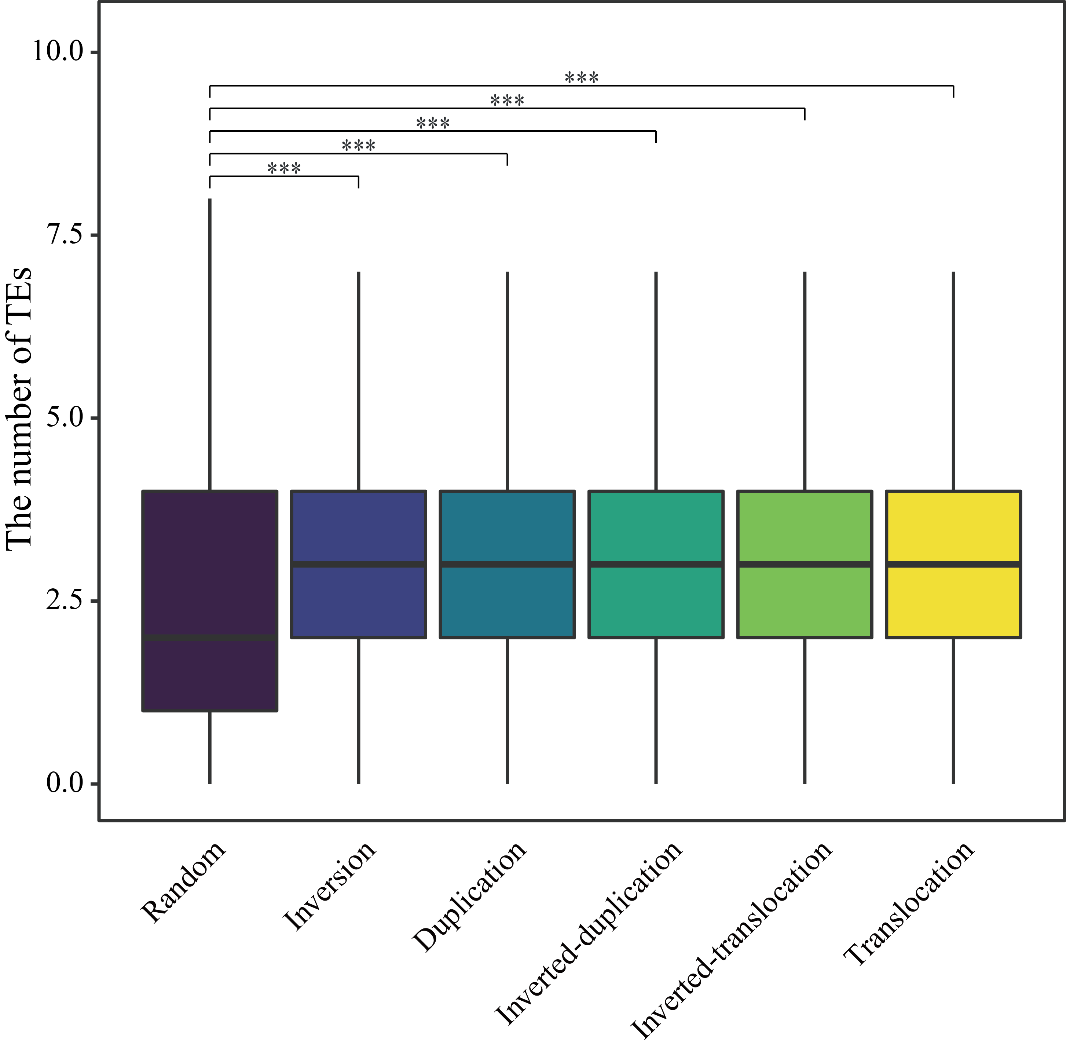


**Supplementary Figure 41. The number of different types of TEs in 4kb random regions and 2kb regions upstream and downstream of structural variation breakpoints.** Mann-Whitney-Wilcoxon test. * *p* < 0.05; ** *p* < 0.01; *** *p* < 0.001.


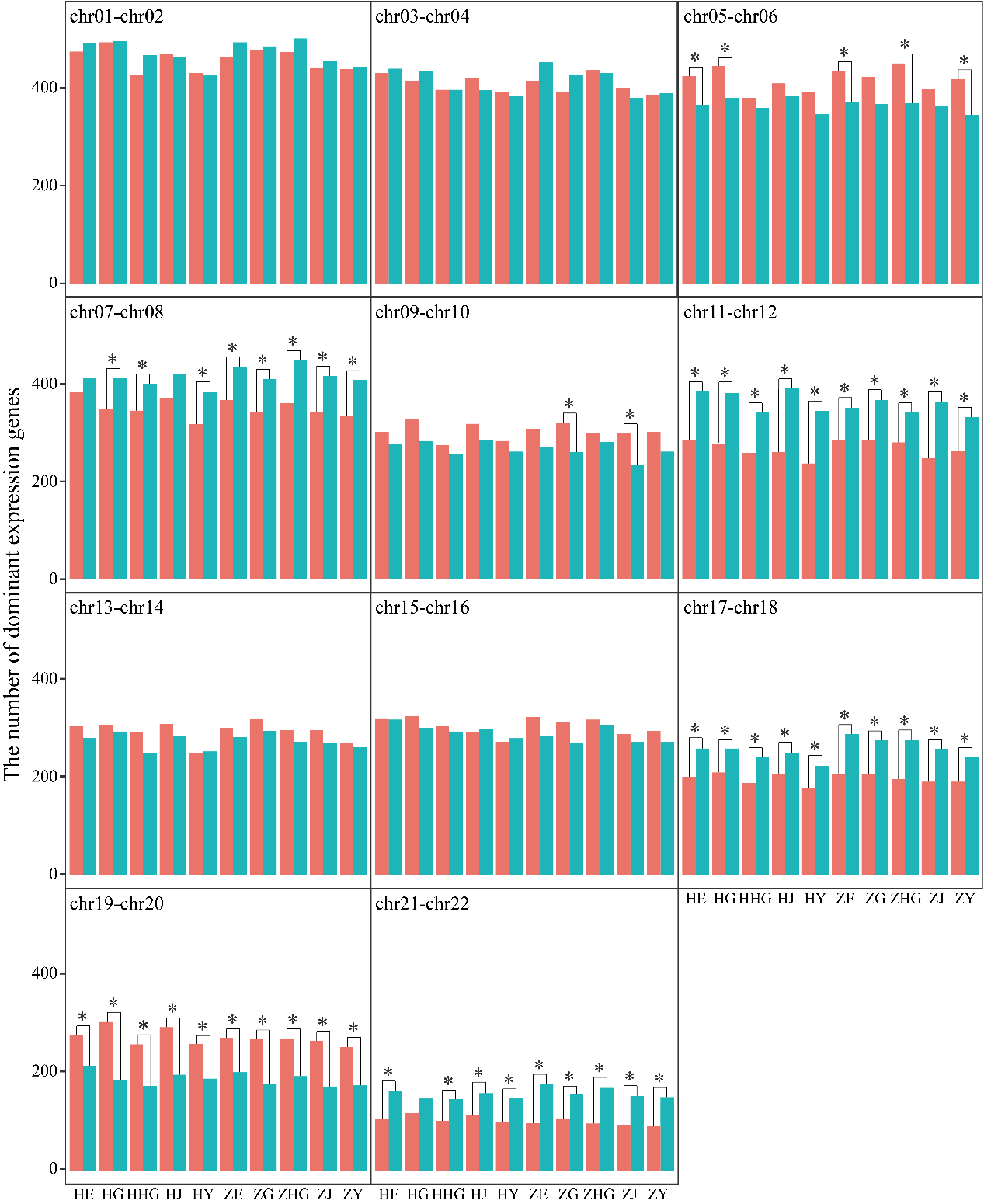


**Supplementary Figure 42. The number of dominant expression genes in homoeologous chromosomes.** y axis, the number of dominant expression genes (dominant expression indicates the genes’ expression values are at least 2-fold higher than their duplicated genes); x axis, different plant tissues. HE, calyx of red variety; HG, root of red variety; HHG, corolla of red variety; HJ, stem of red variety; HY, leave of red variety; ZE, calyx of purple variety; ZG, root of purple variety; ZHG, corolla of purple variety; ZJ, stem of purple variety; ZY, leave of purple variety. The statistical analysis was conducted by χ² test. **p* <0.05.

**Supplementary Table 1. Summary of PacBio and Illumina sequencing data generated in the present study.**

| **Instrument model** | **Design description** | **Sample** | **Raw reads (million)** | **Raw bases (Gb)** | **Clean reads (million)** | **Clean bases (Gb)** | **Clean Q20 (Gb)** | **Clean Q30 (Gb)** | **Average length (bp)** |
| --- | --- | --- | --- | --- | --- | --- | --- | --- | --- |
| PacBio RS II | leaf | huo1 | 4.882 | 31.044 | NA | NA | NA | NA | 6,358 |
| PacBio SEQUEL | leaf | huo1_1 | 3.976 | 34.918 | NA | NA | NA | NA | 8,782 |
| HiSeq X Ten | leaf | huo1 | 137.215 | 20.582 | 113.250(82.5%) | 15.578(75.7%) | 15.150(97.3%) | 14.374(92.3%) | 137.6 |
| HiSeq X Ten | leaf | huo1_1 | 128.316 | 19.247 | 104.962(81.8%) | 14.356(74.6%) | 13.933(97.1%) | 13.192(91.9%) | 136.8 |
| HiSeq X Ten | leaf | huo1 | NA | NA | 573.832 | 85.943 | 82.794(96.3%) | 78.046(90.8%) | 149.8 |
| HiSeq X Ten | root of red flower | HG_1 | 46.001 | 6.9 | 42.257(91.9%) | 6.219(90.1%) | 6.132(98.6%) | 5.948(95.6%) | 147.2 |
| HiSeq X Ten | root of red flower | HG_2 | 45.318 | 6.798 | 41.976(92.6%) | 6.180(90.9%) | 6.096(98.6%) | 5.918(95.8%) | 147.2 |
| HiSeq X Ten | root of red flower | HG_3 | 50.876 | 7.631 | 47.295(93.0%) | 6.961(91.2%) | 6.870(98.7%) | 6.678(95.9%) | 147.2 |
| HiSeq X Ten | stem of red flower | HJ_1 | 43.992 | 6.599 | 40.562(92.2%) | 5.979(90.6%) | 5.898(98.6%) | 5.724(95.7%) | 147.4 |
| HiSeq X Ten | stem of red flower | HJ_2 | 50.36 | 7.554 | 47.525(94.4%) | 7.019(92.9%) | 6.933(98.8%) | 6.746(96.1%) | 147.7 |
| HiSeq X Ten | stem of red flower | HJ_3 | 45.092 | 6.764 | 42.235(93.7%) | 6.233(92.1%) | 6.156(98.8%) | 5.988(96.1%) | 147.6 |
| HiSeq X Ten | leaf of red flower | HY_1 | 52.955 | 7.943 | 49.643(93.7%) | 7.329(92.3%) | 7.235(98.7%) | 7.030(95.9%) | 147.6 |
| HiSeq X Ten | leaf of red flower | HY_2 | 40.347 | 6.052 | 36.916(91.5%) | 5.438(89.9%) | 5.362(98.6%) | 5.200(95.6%) | 147.3 |
| HiSeq X Ten | leaf of red flower | HY_3 | 46.305 | 6.946 | 42.857(92.6%) | 6.315(90.9%) | 6.236(98.7%) | 6.063(96.0%) | 147.4 |
| HiSeq X Ten | calyx of red flower | HE_1 | 42.275 | 6.341 | 39.600(93.7%) | 5.849(92.2%) | 5.777(98.8%) | 5.621(96.1%) | 147.7 |
| HiSeq X Ten | calyx of red flower | HE_2 | 51.118 | 7.668 | 47.561(93.0%) | 7.017(91.5%) | 6.927(98.7%) | 6.733(95.9%) | 147.5 |
| HiSeq X Ten | calyx of red flower | HE_3 | 48.119 | 7.218 | 44.922(93.4%) | 6.637(92.0%) | 6.557(98.8%) | 6.381(96.1%) | 147.8 |
| HiSeq X Ten | corolla of red flower | HHG_1 | 43.315 | 6.497 | 39.148(90.4%) | 5.759(88.6%) | 5.675(98.5%) | 5.503(95.6%) | 147.1 |
| HiSeq X Ten | corolla of red flower | HHG_2 | 40.596 | 6.089 | 37.777(93.1%) | 5.576(91.6%) | 5.504(98.7%) | 5.349(95.9%) | 147.6 |
| HiSeq X Ten | corolla of red flower | HHG_3 | 42.145 | 6.322 | 38.495(91.3%) | 5.664(89.6%) | 5.587(98.6%) | 5.426(95.8%) | 147.1 |
| HiSeq X Ten | root of purple flower | ZG_1 | 47.533 | 7.13 | 43.191(90.9%) | 6.350(89.1%) | 6.261(98.6%) | 6.075(95.7%) | 147 |
| HiSeq X Ten | root of purple flower | ZG_2 | 39.287 | 5.893 | 35.916(91.4%) | 5.285(89.7%) | 5.212(98.6%) | 5.058(95.7%) | 147.1 |
| HiSeq X Ten | root of purple flower | ZG_3 | 43.434 | 6.515 | 39.325(90.5%) | 5.775(88.6%) | 5.691(98.6%) | 5.518(95.6%) | 146.8 |
| HiSeq X Ten | stem of purple flower | ZJ_1 | 38.073 | 5.711 | 34.928(91.7%) | 5.140(90.0%) | 5.071(98.7%) | 4.926(95.8%) | 147.2 |
| HiSeq X Ten | stem of purple flower | ZJ_2 | 53.805 | 8.071 | 49.244(91.5%) | 7.251(89.8%) | 7.154(98.7%) | 6.946(95.8%) | 147.3 |
| HiSeq X Ten | stem of purple flower | ZJ_3 | 44.904 | 6.736 | 41.815(93.1%) | 6.165(91.5%) | 6.089(98.8%) | 5.925(96.1%) | 147.4 |
| HiSeq X Ten | leaf of purple flower | ZY_1 | 40.155 | 6.023 | 36.737(91.5%) | 5.406(89.7%) | 5.335(98.7%) | 5.182(95.9%) | 147.1 |
| HiSeq X Ten | leaf of purple flower | ZY_2 | 36.852 | 5.528 | 33.531(91.0%) | 4.938(89.3%) | 4.871(98.6%) | 4.728(95.7%) | 147.3 |
| HiSeq X Ten | leaf of purple flower | ZY_3 | 53.735 | 8.06 | 49.854(92.8%) | 7.344(91.1%) | 7.252(98.7%) | 7.050(96.0%) | 147.3 |
| HiSeq X Ten | calyx of purple flower | ZE_1 | 38.872 | 5.831 | 36.226(93.2%) | 5.354(91.8%) | 5.289(98.8%) | 5.145(96.1%) | 147.8 |
| HiSeq X Ten | calyx of purple flower | ZE_2 | 41.611 | 6.242 | 37.163(89.3%) | 5.466(87.6%) | 5.387(98.6%) | 5.226(95.6%) | 147.1 |
| HiSeq X Ten | calyx of purple flower | ZE_3 | 46.011 | 6.902 | 41.988(91.3%) | 6.215(90.0%) | 6.126(98.6%) | 5.948(95.7%) | 148 |
| HiSeq X Ten | corolla of purple flower | ZHG_1 | 43.279 | 6.492 | 39.704(91.7%) | 5.854(90.2%) | 5.777(98.7%) | 5.614(95.9%) | 147.4 |
| HiSeq X Ten | corolla of purple flower | ZHG_2 | 42.844 | 6.427 | 41.038(95.8%) | 6.060(94.3%) | 5.996(98.9%) | 5.850(96.5%) | 147.7 |
| HiSeq X Ten | corolla of purple flower | ZHG_3 | 44.557 | 6.683 | 39.339(88.3%) | 5.804(86.8%) | 5.710(98.4%) | 5.530(95.3%) | 147.5 |

**Supplementary Table 2. Statistics of the different versions of the genome assembly of the *S. splendens*.**

| **Versions of assembly** | **Strategy** | **Assembled genome size (Mb)** | **Sequence number** | **N50** | **L50** | **Max. length (Mb)** | **Gene completeness (%)** |
| --- | --- | --- | --- | --- | --- | --- | --- |
| v0.1 | Canu | 808 | 2,306 | 2.06 Mb | 109 | 8.88 | 92.1 |
| v0.2 | Mecat | 790 | 2,597 | 856 Kb | 264 | 5.02 | 92 |
| v0.3 | Canu+Falcon | 827 | 4,480 | 1.09 Mb | 224 | 4.42 | NA |
| v0.4 | Canu+Smartdenovo | 747 | 1,999 | 728 Kb | 297 | 6.24 | NA |
| v1.0 | v0.1+arrow | 808 | 2,306 | 2.06 Mb | 109 | 8.88 | 92.2 |
| v1.1 | v1.0+Sspace×2+Gapcloser | 810 | 2,259/1,525 | 2.1/3.12 Mb | 106/73 | 10.8/12.9 | 92 |
| v1.2 | v1.1+Arow+Pilon×2 | 809 | 2,204/1,525 | 2.26/3.12 Mb | 100/73 | 10.8/12.9 | 92.2 |
| v1.3 | v1.2+Hi-C+Gapclose+  Polish×3+Remove Redundant | 807 | 1,655/1,184 | 3.77/35.13 Mb | 100/73 | 12.9/47.1 | 92 |

| **Supplementary Table 3. Summary of BUSCOs search results.** | | |
| --- | --- | --- |
| **Type** | **Number** | **Percentage (%)** |
| Complete BUSCOs | 1324 | 92.0 |
| Complete and Single-copy BUSCOs (S) | 397 | 27.6 |
| Complete and Duplicated BUSCOs (D) | 927 | 64.4 |
| Missing BUSCOs (M) | 116 | 8.0 |
| **Total BUSCO Groups Searched** | **1,440** | **100.0** |

**Supplementary Table 4. Summary of RNAs.**

| **Types** | **Copy numbers** |
| --- | --- |
| mRNA | 56,267 |
| rRNA/28S | 50 |
| rRNA/18S | 14 |
| rRNA/5S | 454 |
| lncRNA | 28,993 |
| tRNA | 1,541 |
| ncRNA | 1,170 |
| **Total** | **88,489** |

**Supplementary Table 5. Summary of transcription factor genes.**

| **TF families** | **Numbers** | **TF families** | **Numbers** | **TF families** | **Numbers** |
| --- | --- | --- | --- | --- | --- |
| AP2 | 99 | HSF | 59 | GATA | 60 |
| ARF | 57 | LBD | 65 | GeBP | 20 |
| ARR-B | 19 | LFY | 5 | GRAS | 134 |
| B3 | 160 | LSD | 12 | GRF | 23 |
| BBR-BPC | 16 | MIKC_MADS | 17 | HB-other | 30 |
| BES1 | 20 | M-type_MADS | 59 | HB-PHD | 4 |
| bHLH | 325 | MYB | 281 | HD-ZIP | 118 |
| bZIP | 154 | MYB_related | 163 | HRT-like | 2 |
| C2H2 | 221 | NAC | 192 | TCP | 73 |
| C3H | 103 | NF-X1 | 2 | Trihelix | 69 |
| CAMTA | 12 | NF-YA | 20 | VOZ | 5 |
| CO-like | 34 | NF-YB | 32 | Whirly | 5 |
| CPP | 17 | NF-YC | 16 | WOX | 33 |
| DBB | 18 | Nin-like | 31 | WRKY | 160 |
| Dof | 92 | S1Fa-like | 3 | YABBY | 16 |
| E2F/DP | 20 | SAP | 5 | ZF-HD | 49 |
| EIL | 21 | SBP | 46 | G2-like | 114 |
| ERF | 321 | SRS | 16 | TALE | 50 |
| FAR1 | 94 | STAT | 1 |  |  |

**Supplementary Table 6. Gene ontology enrichment analysis among unique gene families in *S. splendens*.**

| **ID** | **Description** | **Category** | **pvalue** | **p.adjust** | **qvalue** |
| --- | --- | --- | --- | --- | --- |
| GO:0015074 | DNA integration | biological process | 1.08E-79 | 3.70E-77 | 2.69E-77 |
| GO:0008270 | zinc ion binding | molecular function | 2.66E-54 | 4.57E-52 | 3.32E-52 |
| GO:0003676 | nucleic acid binding | molecular function | 4.24E-49 | 4.85E-47 | 3.52E-47 |
| GO:0019760 | glucosinolate metabolic process | biological process | 3.03E-32 | 2.60E-30 | 1.89E-30 |
| GO:0009772 | photosynthetic electron transport in photosystem II | biological process | 9.07E-32 | 6.22E-30 | 4.53E-30 |
| GO:0045261 | proton-transporting ATP synthase complex catalytic core F(1) | cellular component | 2.00E-31 | 1.14E-29 | 8.30E-30 |
| GO:0015986 | ATP synthesis coupled proton transport | biological process | 3.64E-31 | 1.78E-29 | 1.30E-29 |
| GO:0046933 | proton-transporting ATP synthase activity rotational mechanism | molecular function | 8.95E-30 | 3.84E-28 | 2.79E-28 |
| GO:0043900 | regulation of multi-organism process | biological process | 1.92E-28 | 7.33E-27 | 5.33E-27 |
| GO:0009595 | detection of biotic stimulus | biological process | 4.08E-28 | 1.40E-26 | 1.02E-26 |
| GO:0046403 | polynucleotide 3'-phosphatase activity | molecular function | 1.04E-27 | 3.24E-26 | 2.36E-26 |
| GO:0009773 | photosynthetic electron transport in photosystem I | biological process | 1.72E-26 | 4.92E-25 | 3.58E-25 |
| GO:0010319 | stromule | cellular component | 2.98E-24 | 7.87E-23 | 5.72E-23 |
| GO:0010310 | regulation of hydrogen peroxide metabolic process | biological process | 9.33E-23 | 2.29E-21 | 1.66E-21 |
| GO:0009697 | salicylic acid biosynthetic process | biological process | 1.75E-22 | 4.00E-21 | 2.91E-21 |
| GO:0010287 | plastoglobule | cellular component | 1.94E-22 | 4.16E-21 | 3.02E-21 |
| GO:0000165 | MAPK cascade | biological process | 6.93E-21 | 1.40E-19 | 1.02E-19 |
| GO:0009862 | systemic acquired resistance salicylic acid mediated signaling pathway | biological process | 9.56E-20 | 1.82E-18 | 1.33E-18 |
| GO:0010207 | photosystem II assembly | biological process | 1.63E-19 | 2.94E-18 | 2.14E-18 |
| GO:0015995 | chlorophyll biosynthetic process | biological process | 3.92E-19 | 6.72E-18 | 4.89E-18 |
| GO:0010363 | regulation of plant-type hypersensitive response | biological process | 6.44E-19 | 1.05E-17 | 7.65E-18 |
| GO:0019252 | starch biosynthetic process | biological process | 7.41E-19 | 1.15E-17 | 8.40E-18 |
| GO:0006612 | protein targeting to membrane | biological process | 2.19E-18 | 3.27E-17 | 2.38E-17 |

**Supplementary Table 7. Summary of repeat content.**

| **Family** | **Element** | **Number** | **Length (bp)** | **Percent of genome (%)** | **Mean length (bp)** |
| --- | --- | --- | --- | --- | --- |
| LTR | Cassandra | 311 | 39,915 | 0.00 | 128.34 |
|  | Caulimovirus | 1,469 | 2,740,938 | 0.34 | 1865.85 |
|  | Copia | 64,397 | 70,186,773 | 8.70 | 1089.91 |
|  | Gypsy | 116,204 | 141,979,589 | 17.59 | 1221.81 |
|  | Pao | 1,115 | 229,861 | 0.03 | 206.15 |
| LINE | CRE | 1,444 | 157,562 | 0.02 | 109.11 |
|  | I-Jockey | 1,079 | 232,828 | 0.03 | 215.78 |
|  | L1 | 13,823 | 12,442,546 | 1.54 | 900.13 |
|  | L1-Tx1 | 5,399 | 4,219,168 | 0.52 | 781.47 |
|  | L2 | 1,163 | 164,195 | 0.02 | 141.18 |
| SINE |  | 2,182 | 268,378 | 0.03 | 123.00 |
| DNA | Academ-1 | 230 | 93,490 | 0.01 | 406.48 |
|  | CMC-EnSpm | 3,231 | 1,017,758 | 0.13 | 315.00 |
|  | Ginger | 8,545 | 1,580,411 | 0.20 | 184.95 |
|  | MULE-MuDR | 6,118 | 1,794,979 | 0.22 | 293.39 |
|  | MuLE-MuDR | 24,226 | 9,053,996 | 1.12 | 373.73 |
|  | Novosib | 3,079 | 2,194,918 | 0.27 | 712.87 |
|  | PIF-Harbinger | 71,890 | 46,173,731 | 5.72 | 642.28 |
|  | PIF-ISL2EU | 180 | 69,942 | 0.01 | 388.57 |
|  | TcMar-Mogwai | 446 | 339,615 | 0.04 | 761.47 |
|  | TcMar-Stowaway | 69,797 | 11,438,212 | 1.42 | 163.88 |
|  | TcMar-Tc1 | 418 | 182,289 | 0.02 | 436.10 |
|  | Zisupton | 17,939 | 5,415,603 | 0.67 | 301.89 |
|  | hAT-Ac | 42,296 | 15,954,333 | 1.98 | 377.21 |
|  | hAT-Tag1 | 4,051 | 1,058,610 | 0.13 | 261.32 |
|  | hAT-Tip100 | 3,363 | 1,231,131 | 0.15 | 366.08 |
| RC | Helitron | 1,234 | 670,979 | 0.08 | 543.74 |
| Unknown |  | 427,843 | 119,120,952 | 14.76 | 278.42 |
| Satellite |  | 544 | 62,059 | 0.01 | 114.08 |
| Simple_repeat |  | 148,130 | 6,685,468 | 0.83 | 45.13 |
| Low_complexity |  | 27,573 | 1,384,646 | 0.17 | 50.22 |
| snRNA |  | 208 | 36,271 | 0.00 | 174.38 |
| **total** |  | **1,076,141** | **459,518,768** | **56.94** | **427.01** |

**Supplementary Table 8. Summary of centromeric repeat array (CenT) composition in *S. splendens* genome.**

| **Chromosomes** | **CenT array start** | **CenT array end** | **CenT array length (Mb)** | **Number of LINE1 repeats** |
| --- | --- | --- | --- | --- |
| chr01 | 23,900,000 | 24,700,000 | 0.8 | 343 |
| chr02 | 21,900,000 | 22,700,000 | 0.8 | 345 |
| chr03 | 25,300,000 | 26,000,000 | 0.7 | 207 |
| chr04 | 24,400,000 | 25,300,000 | 0.9 | 257 |
| chr05 | 24,600,000 | 25,800,000 | 1.2 | 573 |
| chr06 | 22,300,000 | 22,600,000 | 0.3 | 142 |
| chr07 | 19,600,000 | 19,900,000 | 0.3 | 93 |
| chr08 | 19,500,000 | 20,200,000 | 0.7 | 290 |
| chr09 | 35,000,000 | 36,200,000 | 1.2 | 565 |
| chr10 | 29,000,000 | 29,700,000 | 0.7 | 352 |
| chr11 | 16,300,000 | 17,000,000 | 0.7 | 333 |
| chr12 | 17,100,000 | 17,800,000 | 0.7 | 303 |
| chr13 | 16,800,000 | 17,700,000 | 0.9 | 447 |
| chr14 | 12,900,000 | 13,300,000 | 0.4 | 123 |
| chr15 | 23,500,000 | 24,100,000 | 0.6 | 227 |
| chr16 | 27,700,000 | 28,200,000 | 0.5 | 212 |
| chr17 | 15,500,000 | 15,900,000 | 0.4 | 341 |
| chr18 | 14,000,000 | 14,900,000 | 0.9 | 388 |
| chr19 | 10,300,000 | 10,900,000 | 0.6 | 241 |
| chr20 | 10,000,000 | 10,900,000 | 0.9 | 406 |
| chr21 | 13,600,000 | 14,300,000 | 0.7 | 245 |
| chr22 | 10,900,000 | 11,600,000 | 0.7 | 266 |

**Supplementary Table 9. Summary of structural variations between homoeologous chromosomes.**

| **Chr.** | **Duplications** | | **Inversions** | | **Translocations** | | **Inverted-duplications** | | **Inverted-translocations** | | **Sum** | | **Length of chromosomes (bp)** | **Percent (%)** |
| --- | --- | --- | --- | --- | --- | --- | --- | --- | --- | --- | --- | --- | --- | --- |
|  | **Number** | **Size (bp)** | **Number** | **Size (bp)** | **Number** | **Size (bp)** | **Number** | **Size (bp)** | **Number** | **Size (bp)** | **Number** | **Size (bp)** |  |  |
| chr01 | 177 | 673860 | 10 | 5519823 | 53 | 217193 | 166 | 706882 | 51 | 202577 | 457 | 7320335 | 47130199 | 15.53% |
| chr02 |  | 671,672 |  | 5,075,616 |  | 210,462 |  | 711,171 |  | 203,421 |  | 6872342 | 43,663,975 | 15.74% |
| chr03 | 322 | 1,396,770 | 17 | 8,325,928 | 89 | 375,925 | 335 | 1,440,546 | 94 | 387,763 | 857 | 11926932 | 45,750,320 | 26.07% |
| chr04 |  | 1,408,962 |  | 9,270,091 |  | 376,532 |  | 1,432,120 |  | 391,552 |  | 12879257 | 41,582,922 | 30.97% |
| chr05 | 326 | 1,426,772 | 16 | 18,192,372 | 102 | 385,636 | 338 | 1,389,964 | 96 | 349,102 | 878 | 21743846 | 43,775,046 | 49.67% |
| chr06 |  | 1,414,140 |  | 12,131,793 |  | 386,176 |  | 1,387,135 |  | 351,252 |  | 15670496 | 38,537,683 | 40.66% |
| chr07 | 321 | 1,377,115 | 17 | 13,149,049 | 110 | 488,592 | 321 | 1,442,975 | 81 | 412,047 | 850 | 16869778 | 37,237,137 | 45.30% |
| chr08 |  | 1,359,369 |  | 9,443,583 |  | 477,063 |  | 1,437,547 |  | 384,718 |  | 13102280 | 34,700,264 | 37.76% |
| chr09 | 294 | 1,350,732 | 15 | 4,630,595 | 119 | 514,361 | 350 | 1,509,174 | 105 | 433,985 | 883 | 8438847 | 36,474,241 | 23.14% |
| chr10 |  | 1,340,481 |  | 5,200,849 |  | 510,509 |  | 1,498,479 |  | 422,405 |  | 8972723 | 30,561,544 | 29.36% |
| chr11 | 339 | 1,470,112 | 15 | 13,151,346 | 88 | 392,710 | 331 | 1,387,649 | 104 | 496,780 | 877 | 16898597 | 35,206,759 | 48.00% |
| chr12 |  | 1,469,030 |  | 12,038,359 |  | 405,345 |  | 1,406,131 |  | 485,733 |  | 15804598 | 33,758,164 | 46.82% |
| chr13 | 333 | 1,401,968 | 21 | 16,494,205 | 76 | 342,153 | 362 | 1,679,903 | 78 | 334,550 | 870 | 20252779 | 35,138,742 | 57.64% |
| chr14 |  | 1,406,864 |  | 8,283,746 |  | 352,444 |  | 1,665,965 |  | 334,087 |  | 12043106 | 31,325,185 | 38.45% |
| chr15 | 288 | 1,432,002 | 16 | 19,282,392 | 74 | 481,327 | 284 | 1,470,868 | 49 | 269,856 | 711 | 22936445 | 34,957,497 | 65.61% |
| chr16 |  | 1,439,552 |  | 17,920,088 |  | 487,025 |  | 1,465,420 |  | 262,477 |  | 21574562 | 34,015,037 | 63.43% |
| chr17 | 228 | 1,020,817 | 14 | 13,851,543 | 59 | 244,704 | 231 | 938,277 | 53 | 232,819 | 585 | 16288160 | 28,571,943 | 57.01% |
| chr18 |  | 1,035,110 |  | 10,447,480 |  | 259,634 |  | 932,445 |  | 231,866 |  | 12906535 | 25,457,334 | 50.70% |
| chr19 | 234 | 1,096,389 | 14 | 17,195,942 | 41 | 164,208 | 218 | 975,907 | 45 | 188,498 | 552 | 19620944 | 28,282,137 | 69.38% |
| chr20 |  | 1,109,331 |  | 15,293,295 |  | 163,247 |  | 955,249 |  | 189,755 |  | 17710877 | 27,682,662 | 63.98% |
| chr21 | 191 | 858,932 | 12 | 12,093,477 | 54 | 229,572 | 224 | 1,125,019 | 35 | 174,537 | 516 | 14481537 | 27,667,306 | 52.34% |
| chr22 |  | 868,337 |  | 14,365,715 |  | 224,815 |  | 1,095,996 |  | 173,721 |  | 16728584 | 25,615,879 | 65.31% |
| sum | 3,053 | 27,028,317 | 167 | 261,357,287 | 865 | 7,689,633 | 3,160 | 28,054,822 | 791 | 6,913,501 | 8036 | 331,043,560 | 767,091,976 | 43.16% |

**Supplementary Table 10. Statistics of structural variations in *S. splendens* genome.**

|  | **Size (bp)** | **Number** | **Min. size (bp)** | **Max. size (bp)** | **Median size (bp)** | | |
| --- | --- | --- | --- | --- | --- | --- | --- |
| **Duplications** | 27,028,317 | 3,053 | 500 | 24,156 | | 3,570 |  |
| **Inversions** | 261,357,287 | 167 | 595 | 10,411,366 | | 74,066 |  |
| **Translocations** | 7,689,633 | 865 | 553 | 47,215 | | 3,357 |  |
| **Inverted-duplications** | 28,054,822 | 3,160 | 499 | 33,660 | | 3,598 |  |
| **Inverted-translocations** | 6,913,501 | 791 | 504 | 43,917 | | 3,408 |  |

**Supplementary Table 11. The number of different types of repeating elements in structural variation breakpoints.**

|  | **The number of repeating elements near the breakpoints** | **The number of repeating elements in 22 chromosomes** | **Percent (%)** |
| --- | --- | --- | --- |
| **DNA** | 25,089 | 259,139 | 9.68 |
| **LINE** | 2,352 | 22,908 | 10.27 |
| **LTR** | 26,606 | 186,380 | 14.28 |
| **RC** | 96 | 1,234 | 7.78 |
| **SINE** | 134 | 427,843 | 6.14 |

**Supplementary Table 12. The number of differently expressed genes in structural variations.**

|  | **The number of genes** | **The number of genes in synetic blocks** | **The number of genes had different expression** | **Percent (%)** |
| --- | --- | --- | --- | --- |
| **Duplications** | 450 | 384 | 216 | 56.25% |
| **Inversions** | 16,776 | 14,804 | 6,638 | 44.84% |
| **Inverted-duplications** | 387 | 326 | 204 | 62.58% |
| **Inverted-translocations** | 173 | 158 | 72 | 45.57% |
| **Translocations** | 245 | 206 | 100 | 48.54% |
| **Sum** | 18,031 | 15,878 | 7,230 | 45.53% |

**Supplementary Table 13. Gene ontology enrichment analysis among stable homoeologs in *S. splendens*.**

| **ID** | **Description** | **Category** | **pvalue** | **p.adjust** | **qvalue** |
| --- | --- | --- | --- | --- | --- |
| GO:0044822 | poly(A) RNA binding | molecular function | 5.99E-28 | 8.72E-25 | 6.59E-25 |
| GO:0008380 | RNA splicing | biological process | 7.21E-28 | 8.72E-25 | 6.59E-25 |
| GO:0005654 | nucleoplasm | cellular component | 4.98E-27 | 4.02E-24 | 3.03E-24 |
| GO:0003723 | RNA binding | molecular function | 1.26E-26 | 7.60E-24 | 5.74E-24 |
| GO:0006397 | mRNA processing | biological process | 3.21E-26 | 1.55E-23 | 1.17E-23 |
| GO:0005730 | nucleolus | cellular component | 8.74E-24 | 3.53E-21 | 2.66E-21 |
| GO:0000166 | nucleotide binding | molecular function | 2.16E-18 | 7.45E-16 | 5.63E-16 |
| GO:0010467 | gene expression | biological process | 1.61E-17 | 4.88E-15 | 3.69E-15 |
| GO:0005681 | spliceosomal complex | cellular component | 4.24E-17 | 1.14E-14 | 8.61E-15 |
| GO:0000398 | mRNA splicing via spliceosome | biological process | 9.61E-17 | 2.33E-14 | 1.76E-14 |
| GO:0000502 | proteasome complex | cellular component | 4.18E-15 | 9.20E-13 | 6.95E-13 |
| GO:0010228 | vegetative to reproductive phase transition of meristem | biological process | 4.01E-13 | 8.09E-11 | 6.11E-11 |
| GO:0022626 | cytosolic ribosome | cellular component | 1.24E-12 | 2.30E-10 | 1.74E-10 |
| GO:0006367 | transcription initiation from RNA polymerase II promoter | biological process | 1.32E-11 | 2.28E-09 | 1.72E-09 |
| GO:0006886 | intracellular protein transport | biological process | 2.10E-11 | 3.38E-09 | 2.55E-09 |
| GO:0043161 | proteasome-mediated ubiquitin-dependent protein catabolic process | biological process | 2.52E-11 | 3.80E-09 | 2.87E-09 |
| GO:0006511 | ubiquitin-dependent protein catabolic process | biological process | 2.67E-11 | 3.80E-09 | 2.87E-09 |
| GO:0003676 | nucleic acid binding | molecular function | 3.64E-11 | 4.89E-09 | 3.70E-09 |
| GO:0000184 | nuclear-transcribed mRNA catabolic process nonsense-mediated decay | biological process | 6.28E-11 | 8.00E-09 | 6.04E-09 |
| GO:0005635 | nuclear envelope | cellular component | 9.62E-11 | 1.16E-08 | 8.79E-09 |
| GO:0015031 | protein transport | biological process | 1.08E-10 | 1.25E-08 | 9.43E-09 |
| GO:0000956 | nuclear-transcribed mRNA catabolic process | biological process | 2.31E-10 | 2.44E-08 | 1.84E-08 |
| GO:0016192 | vesicle-mediated transport | biological process | 2.40E-10 | 2.44E-08 | 1.84E-08 |

**Supplementary Table 14. Gene ontology enrichment analysis among dynamic homoeologs in *S. splendens*.**

| **ID** | **Description** | **Category** | **pvalue** | **p.adjust** | **qvalue** |
| --- | --- | --- | --- | --- | --- |
| GO:0020037 | heme binding | molecular function | 9.17E-20 | 1.53E-16 | 1.32E-16 |
| GO:0005576 | extracellular region | cellular component | 1.43E-19 | 1.53E-16 | 1.32E-16 |
| GO:0005506 | iron ion binding | molecular function | 3.34E-17 | 2.39E-14 | 2.05E-14 |
| GO:0004497 | monooxygenase activity | molecular function | 8.69E-17 | 3.73E-14 | 3.20E-14 |
| GO:0009813 | flavonoid biosynthetic process | biological process | 1.48E-14 | 5.29E-12 | 4.55E-12 |
| GO:0016758 | transferase activity transferring hexosyl groups | molecular function | 3.34E-11 | 1.02E-08 | 8.80E-09 |
| GO:0080043 | quercetin 3-O-glucosyltransferase activity | molecular function | 5.64E-11 | 1.34E-08 | 1.15E-08 |
| GO:0080044 | quercetin 7-O-glucosyltransferase activity | molecular function | 5.64E-11 | 1.34E-08 | 1.15E-08 |
| GO:0035251 | UDP-glucosyltransferase activity | molecular function | 2.76E-10 | 5.92E-08 | 5.09E-08 |
| GO:0052696 | flavonoid glucuronidation | biological process | 3.10E-10 | 6.05E-08 | 5.20E-08 |
| GO:0009809 | lignin biosynthetic process | biological process | 3.50E-10 | 6.25E-08 | 5.37E-08 |
| GO:0016747 | transferase activity transferring acyl groups other than amino-acyl groups | molecular function | 3.17E-09 | 4.79E-07 | 4.12E-07 |
| GO:0019500 | cyanide catabolic process | biological process | 3.35E-09 | 4.79E-07 | 4.12E-07 |
| GO:0051410 | detoxification of nitrogen compound | biological process | 3.35E-09 | 4.79E-07 | 4.12E-07 |
| GO:0016042 | lipid catabolic process | biological process | 8.48E-09 | 1.06E-06 | 9.15E-07 |
| GO:0019499 | cyanide metabolic process | biological process | 8.91E-09 | 1.06E-06 | 9.15E-07 |
| GO:0010333 | terpene synthase activity | molecular function | 2.49E-08 | 2.81E-06 | 2.42E-06 |
| GO:0009718 | anthocyanin-containing compound biosynthetic process | biological process | 3.16E-08 | 3.38E-06 | 2.91E-06 |
| GO:0047427 | cyanoalanine nitrilase activity | molecular function | 4.13E-08 | 4.03E-06 | 3.46E-06 |
| GO:0047558 | 3-cyanoalanine hydratase activity | molecular function | 4.13E-08 | 4.03E-06 | 3.46E-06 |
| GO:0031408 | oxylipin biosynthetic process | biological process | 9.72E-08 | 9.06E-06 | 7.79E-06 |
| GO:0018822 | nitrile hydratase activity | molecular function | 1.88E-07 | 1.61E-05 | 1.38E-05 |
| GO:0080061 | indole-3-acetonitrile nitrilase activity | molecular function | 1.88E-07 | 1.61E-05 | 1.38E-05 |

**Supplementary Table 15. Co-expression based on module assignment of homoeologs for different varieties.**

|  |  | **Category (count)** | | | **Category (%)** | | |
| --- | --- | --- | --- | --- | --- | --- | --- |
| **Network** | **The number of modules** | **Same** | **Similar** | **Divergent** | **Same** | **Similar** | **Divergent** |
| **red variety** | 26 | 4962 | 4837 | 4824 | 33.93 | 33.08 | 32.99 |
| **purple variety** | 30 | 4687 | 4814 | 4812 | 32.75 | 33.63 | 33.62 |
